# Syntheses, Characterizations, and Inhibition Activities Against Coxsackievirus B3 of Iodobenzoic Hydrazide Functionalized Hexamolybdates

**DOI:** 10.3389/fchem.2022.841151

**Published:** 2022-03-17

**Authors:** Long-Sheng Wang, Chao Guo, Da Hu, Yan-Xi Zhao, Hui-Hui Liu, Yu-Jia Dong, Shang-Bin Sun, Xing Liu, Kang-Hong Hu, Yan-Hong Wei

**Affiliations:** ^1^ School of Material and Chemical Engineering, Hubei University of Technology, Wuhan, China; ^2^ State Key Laboratory of Structural Chemistry, Fujian Institute of Research on the Structure of Matter, CAS, Fuzhou, China; ^3^ National “111” Center for Cellular Regulation and Molecular Pharmaceutics, Key Laboratory of Fermentation Engineering (Ministry of Education), Hubei Provincial Cooperative Innovation Center of Industrial Fermentation, Hubei Key Laboratory of Industrial Microbiology, Sino-German Biomedical Center, Hubei University of Technology, Wuhan, China; ^4^ Key Laboratory of Catalysis and Energy Materials Chemistry of Ministry of Education and Hubei Key Laboratory of Catalysis and Materials Science, South-Central University for Nationalities, Wuhan, China

**Keywords:** functionalization, antiviral, hydrazide, coxsackievirus B3, polyoxometalates

## Abstract

A class of iodobenzoyldiazenido-functionalized POMs (TBA)_3_ [Mo_6_O_18_(=N=NCOAr)] (Ar = Ph-*o*-I **(1)**; Ph-*m*-I **(2)**; Ph-*p*-I **(3)**; Ph-3,4-I_2_ **(4)**; Ph-2,3,5-I_3_ **(5)** (TBA = tetrabutylammonium) were prepared *via* the refluxing reaction of *α*-octamolybdates, DCC, and corresponding hydrazides in dry acetonitrile. Their structures were determined by Fourier-transform infrared spectroscopy, ultraviolet–visible spectra, X-ray photoelectron spectroscopy, hydrogen-1 nuclear magnetic resonance, and high-resolution mass spectrometry. Research on the biological activity of title compounds shows that **L3, L5**, **3**, and **5** demonstrate potent inhibitory activity against coxsackievirus B3 and low *in vitro* cytotoxic activity against Hep-2 cell lines. The covalent linkage between the iodobenzoyldiazenido components and POMs can enhance the molecular inhibitory efficiency of iodobenzohydrazides.

## Introduction

Polyoxometalates (POMs) are a class of isolated polyanionic metal oxide clusters consisting of early transition metal (Mo, W, V, Nb, and Ta) in their high oxidation state and oxygen ligand (Hill, 1998; Pope and Muller, 2004). POMs had drawn increasing research attention not only owing to their abundant structures and various components (Cronin, 2003) but also due to their potential applications in the fields of catalysts (Wang et al., 2009), magnetism, nanoscience (Muller et al., 1998), photoelectronic material (Yamase, 1998), and medicine (Rhule et al., 1998). Especially in the field of medicine, POMs as general enzyme inhibitors (Stephan et al., 2013) were found to possess antitumor, antiviral, antibacterial (Yamase, 2005), and antidiabetic activity *in vitro* and *in vivo*. POMs have several advantages as inorganic medicine. Firstly, POMs are easy to synthesize compared with organic medicines, which usually need multiple-step synthesis (Colovic et al., 2020); secondly, POMs, as one kind of nonnucleoside drug, are less likely to occur drug resistance just like nucleoside drugs (Rhule et al., 1998). However, the tissue toxicity of POMs renders their clinical application to be a great challenge (De Bournonville et al., 2020). Moreover, the giant molecular weight of POMs usually needs a high dose in the practical applications of POMs; miniaturization of POM drugs is also very important to lessen the dose of POMs for further applications (Colovic et al., 2020). Therefore, it is necessary to improve the biological activities of POMs and/or reduce the issue toxicity of POMs if POMs medicine will move toward applications in the future. One important strategy, namely, organically modification *via* a covalent bond, was applied to modify the structure and properties of POMs.

The terminal oxo or bridging oxo atoms (O_t_) of POMs can be replaced by alkoxy or organic amine ligand to form corresponding organically derivatized POMs (Proust et al., 2012; Zhang et al., 2019). Especially for these derivatives of terminal O_t_ of POMs replaced by organic amine *via* multiple covalent bond, organoimido derivatives of POMs (Clegg et al., 1995; Strong et al., 2000; Wei et al., 2001) had received increasing research attention owing to the advantage of bond adding value (Xue et al., 2008). It can not only combine the properties of organic ligands and POMs but also possibly result in new properties due to the conjugation between organic ligands and POMs (Peng et al., 2004; Zhang et al., 2012). However, only a few efforts had been paid to the study on the derivatives of POMs modified by hydrazides (Wang et al., 2017) and phenylhydrazine (Kwen et al., 1999; Bustos et al., 2003). Hydrazides are one class of organic intermediates with various biological activities (Majumdar et al., 2014). For example, isoniazid has been used as a basic remedy for the therapy of tuberculosis during the past 70 years (Longmore, 2007). We had reported a class of benzoyldiazenido-functionalized POMs, which demonstrate enhanced antitumor activities compared with its parent POMs and corresponding ligands (Wang et al., 2017). In this research, we chose one kind of small POM platform, hexamolybdates (**POM-0**), to be modified by a series of iodobenzyol hydrazides.

Coxsackievirus B3 (CVB3), as one kind of non-enveloped and single-stranded (+) RNA virus (Rotbart, 2002), can cause many diseases, including aseptic meningitis, viral myocarditis, encephalitis, respiratory infection, hepatitis, pancreatitis, acute episodes of the hand and foot, and mouth disease of children (Bryant et al., 2004). To date, some compounds have been reported to demonstrate inhibitory activities against CVB3 (Kim et al., 2012; Wei et al., 2020). However, none of them have received final market approval owing to their low inhibitory activities and adverse effects (Fechner et al., 2011). Therefore, it is still significant to search for new compounds with high inhibitory activities and low adverse effects.

To explore organically derivatized POMs with antivirus activity, we had prepared a series of iodobenzoic hydrazide-functionalized hexamolybdates *via* multiple covalent bond of Mo=N, namely, (TBA)_3_ [Mo_6_O_18_(=N=NCOAr)] (Ar = Ph-*o*-I (**1)**; Ph-*m*-I (**2)**; Ph-*p*-I (**3)**; Ph-3,4-I_2_ (**4)**; Ph-2,3,5-I_3_ (**5)** (TBA = tetrabutylammonium), which are obtained by the refluxing reaction of corresponding hydrazides, DCC, and *α*-octamolybdates in dry acetonitrile. They were characterized by hydrogen-1 nuclear magnetic resonance (^1^H NMR), high-resolution mass spectrometry (HRMS), Fourier-transform infrared spectroscopy (FTIR), and ultraviolet–visible (UV-Vis) spectroscopy. Compounds **3** and **5** demonstrate enhanced molecular antivirus activity against CVB3 in comparison with **POM-0** and corresponding hydrazides. Herein, we reported their syntheses, characterizations, and antivirus activities against CVB3.

## RESULTS AND DISCUSSION

### Synthesis and Characterization of Title Compounds

**SCHEME 1:**
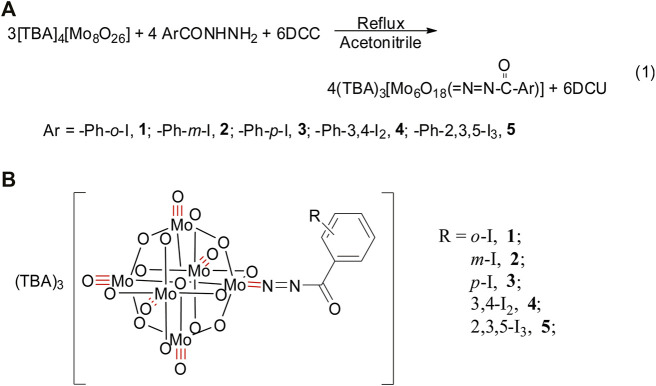
Synthetic route **(A)** and molecular structure **(B)** scheme of compounds **1**–**5**.

Iodobenzohydrazides of *o*-iodobenzohydrazide (**L1**), *m*-iodobenzohydrazide (**L2**), *p*-iodobenzohydrazide (**L3**), 3,4-diiodobenzohydrazide (**L4**), and 2,3,5-triiodobenzohydrazide (**L5**) can be conveniently prepared by the refluxing reaction of hydrazine hydrate (85%) and corresponding ester in the solvent of ethanol, which were evidenced by FTIR (Supplementary Figures S1−S5), ^1^H NMR, ^13^C NMR (Supplementary Figures S11−S15), and electrospray ionization mass spectrometry. Among them, **L4** and **L5** are prepared for the first time. As shown in Scheme 1, compounds **1**–**5** were obtained *via* the refluxing reaction of *α*-octamolybdates, corresponding iodobenzohydrazides and DCC with the molar ratio of 3:4:6 in the solvent of anhydrous acetonitrile. The resulting mixture was filtered to remove the insoluble white precipitates of DCU and afforded a black-red filtrate, which is diffused by ethyl ether. Two weeks later, lots of black or black-red crystals can be obtained, accompanied by a lot of white block crystals (they are proved to be octamolybdates by FTIR). It is pitiful that these crystals of title compounds cannot be collected by single-crystal X-ray diffraction, although we had tried to collect their crystals many times. According to our previous research (Wang et al., 2017), the molecular formula of compounds **1**–**5** are (TBA)_3_ [Mo_6_O_18_(=N=NCOAr)] (Ar = Ph-2-I (**1)**; Ph-3-I (**2)**; Ph-4-I (**3)**; Ph-3,4-I_2_ (**4)**; Ph-2,3,5-I_3_ (**5)** (TBA = tetrabutylammonium), respectively. Their structures can be viewed that one terminal O_t_ of hexamolybdates (**POM-0**) was replaced by the ligand of iodobenzoyldiazenido, **POM-0**, and the corresponding iodobenzoyldiazenido is connected *via* the Mo-N multiple covalent bond (as shown in Scheme 1). Their structures are determined and evidenced by UV-Vis, FTIR, X-ray photoelectron spectroscopy (XPS), ^1^H NMR, HRMS, and elemental analysis.

UV-Vis spectroscopy of title compounds was measured in their solution of acetonitrile (approximately 10^–5^ M) at room temperature. As shown in Supplementary Figures S16, the lowest absorption band of compounds **1**–**5** are found as broadband with the peak of 370 nm for **1**, 382 nm for **2**, 384 nm for **3**, 392 nm for **4**, and 392 nm for **5**. There is a bathochromic shift approximately 60 nm compared with that of their parent POMs of hexamolybdate, in which the lowest absorption arising from the electron transfer of O_t_ to Mo (LMCT) appeared at approximately 320 nm (Strong et al., 2000). Notably, the lowest absorption band of **1** is lower than that of other compounds; this is possible because the large steric hindrance of iodo atom in POM-**1** compels the acryl group to deviate from the phenyl ring plane and destroy their conjugation between the phenyl ring and POMs (Gougoutas and Clardy, 1970). The large bathochromic shift of the lowest absorption band in title compounds indicates the formation of the multiple bond Mo=N, in which the hydrazide ligand is connected with POMs *via* the multiple covalent bond of Mo=N, the conjugation between the organic ligand and POMs lowers the energy level of title compounds and results in the large bathochromic shift of title compounds.

FTIR spectra are further used to study the structure of title compounds. As shown in Supplementary Figures S6−S10, in the high wavenumber region of 1,350–4,000 cm^−1^, the peaks around approximately 2,960, 2,873 cm^−1^ in **1**, 2,960, 2,872 cm^−1^ in **2**, 2,960, 2,873 cm^−1^ in **3**, 2,960, 2,873 cm^−1^ in **4**, and 2,960, 2,873 cm^−1^ in **5** are ascribed as the C-H stretching vibration of methyl and methylene, verifying the presence of TBA cation. The strong peak around approximately 1,638 cm^−1^ in **1**, 1,647 cm^−1^ in **2**, 1,639 cm^−1^ in **3**, 1,637 cm^−1^ in **4**, and 1,628 cm^−1^ in **5** is attributed to the stretching vibration of C=O in compounds **1**–**5**, indicating the presence of carbonyl in those compounds (Wang et al., 2017). There is a slight redshift approximately 5–10 cm^−1^ relative to the υ(C=O) in corresponding ligands (1,638 cm^−1^ in **L1**, 1,640 cm^−1^ in **L2**, 1,649 cm^−1^ in **L3**, 1,650 cm^−1^ in **L4**, and 1,632 cm^−1^ in **L5**). The redshift of υ(C=O) in compounds **1**–**5** is mainly originating from the conjugation between the organic segment of benzoyldiazenido and POMs. In the low wavenumber region of 400–1,350 cm^−1^, the strong peak around 944 cm^−1^ in compounds **1–5** is attributed to the stretching vibration of Mo=O_t_, and their shoulder peaks around 968 cm^−1^ are associated with the stretching vibration of Mo=N. Compared with that of the parent POMs, hexamolybdates, in which the stretching vibration of Mo=O_t_ appeared at 960 cm^−1^ (Clemente-León et al., 2001), there is a redshift approximately 17 cm^−1^ for υ(Mo=O) as one consequence of one terminal O_t_ substituted by benzoyldiazenido ligand. The new-formed shoulder peak around 968 cm^−1^ can be diagnostic for the formation of Mo=N. Similarly, another peak around 799 cm^−1^ associated with the stretching vibration of Mo-Ob in hexamolybdates is also found to be split into two peaks (798 and 767 cm^−1^ for **1**, 791 and 766 cm^−1^ for **2**, 799 and 768 cm^−1^ for **3**, 792 and 769 cm^−1^ for **4**, and 792 and 769 cm^−1^ for **5**) owing to the substitution of benzoyldiazenido ligand.

XPS was performed to further confirm their composition. Full XPS spectra of compounds **1**–**5** reveal the presence of C, N, O, Mo, and I in those compounds (Supplementary Figures S11−S15). Narrow XPS spectra of compounds **1**–**5** show the characteristic Mo (3d) doublet (Mo_3d5/2_, Mo_3d3/2_) approximately 231.97, 235.18 eV for **1**, 231.97, 234.7 eV for **2**, 231.97, 235.23 eV for **3**, 231.97, 235.14 eV for **4**, and 231.81, 235.05 eV for **5**, in agreement with the literature values of molybdenum (VI) (231.3 eV, 235.8 eV; Wagner et al., 1977; Zhu et al., 2019). The doublet of I (3d) (I_3d5/2_, I_3d3/2_) is found approximately 620.71, 632.24 eV in **1**, 620.87, 632.40 eV in **2**, 620.88, 632.38 eV in **3**, 620.74, 632.33 eV in **4**, and 620.67, 632.17 eV in **5**, in accordance with the literature values of iodine (I_2_) (619.9 eV, 631.42 eV; Wagner et al., 1977). XPS results confirm the presence of molybdenum and iodine in compounds **1**–**5**, and their oxidation state is +6 and -1, respectively.

^1^H NMR spectra of POMs **1–5** (Supplementary Figures S21−S26) and corresponding ligands **L1**–**L5** (Supplementary Figures S16−S20) in *d*_6_-DMSO solution (Supplementary Table S1) show clearly resolved signals, all of which can be unambiguously assigned. Three signals (8.29, 8.01, and 7.54 ppm) of aryl hydrogen and two signals (8.24, 8.15 ppm) of aryl hydrogen are found in the ^1^H-NMR spectra of **L4** and **L5**, respectively, indicating the successful preparation of **L4** and **L5**. In the ^1^H-NMR spectra of compounds **1–5**, these signals of TBA are found approximately 3.17, 1.57, 1.32, and 0.93 ppm and further evidenced the presence of TBA. The signals of –N*H*N*H*_2_ of **L1**–**L5** disappeared in the ^1^H-NMR spectra of compounds **1–5**, but the signals of aryl hydrogen around 7–8 ppm are found in that of compounds **1–5,** which means the presence of aryl ring and the formation of Mo=N multiple bond in compounds **1–5**. As shown in Supplementary Table S1, the chemical shifts of aryl hydrogen in compounds **1**–**5** show slight upfield shifts relative to that of corresponding hydrazides. Notably, only one signal of aryl hydrogen (2H) is found in **5**, which appears at the place of 8.05 ppm; this is different from that of **L5**, which possesses two signals around 8.24 and 8.15 ppm. It can be deduced that both aryl hydrogens in **5** moved to the same chemical shifts coincidentally after the combination of **L5** with hexamolybdates *via* multiple covalent bond of Mo-N. The changing trend of chemical shifts in compounds **1**–**5** is quite different from their sisters, arylimido derivatives, in which all aryl hydrogen atoms demonstrate an obvious downfield shift compared with that of corresponding free aryl amines (Wei et al., 2001; Wang et al., 2009). POM cluster plays a role of the electron-withdrawing functional group in the arylimido derivatives. However, the POM cluster of [Mo_6_O_18_(NN)]^−3^ in compounds **1**–**5**, as an anion cluster, possessing stronger electron-donating ability compared with –NHNH_2_, can alleviate the electron deficiency of carbonyl to some extent and lower the deshielding effect of aryl ring to aryl hydrogen.

Gas chromatography coupled to HRMS was used to study compounds **1**–**5**. As shown in Supplementary Figures S27−S36, in the positive region, there are one or two molecular ionic peaks of compounds **1-4,** one molecular ionic peak found m/z = 2,092.4213 in **1**, 2,092.4216 in **2**, 2,093.4179 in **3**, and 2,218.3156 in **4** is corresponding to the molecular ion of [M + TBA]^+^ (calcd: 2,092.4286 for **1**–**3**, calcd. 2,218.3252 for **4**); another small molecular ionic peak found m/z = 1,852.1452 in **1**, 1,853.1464 in **2**, and 1,852.1452 in **3** is well matched with the molecular ion of [M + H]^+^ (calcd: 1,852.1511). In the negative mode, the strong peak found m/z = 1,607.8468 in **1**, 1,607.8475 in **2**, 1,607.8449 in **3**, and 1,733.7453 in **4** is corresponding to the molecular ion of [M - TBA]^1-^ (calcd: 1,607.8434 for **1**–**3**, calcd. 1,733.7400 for **4**). The small peak found m/z = 1,366.5709 in **1**, 1,366.5709 in **2**, 1,366.5690 in **3**, and 1,492.4680 in **4** is corresponding to the molecular ion of [M–2TBA + H]^1-^ (calcd: 1,366.5658 for **1**–**3**, calcd. 1,492.4625 for **4**). Different from that of compounds **1**–**4**, in the HRMS of compound **5,** none molecular ionic peak of [M + TBA]^+^ or [M + H]^+^ in the positive pattern except one ionic peak of [M–I + TBA]^+^ (calcd. 2,218.3252) found m/z = 2,218.3205; in the negative pattern, a very small molecular ionic peak of [M - TBA]^1-^ (calcd. 1,859.6439) found m/z = 1,859.6378. Moreover, two ionic peaks of [M - I- TBA]^1-^ (calcd. 1,732.7472) and [M–I–2TBA + H]^1-^ (calcd. 1,492.4697) found m/z = 1,732.7460 and 1,492.4689, respectively. This means that compound **5** is easy to lose one iodine atom under the ionization process.

### Biological Activity of L1–L5 and Compounds 1–5

These synthesized compounds were firstly evaluated for *in vitro* cytotoxic effects against Hep-2 (human laryngeal epithelial cancer) cell lines by the standard 3-(4,5-dimethylthiazol-2-yl)-2,5-diphenyl tetrazolium bromide assay (Mosmann, 1983; Priti et al., 2018); the 50% cell cytotoxic concentrations (CC_50_s) of compounds are summarized in Table 1. As shown in Table 1, most of the compounds showed low toxicity to the tested cell lines. It is obvious that the CC_50_s of most compounds except compound **3** are larger than that of corresponding ligands, indicating that the covalent modification can reduce the *in vitro* cytotoxic effects of hydrazides to some extent.

**TABLE 1:** Cytotoxicity and antiviral activity of synthesized compounds against CVB3.

Entry	Compds	CC_50_ ^a^ (μg/ml)	EC_50_ ^b^ (μg/ml)	SI^c^
1	L1	95.00	−^d^	−
2	L2	109.38	−	−
3	L3	188.13	52	3.62
4	L4	115.00	−	−
5	L5	125.00	13	9.62
6	0	188.13	50	3.76
7	1	161.88	−	−
8	2	166.25	−	−
9	3	183.13	46	3.98
10	4	150.00	−	−
11	5	128.13	12	10.68

a CC_50_, compound concentration required to reduce cell viability by 50%.

b EC_50_, compound concentration required to achieve 50% protection from virus-induced cytopathogenicity.

c Selective index was calculated as ratio of CC_50_ *versus* EC_50_, SI (selective index) = CC_50_/EC_50_.

d −, activity that is lower than 50% inhibition.

**FIGURE 1:**
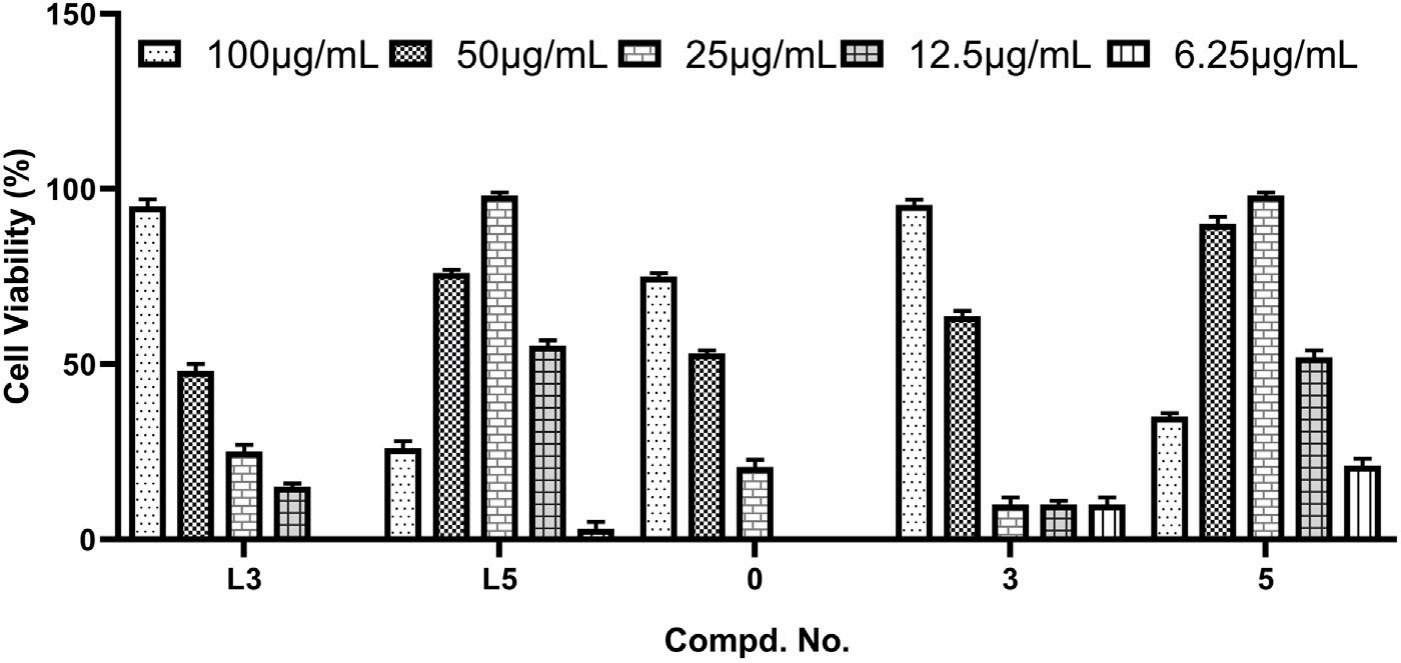
Column diagram of antiviral activities of **L3**, **L5**, **POM-0**, **3**, and **5** against CVB3.

According to the *in vitro* cytotoxic results, the antiviral activities of title compounds were further evaluated for the inhibition of virus-induced cytopathic effects. The concentration-dependent antiviral activities are shown in Figure 1. The results showed that these tested compounds displayed moderate to good inhibitory activities against CVB3. The parent POMs, hexamolybdates (**POM-0**), exhibit moderate antiviral effects. Although compounds **L3**, **L5**, **3**, and **5** with inhibition rates beyond 90% exhibited potent anti-CVB3 activities, they showed better inhibitory activity than the positive control ribavirin. Especially for **L5** and **5**, they demonstrate the lowest EC_50_s and the highest selective index in these compounds. This is possible because there are three iodine atoms in **L5** and **5**, which afford them more chances to combine with target points of CVB3s *via* halogen oxygen interaction and hydrogen bonding. Notably, the molecular inhibition efficiency of **3** and **5** surpasses that of **L3** and **L5**, considering the large molecular weight of **3** and **5**, indicating their molecular inhibition efficiency against CVB3 enhanced after the covalent linkage between the hydrazides and POMs.

To further explore the potential antiviral activities, EC_50_ values of the tested compounds were also evaluated, and ribavirin was used as a positive control. As shown in Table 1, EC_50_s of **L5** and **5** were 13 and 12 μg/ml, respectively. The selectivity index (SI) of **L5** and **5** were 9.6 and 10.7, respectively. It means that both compounds showed more potent inhibitory activity against CVB3 than ribavirin (EC_50_ = 24.7 μg/ml, SI = 8.3).

**FIGURE 2:**
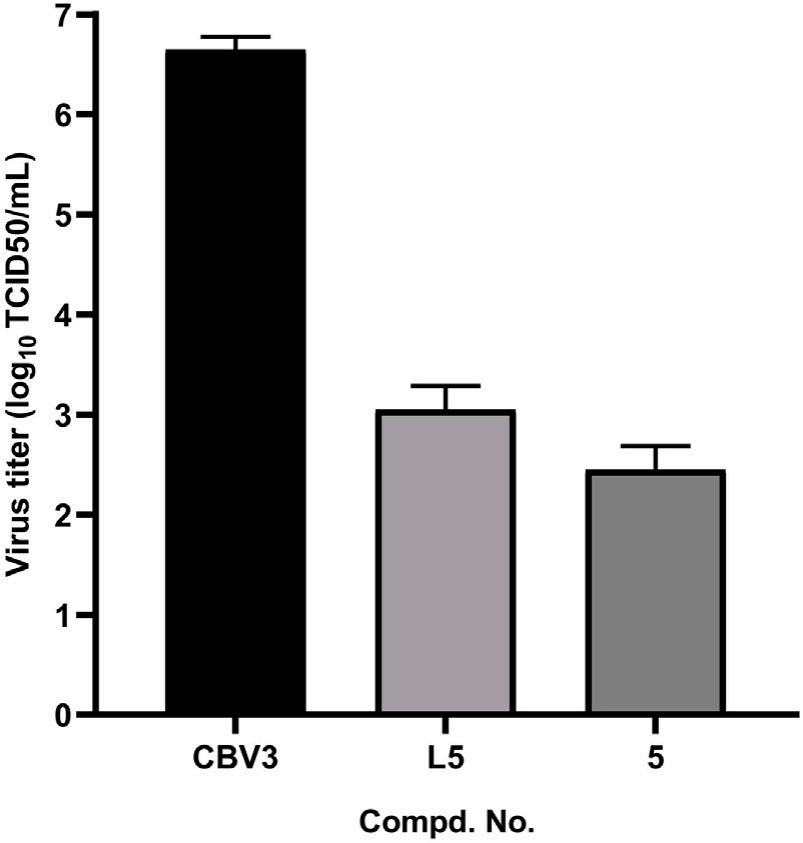
Effects of **L5** and **5** on progeny virus yields against CVB3.

To this end, the confluent monolayers Hep-2 cells in a 96-well plate were infected with 100 TCID50 of CVB3 mixed with or without the tested compounds **L5** and **5** at the concentration of 25 μg/ml. After 10 h, the culture media and cell lysates were collected after freeze–thaw cycles and then subjected to virus titration. Treatments with tested compounds resulted in efficient reductions in progeny virus titers (Figure 2), with a reduction of approximately 3.5 log for **L5** and a 4.0 log reduction for compound **5**.

## Conclusion

In conclusion, we had successfully prepared a series of iodobenzoyldiazenido-functionalized POMs (TBA)_3_ [Mo_6_O_18_(=N=NCOAr)] (Ar = Ph-*o*-I (**1)**; Ph-*m*-I (**2)**; Ph-*p*-I (**3)**; Ph-3,4-I_2_ (**4)**; Ph-3,2,3-I_3_ (**5)** (TBA = tetrabutylammonium) *via* the refluxing reaction of *α*-octamolybdates, DCC, and corresponding hydrazides (**L1**–**L5**) in dry acetonitrile. Their structures are determined and evidenced by FTIR, UV-Vis spectroscopy, XPS, ^1^H NMR, and HRMS. The biological activity test of title compounds and corresponding hydrazides shows that **L3**, **L5**, **3**, and **5** demonstrate inhibition activity against CVB3; the covalent modification of hydrazides and hexamolybdate can increase the molecular inhibition efficiency against CVB3. Among them, **L5** and **5** can reduce the progeny virus titers of CVB3 effectively.

## Data Availability

The original contributions presented in the study are included in the article/Supplementary Material, further inquiries can be directed to the corresponding authors.
