# Supplementary material for: Syntheses, Characterizations, and Inhibition Activities Against Coxsackievirus B3 of Iodobenzoic Hydrazide Functionalized Hexamolybdates
Source: Front Chem. 2022 Mar 17;10:841151. doi: 10.3389/fchem.2022.841151 (PMC8968398; doi:10.3389/fchem.2022.841151)
Supplement: Supplementary file 1 [file DataSheet1.docx]

**Syntheses, Characterizations and Inhibition Activities against CVB3 Virus of Iodobenzoic Hydrazides Functionalized Hexamolybdates**

Long-Sheng Wang^1,4*^, Chao Guo^1^, Da Hu^2^, Yan-Xi Zhao^3^, Hui-Hui Liu^2^, Yu-Jia Dong^1^, Shang-Bin Sun^1^, Xing Liu^1^, Kang-Hong Hu^2^, Yan-Hong Wei^2,*^

^1^School of Material and Chemical Engineering, Hubei University of Technology, Hubei Wuhan, 430068, P.R. China

^2^National “111” Center for Cellular Regulation and Molecular Pharmaceutics, Key Laboratory of Fermentation Engineering (Ministry of Education), Hubei Provincial Cooperative Innovation Center of Industrial Fermentation, Hubei Key Laboratory of Industrial Microbiology, Sino-German Biomedical Center, Hubei University of Technology

^3^Key Laboratory of Catalysis and Energy Materials Chemistry of Ministry of Education & Hubei Key Laboratory of Catalysis and Materials Science, South-Central University for Nationalities, Wuhan 430074, China

^4^State Key Laboratory of structural chemistry, Fujian Institute of Research on the Structure of Matter, CAS, Fujian Fuzhou, 350002, P.R. China

*** Correspondence:**Prof. Dr. Long-Sheng Wang, E-mail: [wangls@mail.hbut.edu.cn](mailto:wangls@mail.hbut.edu.cn)

Prof. Dr. Yan-Hong Wei, E-mail: [weiyanhong925@163.com](file:///E:\user\Hugong\paper\2020\GC%20hydrazide%20I\weiyanhong925@163.com)

**Experimental Details**

**Table S1 Chemical shifts of aryl hydrogens in compounds 1-5 and corresponding hydrazides.**

**Figure S1-S5 FT-IR spectra of L1-L5.**

**Figure S6-S10 FT-IR spectra of compounds 1-5.**

**Figure S11-S15 Full XPS spectra of compounds 1-5 and narrow XPS spectra for I(3d) and Mo(3d).**

**Figure S16-S20 ^1^H NMR and ^13^C NMR spectra of L1-L5.**

**Figure S21 UV-VIS spectra of POM-0 and compounds 1-5.**

**Figure S22-S26 ^1^H NMR spectra of compounds 1-5.**

**Figure S27-S36 GC-HR MS spectra of compounds 1-5**.

**Table S2 Summary of GC-HR MS results of compound 1.**

**Table S3 Summary of GC-HR MS results of compound 2.**

**Table S4 Summary of GC-HR MS results of compound 3.**

**Table S5 Summary of GC-HR MS results of compound 4.**

**Table S6 Summary of GC-HR MS results of compound 5.**

**Experimental Details**

[Bu_4_N]_4_[*α*-Mo_8_O_26_] was synthesized according to literature procedure([Hur *et al.*, 1990](#_ENREF_2), [Wang *et al.*, 2011](#_ENREF_6))**,** confirmed by IR spectroscopy. Other chemical reagents were of analytical grade obtained commercially from Aladdin Reagent Co., Ltd, Ji’nan Henghua Reagent Co., Ltd or Sinopharm Chemical Reagent Co., Ltd, which were used as received without further purifications except as otherwise noted. Acetonitrile were dried using standard drying procedure (refluxing with CaH_2_) and distilled in an inert atmosphere prior to use([Armarego *et al.*, 2003](#_ENREF_1)). All reactions were carried out using ordinary device and operations. Accurate mass spectra (MS) were referenced against leucine enkephalin (555.6 g⋅mol^-1^) and reported within 5ppm for electrospray mass spectra. Nuclear magnetic resonance (NMR) spectra were recorded in deuterated DMSO or chloroform with a 400 MHz spectrometer at the following frequencies: 400.13 MHz for ^1^H and 100.6 MHz for ^13^C. The signals for ^1^H and ^13^C NMR spectra were referenced to TMS at *δ* 0.0 ppm, and coupling constants were recorded in hertz (Hz) to two decimal places. The samples for CHN analysis were dried in vacuum for 24 hours at 50^o^C to remove crystalized solvent. Elemental analyses were performed on Elementar Vario Micro Cube. X-ray photoelectron spectroscopy (XPS) was performed on PHI-5000 Versaprobe III (ULVAC-PHI, Japan) using monochromatic Al Kα as the excitation source. Pass energy of 25 eV was used in the XPS measurement. The base pressure of the analysis chamber was less than 6.7 × 10^-8^ Pa. All spectra were calibrated using the binding energy of C1s (285.0 eV) as a reference. FT-IR spectra were recorded on Perkin Elmer Spectrum 100 FTIR Spectrometer. UV spectra were conducted on Shimadzu UV Probe 2450.

**1 Synthesis of Ethyl Iodobenzoate**

**General Synthetic Procedure:** To a round-bottom flask with 20.0 ml absolute ethanol, iodobenzoic acid (5.0 mmol, 1.24 g for *o*-iodobenzoic acid, *m*-iodobenzoic acid, *p*-iodobenzoic acid, 1.87g for 3,4-diiodobenzoic acid, 2.49g for 2,3,5-triiodobenzoic acid) were added to form a mixture. Then 3.0 ml sulfuric acid (98%) was added slowly. The mixture was refluxed and stirred for 12 hours. The solid disappeared gradually to form a colorless solution. The reaction was monitored by TLC until the disappearance of *o*-iodobenzoic acid. The obtained solution was cooled to room temperature and reduced to about 10.0 mL under vacuum, whose PH are adjusted to 8 ~ 9 using saturated solution of Na_2_CO_3_ and then extracted by 10ml × 4 ethyl acetate, the combined organic phase were dried using MgSO_4_ and desolventized under vacuum to give a light yellow oil.

**1.1 Ethyl *o*-iodobenzoate**

Light yellow oil. Weight 0.925g; yield: 69%.

**1.2 Ethyl *m*-iodobenzoate**

Light yellow oil. Weight 0.952g; yield: 69%.

**1.3** **Ethyl *p*-iodobenzoate**

Light yellow oil. Weight 1.035 g; yield: 75%.

**1.4** **Ethyl** **3,4-Diiodobenzoate**

Light yellow solid. Weight 1.445g; yield: 69%.

**1.5** **Ethyl** **2,3,5-Triiodobenzoate**

White solid, weight 1.825g, Yield: 67%.

2. **Ligand Syntheses**

**General Synthetic Procedure:** Ethyl benzoate (0.014mol, 3.864g for ethyl *o*-iodobenzoate, ethyl *m*-iodobenzoate, ethyl *p*-iodobenzoate, 5.62g for ethyl 3,4-diiodobenzoate; 3.69g for ethyl 2,3,5-triiodobenzote(0.007mol)) was dissolved in 10.0 mL absolute ethanol to form a colorless solution. Then 7.0 ml hydrate hydrazine (85%) was added to the solution, which was stirred and heated in 70^o^C for 8 hours. Resulting solution was reduced to about 10.0 mL in vacuum and stored in the refrigerator for one day to give lots of needle-like colorless crystals, which are separated by suction filtration, and washed with cold water, ethyl ether, dried in the vacuum apparatus.

**2.1 *o*-iodobenzohydrazide (L1)**

Weight, 3.118g; yield, 85%. CHN analysis for C_7_H_7_IN_2_O, calcd. (%): C, 32.08; H, 2.69; N, 28.01; Found: C, 32.19; H, 2.75; N, 28.13. FT-IR (KBr pellet, cm^-1^): 3299(vs), 3036(w), 2830(w), 1642(vs), 1625(s), 1583(m), 1518(s), 1461(m), 1422(m), 1331(s), 1264(m), 1187(w), 1159(w), 1129(m), 1108(m), 1042(m), 1014(s), 964(s), 950(s), 888(w), 875(w), 783(m), 749(vs), 715(m), 692(m), 638(s), 444(s). ^1^H NMR (400 MHz, DMSO) δ 9.52 (s, 1H, -N*H*NH_2_), 7.88 (dd, *J* = 7.8, 1.1 Hz, 1H), 7.43 (td, *J* = 7.5, 1.2 Hz, 1H), 7.28 (dd, *J* = 7.6, 1.7 Hz, 1H), 7.17 (td, *J* = 7.7, 1.0 Hz, 1H), 4.47 (s, 2H, -NHN*H*_2_) ppm; ^13^C NMR (100 MHz, DMSO) δ 168.63, 142.12, 139.60, 131.36, 128.84, 128.40, 94.60 ppm; ESI-MS (-p): 260.97, calcd. for [L - H]^-^ 261.03.

**2.2 *m*-iodobenzohydrazide (L2)**

Weight, 3.082g; yield, 84%. CHN analysis for C_7_H_7_IN_2_O, calcd. (%): C, 32.08; H, 2.69; N, 28.01; Found: C, 31.96; H, 2.78; N, 27.96. FT-IR (KBr pellet, cm^-1^): 3311(s), 3190(s), 3043(s), 1623(vs), 1589(s), 1557(s), 1467(m), 1401(m), 1345(s), 1288(m), 1269(m), 1171(w), 1111(m), 1061(m), 994(s), 970(w, sh), 912(w), 897(w), 803(m), 708(s), 646(m), 619 (w), 500(w), 479(m). ^1^H NMR (400 MHz, DMSO) δ 9.86 (s, 1H, -N*H*NH_2_), 8.17 (s, 1H), 7.89 - 7.83 (dd, *J* = 8.0 Hz, 2H), 7.27 (t, *J* = 7.8 Hz, H), 4.53 (s, 2H, -NHN*H*_2_) ppm; ^13^C NMR (100 MHz, DMSO) δ 164.71, 142.12, 139.60, 131.36, 128.40, 94.60 ppm; ESI-MS: 261.24, calcd. for [L - H]^-^ 261.04; 247.21, calcd. for [L – NH_2_]^-^ 247.03.

**2.3**  ***p*-iodobenzohydrazide (L3)**

Weight, 3.192g; yield, 87%. CHN analysis for C_7_H_7_IN_2_O, calcd. (%): C, 32.08; H, 2.69; N, 28.01; Found: C, 32.15; H, 2.63; N, 27.72. FT-IR (KBr pellet, cm^-1^): 3296(s), 3187(s), 1923(w), 1650(s, sh), 1631(vs), 1584(s), 1557(m, sh), 1530(s), 1473(s), 1388(m), 1328(s), 1299(m), 1271(m), 1186(w, d), 1168(w, d), 1112(s), 1098(s), 1057(s), 1004(s), 961(s), 876(s), 844(s), 760(s), 705(s), 644(s), 624(s), 497(s), 458(s). ^1^H NMR (400 MHz, DMSO) δ 9.86 (s, 1H, -N*H*NH_2_), 7.84 (td, *J* = 7.2, 1.6 Hz, 2H), 7.61 (td, *J* = 7.6, 2.0 Hz, 2H), 4.55 (s, 2H, -NHN*H*_2_) ppm; ^13^C NMR (100 MHz, DMSO) δ 165.60, 137.65, 133.16, 129.38, 99.10 ppm; ESI-MS (-p): 260.96, calcd. for [L - H]^-^ 261.04.

**2.4**  **3,4-Diiodobenzohydrazide (L4)**

Weight, 4.561g; yield, 84%. CHN analysis for C_7_H_6_I_2_N_2_O, calcd. (%): C, 21.67; H, 1.56; N, 7.22; Found: C, 21.72; H, 1.64; N, 7.15. FT-IR (KBr pellet, cm^-1^): 3303(s), 3053(w), 1650(vs), 1578(m), 1528(s), 1447(m), 1321(m), 1247(s), 1114(m), 1087(w), 1003(w), 970(m), 908(w), 826(m), 748(m, d), 725(m, d), 692(w), 640(m), 491(w). ^1^H NMR (400 MHz, DMSO) δ 9.92 (s, 1H, -N*H*NH_2_), 8.29 (dd, *J* = 2.0 Hz, 1H), 8.01 (d, *J* = 8.2 Hz, 1H), 7.54 (d, *J* = 8.2, 2.1 Hz, 1H), 4.57 (s, 2H, -NHN*H*_2_) ppm; ^13^C NMR (100 MHz, DMSO) δ 164.21, 139.62, 137.70, 134.63, 128.21, 112.93, 109.32 ppm; ESI-MS (-p): 386.94, calcd. for [L - H]^-^ 386.95; 372.89, calcd. for [L – NH]^-^ 372.92.

**2.5** **2,3,5-Triiodobenzohydrazide (L5)**

Weight, 3.2g; yield, 89%. CHN analysis for C_7_H_5_I_5_N_2_O, calcd. (%): C, 16.36; H, 0.98; N, 5.45; Found: C, 16.44; H, 1.07; N, 5.53. FT-IR (KBr pellet, cm^-1^): 3299(s), 3195(m), 3062(m), 1631(vs), 1575(m, sh), 1541(s), 1518(vs), 1403(m), 1326(s), 1289(m), 1256(w), 1184(w), 1111(m), 993(m), 976(m), 907(m), 868(s), 771(m), 723(m, sh), 708(s), 670(s), 629(m), 526(w), 444(w). ^1^H NMR (400 MHz, DMSO) δ 9.95(s, 1H), 8.24 (s, 1H), 8.15 (s, 1H), 4.61 (s, 2H) ppm; ^13^C NMR (100 MHz, DMSO) δ 163.25, 146.98, 137.05, 135.37, 96.57 ppm; ESI-MS (-p): 386.95, calcd. for [L – I]^-^ 387.95; 514.54, calcd. for [L – H]^-^ 513.84.

**3. Synthesis of Compounds 1-5**

**General Synthetic Procedure:** [Bu_4_N]_4_[*α*-Mo_8_O_26_] (2.153g, 1.00mmol) and DCC (0.4659g, 2.26mmol) were added to 20ml anhydrous acetonitrile and refluxed for 40min. Then iodobenzohydrazide (1.33mmol, 0.348g for **L1**, **L2**, **L3**, 0.514g for **L4**, 0.682g for **L5**) was added to the solution, which are refluxed for another twelve hours and cooled to room temperature. Black red filtrate were obtained by filtration to remove those white precipitates and diffused by ethyl ether. Two week later, lots of black bar solid yields was obtained and washed by ethyl ether.

**3.1 [Bu_4_N]_3_[Mo_6_O_18_(=N=NCOC_6_H_4_-*o*-I)] (1)**

Black strip crystals, weight, 0.274g; yield, 14%. CHN analysis for C_55_H_112_IMo_6_N_5_O_19_, calcd. (%): C, 35.71; H, 6.10; N, 3.39; Found: C, 36.60; H, 5.97; N, 3.53. FT-IR (KBr pellet, cm^-1^) 3415(vs, br), 2961(m), 2873(m), 2387(m), 1638(m), 1481(w), 1384(vs), 1297(w), 1261(s), 1151(s), 1089(s), 1006(s), 968(s), 943(s), 924(m), 912(m), 865(s), 839(m), 798(s,d), 767(m), 663(s), 583(m), 545(s), 464(m). ^1^H NMR (400 MHz, DMSO) δ 7.80 (s, 1H), 7.43 (s, 1H), 7.30 (s, 1H), 7.00 (s, 1H), 3.17 (t, 24H), 1.57 (m, 24H), 1.32 (q, 24H), 0.93 (t, 36H).

**3.2 [Bu_4_N]_3_[Mo_6_O_18_(=N=NCOC_6_H_4_-*m*-I)] (2)**

Black strip crystals, weight, 0.313g; yield, 16%. CHN analysis for C_55_H_112_IMo_6_N_5_O_19_, calcd. (%): C, 35.71; H, 6.10; N, 3.39; Found: C, 35.74; H, 5.93; N, 3.47. FT-IR (KBr pellet, cm^-1^): 3441(vs, br), 2960(m), 2873(m), 2387(m), 1647(m), 1589(w), 1544(w), 1482(w), 1384(s), 1295(w), 1260(m), 1166(s, sh), 1090(s), 1006(w), 968(s), 944(s), 867(s), 839(m), 791(m), 766(m), 582(s), 543(s), 468(m). ^1^H NMR (400 MHz, DMSO) δ 8.07 (s, 1H), 7.75 (d, 1H), 7.70 (d, 1H), 7.13 (m, 1H), 3.17 (t, 24H), 1.57 (m, 24H), 1.31 (q, J = 16.4, 9.1 Hz, 24H), 0.93 (t, J = 13.7, 7.1 Hz, 36H).

**3.3 [Bu_4_N]_3_[Mo_6_O_18_(=N=NCOC_6_H_4_-*p*-I)] (3)**

Black strip crystals, weight, 0.390g; yield, 20%. CHN analysis for C_55_H_112_IMo_6_N_5_O_19_, calcd. (%): C, 35.71; H, 6.10; N, 3.39; Found: C, 35.83; H, 5.99; N, 3.45. FT-IR (KBr pellet, cm^-1^): 3416(vs, br), 2961(m), 2873(m), 2387(s), 2169(w), 1639(s), 1481(m), 1384(s), 1296(m), 1260(m), 1099(s), 984(s), 968(m), 944(m), 866(w), 799(m), 768(sh), 663(w), 583(m), 546(s), 464(m). ^1^H NMR (400 MHz, DMSO): δ 7.68 (s, 2H), 7.53 (d 2H), 3.26 – 3.10 (m, 24H), 1.57 (s, 24H), 1.31 (d, 24H), 0.93 (t, 36H).

**3.4 [Bu_4_N]_3_[Mo_6_O_18_(=N=NCOC_6_H_3_-3,4-I_2_)] (4)**

Black strip crystals, weight, 0.318g; yield, 15%. CHN analysis for C_55_H_111_I_2_Mo_6_N_5_O_19_, calcd. (%): C, 33.43; H, 5.66; N, 3.54; Found: C, 33.54; H, 5.75; N, 3.67. FT-IR (KBr pellet, cm^-1^): 3447(s,br), 2960(s), 2873(s), 1628(w), 1586(m), 1535(m), 1482(s), 1382(m), 1350(m), 1251(vs), 1235(s, sh), 1151(w), 1106(w), 1092(s), 968(s, sh), 943(vs), 885(m), 792(vs, d), 769(vs, d), 705(w), 651(w), 617(w), 464(w). ^1^H NMR (400 MHz, DMSO) δ 8.21 (s, 1H), 7.85 (d, J = 7.3 Hz, 1H), 7.46 (d, J = 1.9 Hz, 1H), 3.13 (t, 24H), 1.56 (t, 24H), 1.29 (t, 24H), 0.93 (t, 36H).

**3.5 [Bu_4_N]_3_[Mo_6_O_18_(=N=NCOC_6_H_2_-2,3,5-I_3_)] (5)**

Black strip crystals, weight, 0.275g; yield, 12%. CHN analysis for C_55_H_110_I_3_Mo_6_N_5_O_19_, calcd. (%): C, 31.42; H, 5.27; N, 3.33; Found: C, 31.57; H, 5.21; N, 3.35. FT-IR (KBr pellet, cm^-1^): 3447(s, br), 2960(s), 2873(m), 1628(w), 1586(m), 1535(m), 1482(s), 1382(m, d), 1350(m, d), 1252(s), 1235(s, sh), 1151(w), 1106(w), 968(m, sh), 943(vs), 885(w), 792(vs, d), 769(vs, d), 705(w), 651(w), 618(w), 464(m). ^1^H NMR (400 MHz, DMSO) δ 8.14 (s, 1H), 8.04 (s, 1H), 3.18 (s, 24H), 1.57 (s, 24H), 1.32 (s, 24H), 0.94 (s, 36H).

**4. Biological evaluation**

**4.1 Cells, virus and tested compounds**

Human laryngealcarcinoma cells (Hep-2) (purchased from China Center for type Culture Collection, CCTCC) were maintained in DMEM (Gibco) supplemented with 10% fetal bovine serum (FBS; Gibco), 100U/mL of penicillin and streptomycin, and 2 mM L-glutamine. Live CVB3 strain were kindly provided by Prof. YingZhu (State Key Laboratory of Virology, College of Life Sciences, Wuhan University, China) and propagated in the Hep-2 cells. Viral titers were determined using the standard method of median tissue culture infective dose (TCID50) ([Reed *et al.*, 1938](#_ENREF_5)). Ribavirin was used as positive controls, was purchased from Sigma Chemical Co. Stock solutions of drugs were prepared in dimethyl sulfoxide (DMSO) at a final concentration of 0.1% and diluted with maintenance medium (MM) consisting of DMEM with 2% fetal bovine serum.

**4.2 Cytotoxicity assays**

The cytotoxicities of the synthesized compounds were evaluated in human laryngeal epithelial cancer (Hep-2) cells with the 3-(4,5-dimethylthiazol-2-yl)-2,5-diphenyl tetrazolium bromide (MTT) assay ([Mosmann, 1983](#_ENREF_3), [Priti *et al.*, 2018](#_ENREF_4)). Briefly, the cells were seeded into a 96-well plate. After 24 h, the medium was replaced with fresh medium (2% fetal calf serum [FCS]) and a two-fold serial dilution of the compounds. After 48 h of exposure, the medium was removed and MTT was added and incubated for 4h at 37 ^o^C. DMSO (50ml/well) was added to dissolve the MTT formazan and the optical density of the cells was measured at 492nm (OD 492) with a microplate reader (Thermo Scientific, MK3). The 50% cell cytotoxic concentration (CC50) of compounds was calculated using statistical package for the social sciences (SPSS) software.

**4.3 Antiviral assays & selectivity index**

Antiviral activity of title compounds against CVB3 was determined by measuring its inhibition of virus-induced cytopathic effects (CPEs) in acutely infected Hep-2 cells, respectively. Generally, confluent cell monolayer since 96-well dishes were infected with 100TCID50 of CVB3 for 1.5h at 37 ◦C. Inocula were aspirated and the cells then were incubated with various concentrations of compounds at 37 ◦C, 5% CO_2_, for 48 h. CPEs were observed microscopically and the viability of the cells determined using MTT assays. The concentrations of test compounds required to achieve 50% protection from virus-induced cytopathogenicity (EC50) were determined. The selectivity index (SI) was calculated as the ratio of CC50/EC50. Each experiment was performed in triplicate and at least three independent experiments.

**4. 4 Progeny virus titration**

Viral suspensions, serially diluted tenfold with dulbecco’s modified Eagle medium (DMEM) containing 2% fetal bovine serum (FBS), were used to inoculate RD or Hep-2 cells in a 96-well plate. After 1.5 h incubation at 37 ^o^C in 5% CO_2_, unbound virus was washed out and DMEM maintenance medium (MM) supplemented with 2% FBS added to the cells. After 2 days, the infected cells were monitored for CPEs. Virus titers were calculated by the Reed–Muench method([Reed *et al.*, 1938](#_ENREF_5)).

Table S1 Chemical shift of aryl hydrogen in compounds 1-5 and corresponding hydrazines.

|  | FG =  [Mo_6_O_18_(=N=N−)]^3-^ | FG =  NH_2_NH− |  |
| --- | --- | --- | --- |
|  | a 7.80  b 7.43  c 7.30  d 7.00 | a 7.88  b 7.48  c 7.28  d 7.17 |  |
|  | a, b 7.75  b 7.70  c 7.13 | a, b 7.97  b 7.27  c 7.17 |  |
|  | a 7.68  b 7.53 | a 7.84  b 7.61 |  |
|  | a 8.21  b 7.85  c 7.46 | a 8.29  b 8.01  c 7.54 |  |
|  | a,b 8.05 | a 8.24  b 8.15 |  |


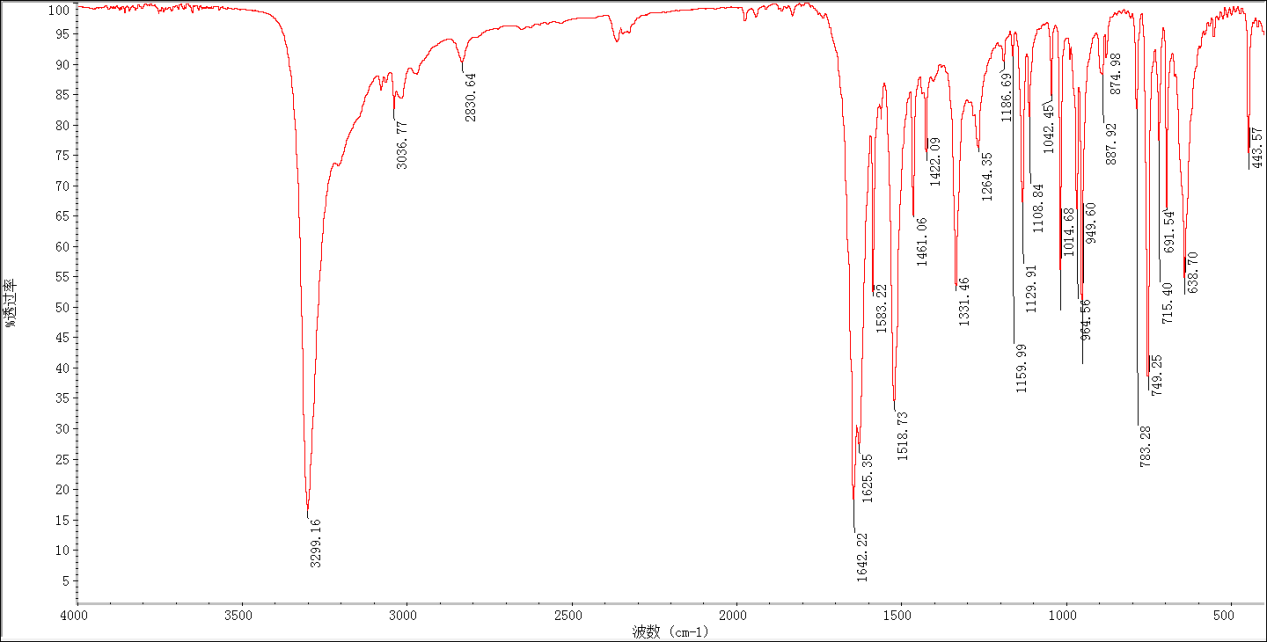


Figure S1 FT-IR spectrum of **L1**


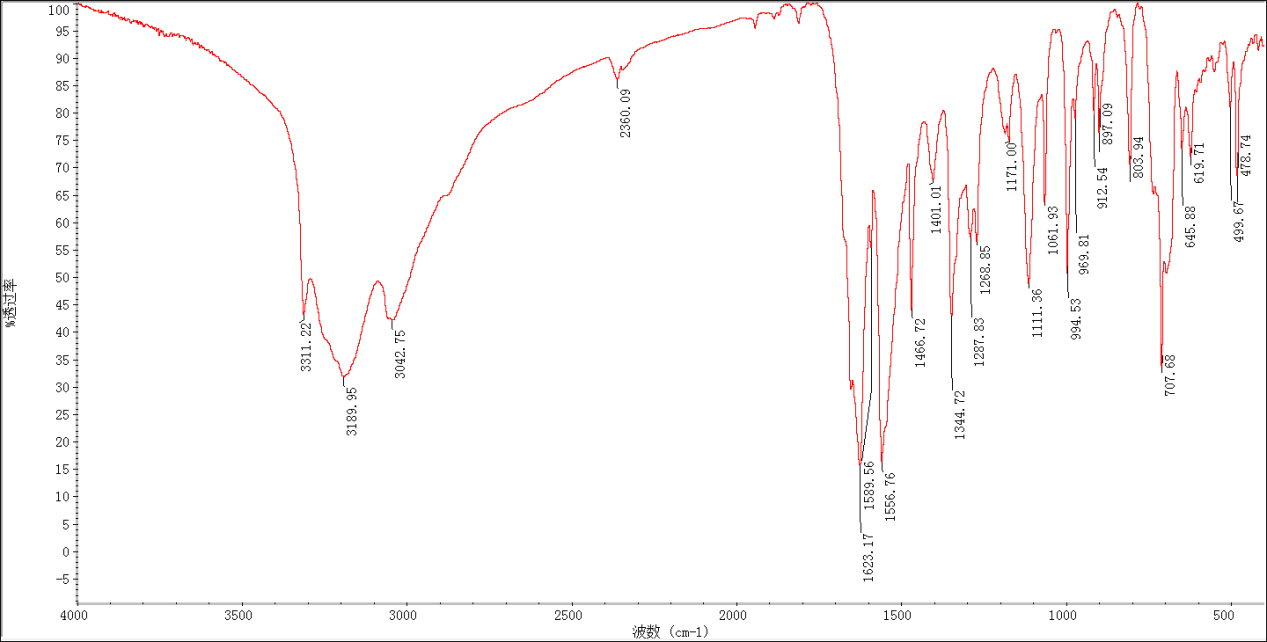


Figure S2 FT-IR spectrum of **L2**


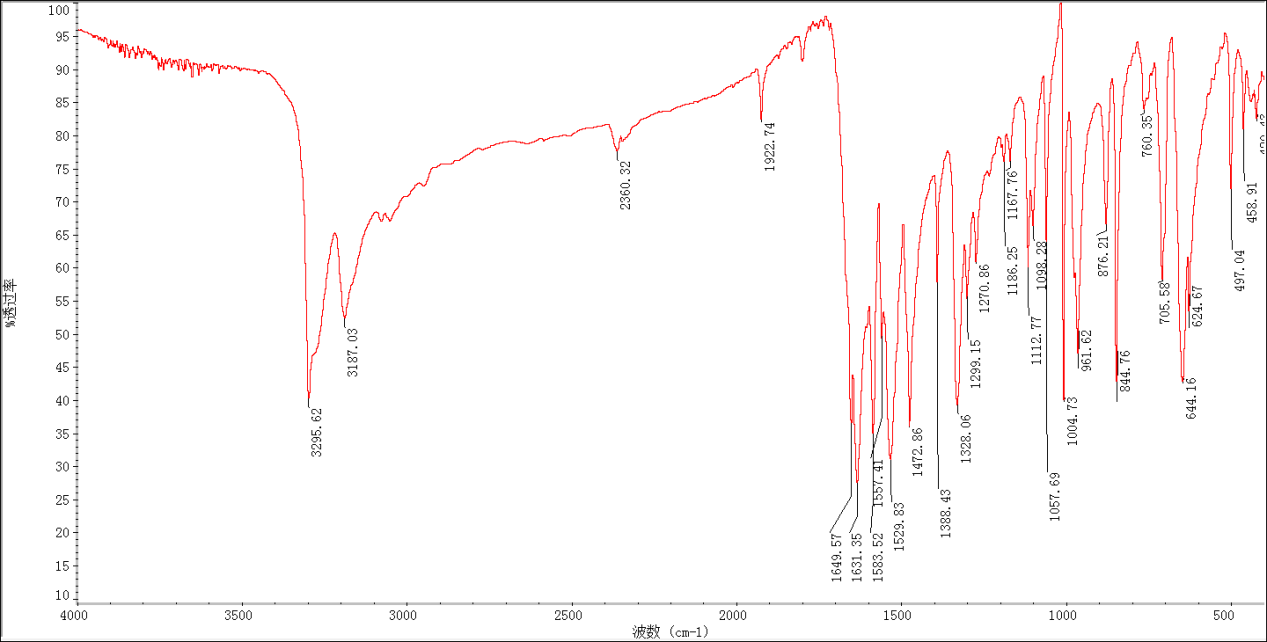


Figure S3 FT-IR spectrum of **L3**


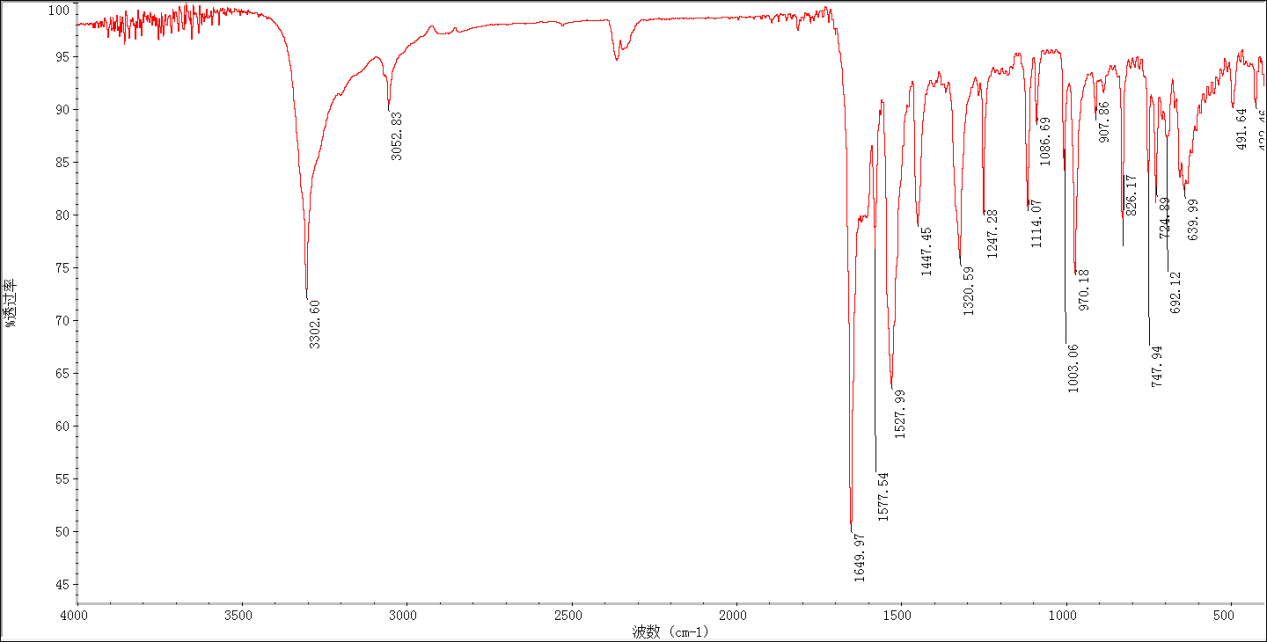


Figure S4 FT-IR spectrum of **L4**


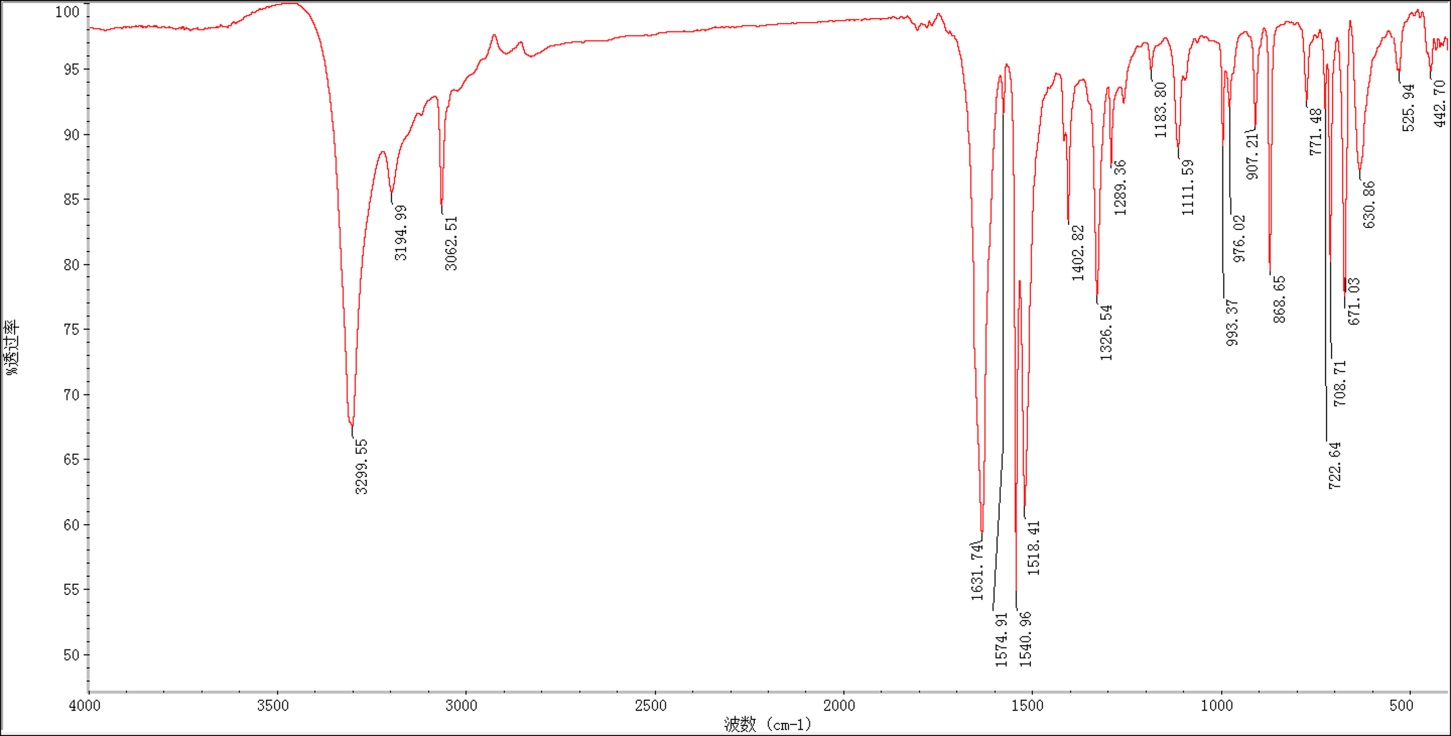


Figure S5 FT-IR spectrum of **L5**

**
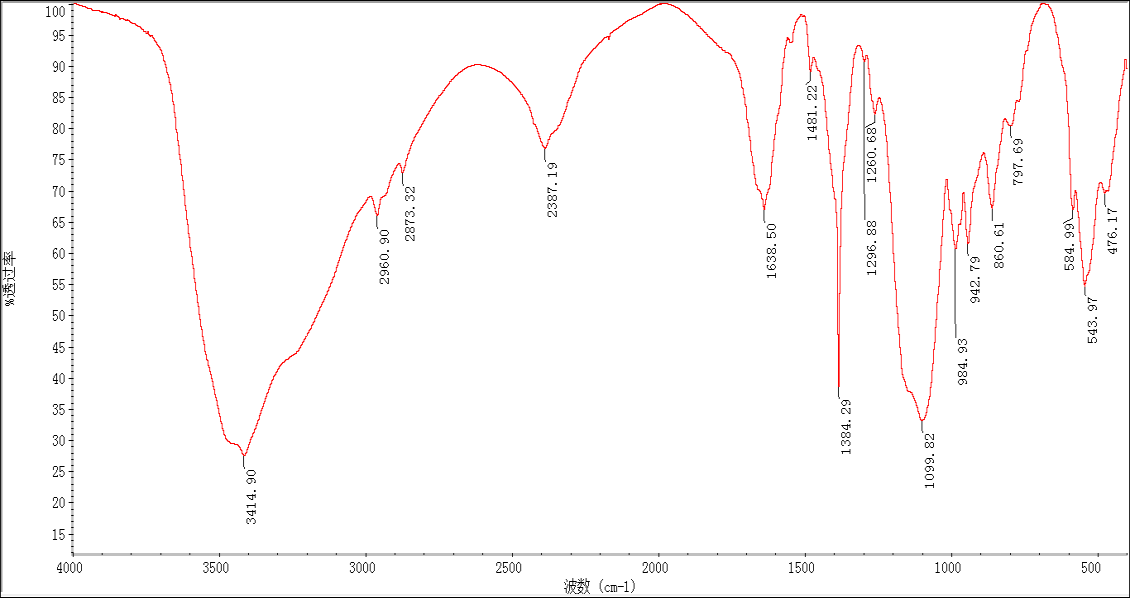
**

Figure S6. FT-IR spectrum of [Bu_4_N]_3_[Mo_6_O_18_(=N=NCOC_6_H_4_-2-I)] **(1)**


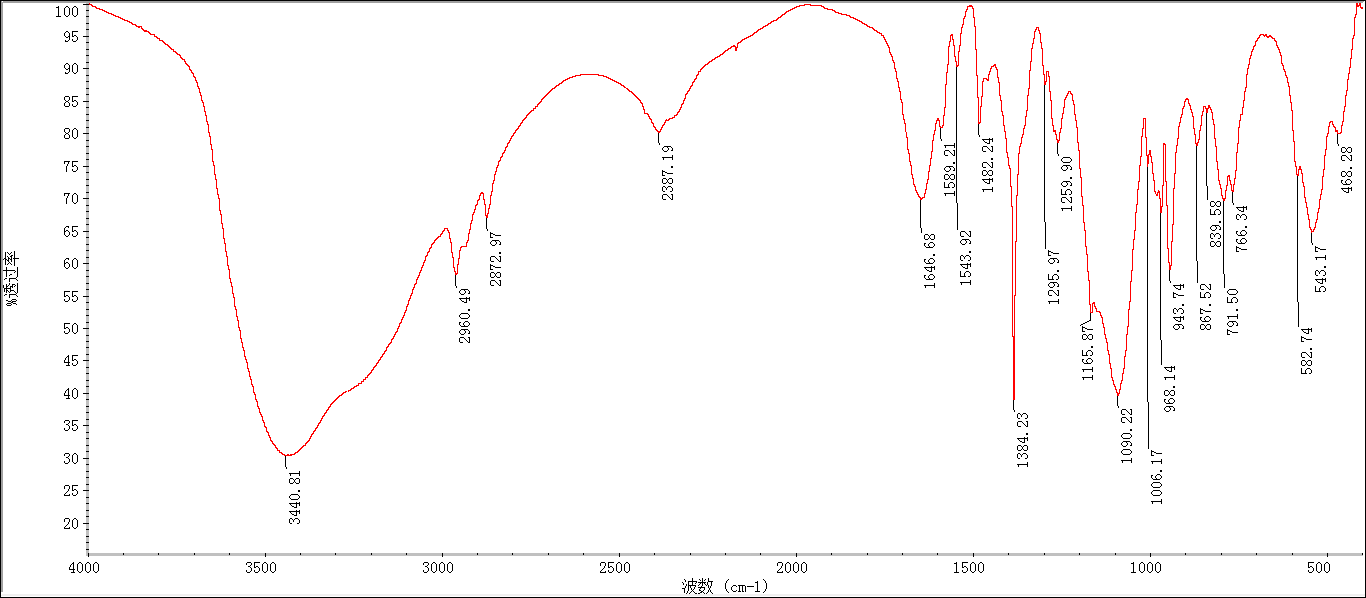


Figure S7. FT-IR spectrum of [Bu_4_N]_3_[Mo_6_O_18_(=N=NCOC_6_H_4_-3-I)] (**2**)


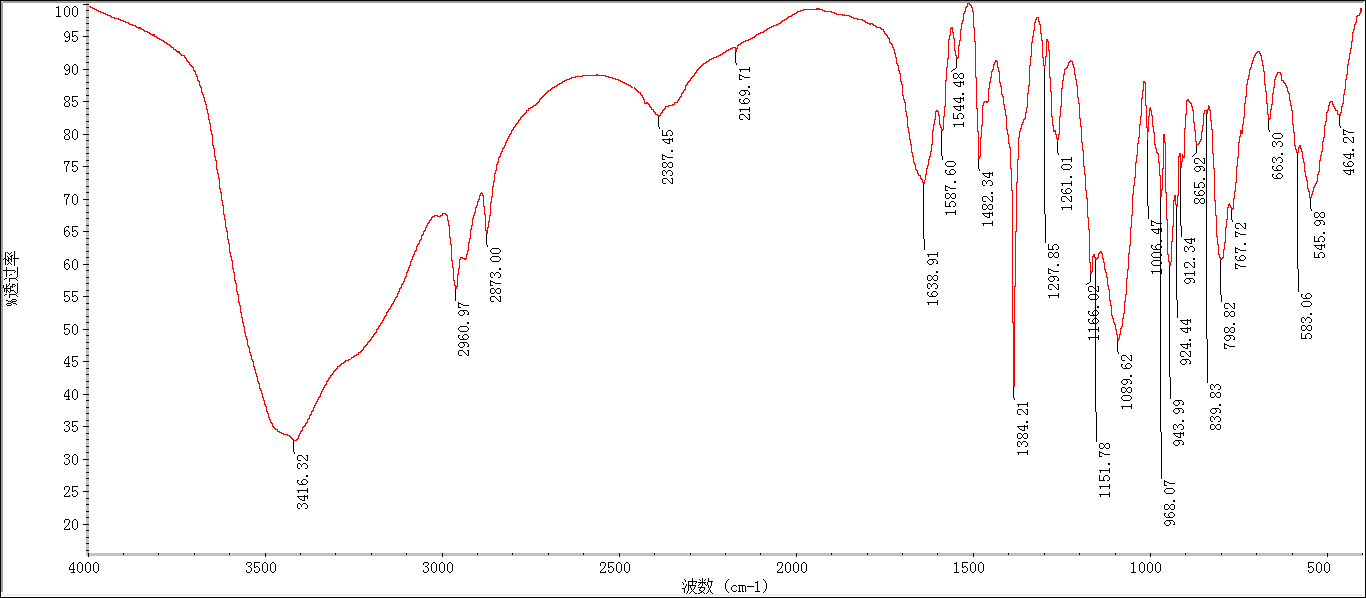


Figure S8. FT-IR spectrum of [Bu_4_N]_3_[Mo_6_O_18_(=N=NCOC_6_H_4_-4-I)] (**3**)

**
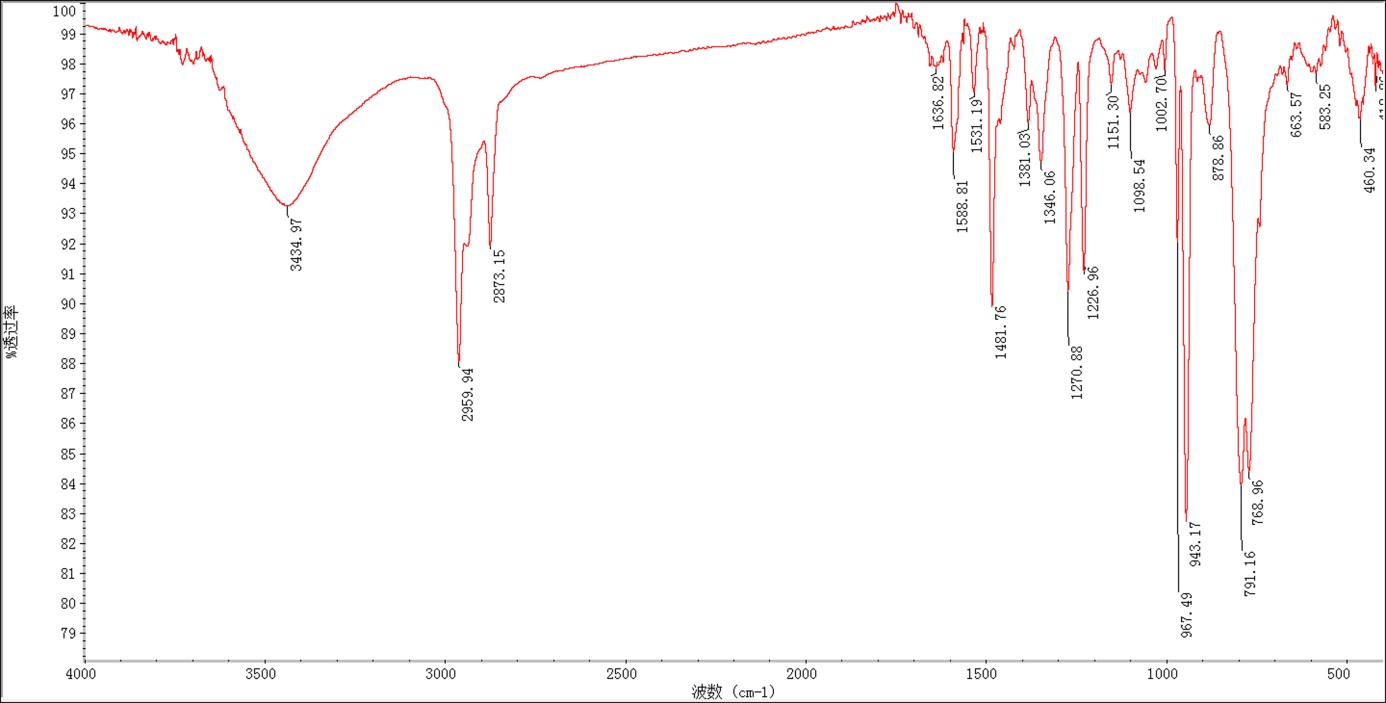
**

Figure S9. FT-IR spectrum of [Bu_4_N]_3_[Mo_6_O_18_(=N=NCOC_6_H_3_-3,4-I_2_)] (**4**)

**
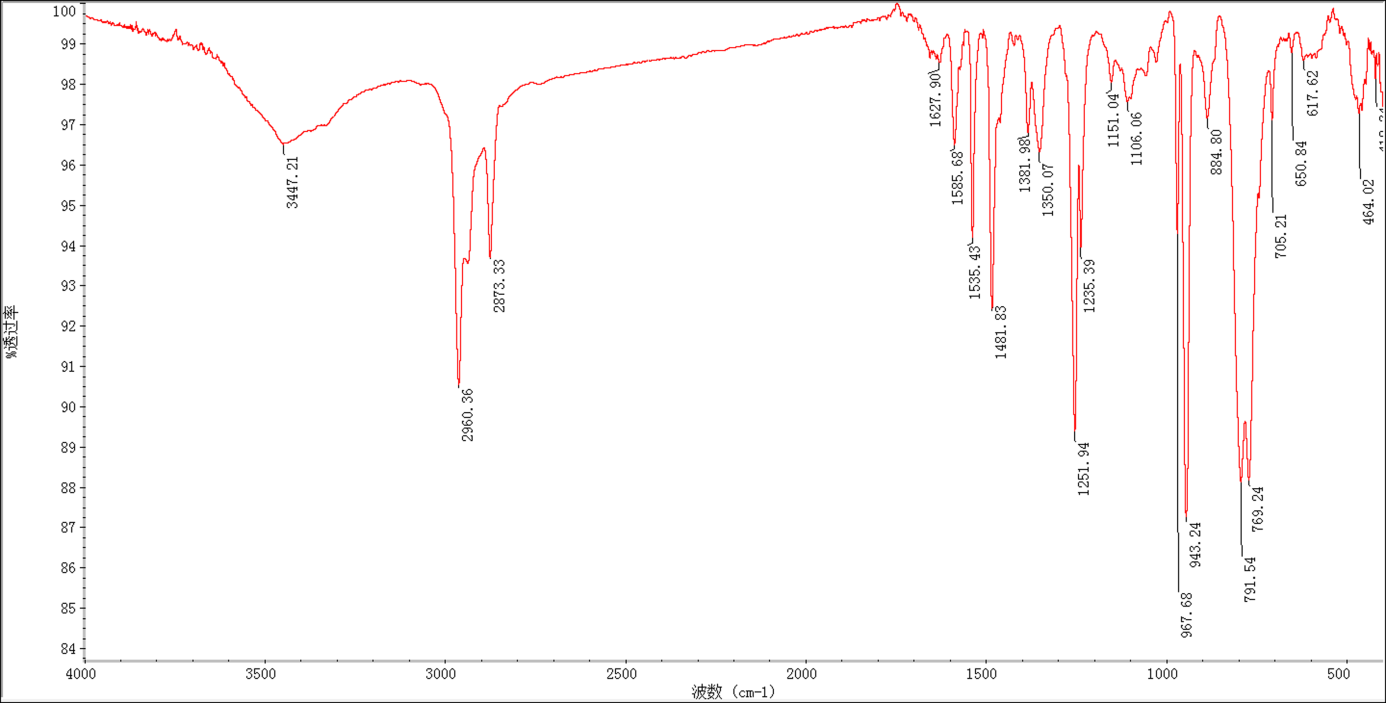
**

Figure S10. FT-IR spectrum of [Bu_4_N]_3_[Mo_6_O_18_(=N=NCOC_6_H_2_-2,3,5-I_3_)] (**5**)


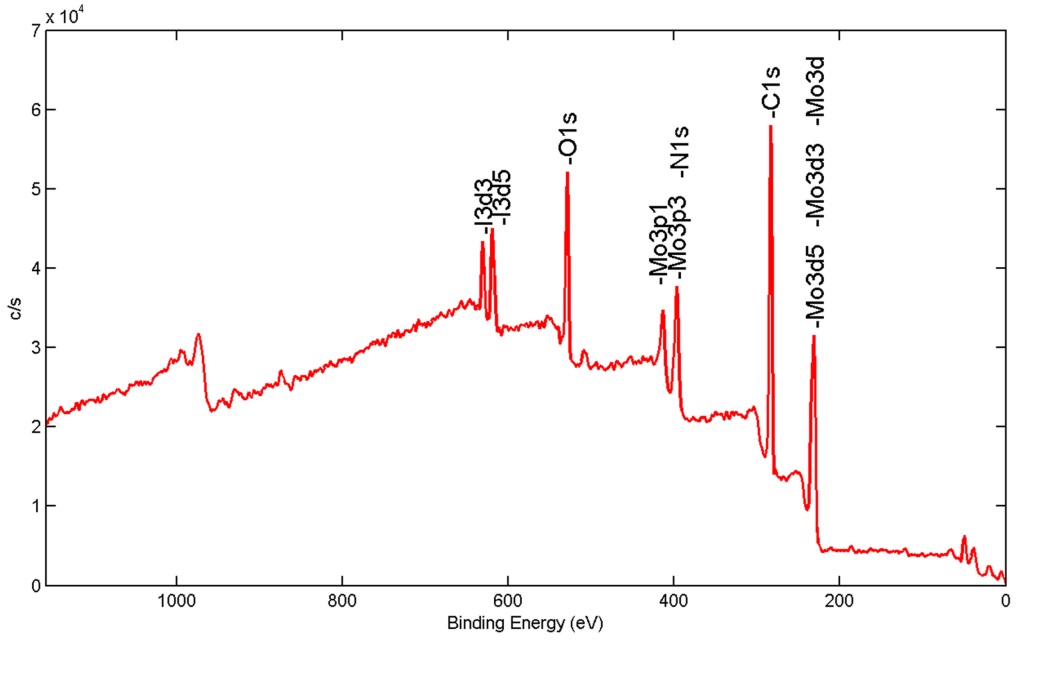


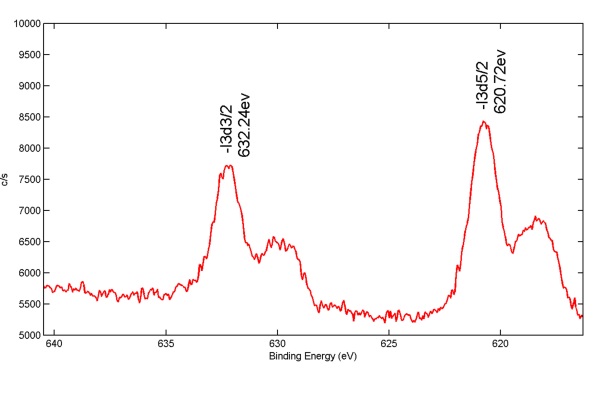

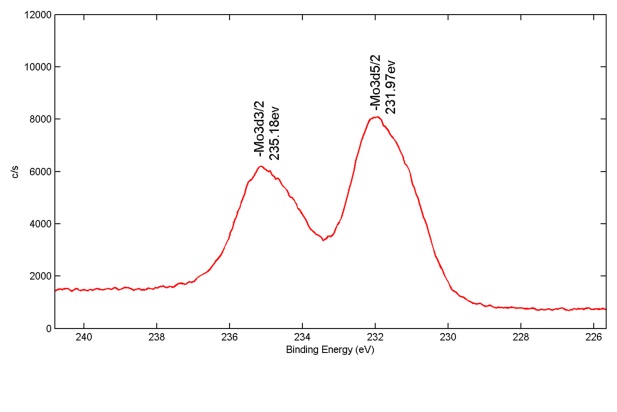


Figure 11 Full XPS spectra of compound **1** and narrow XPS spectra for I(3d) and Mo(3d).


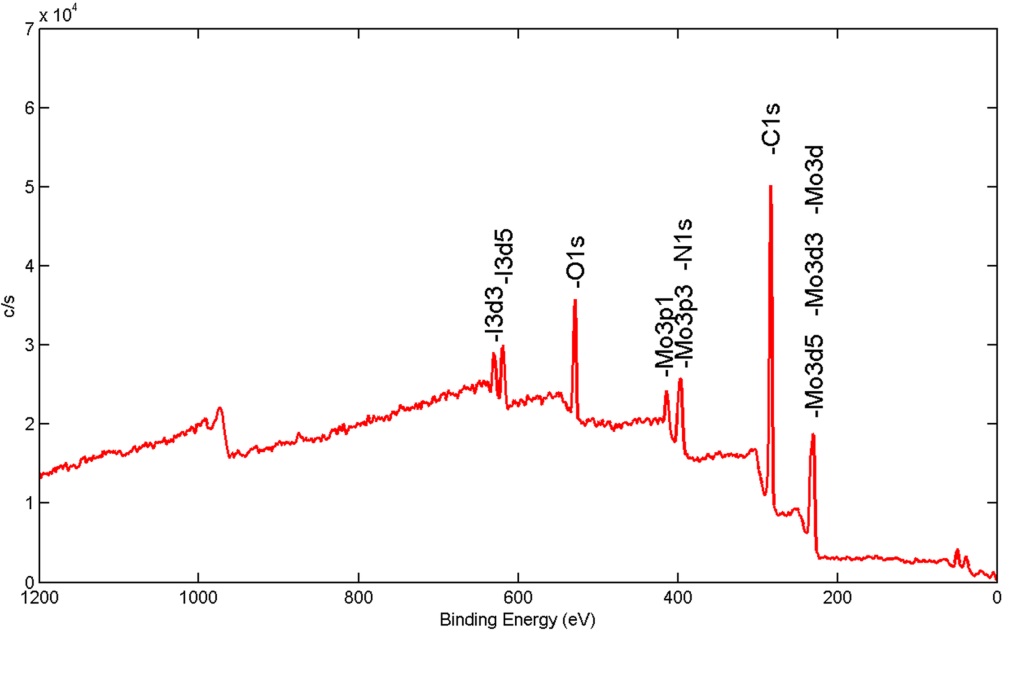


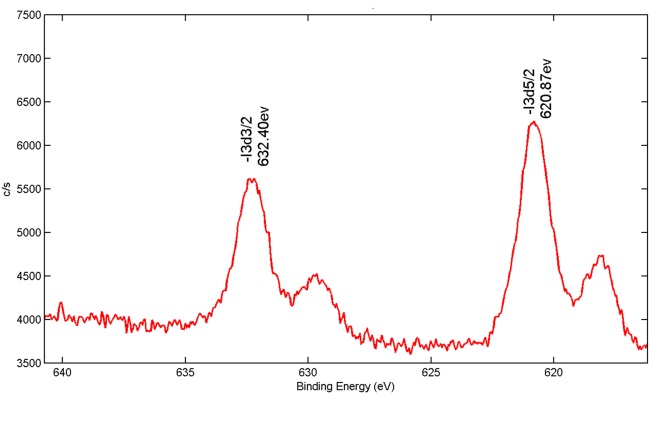

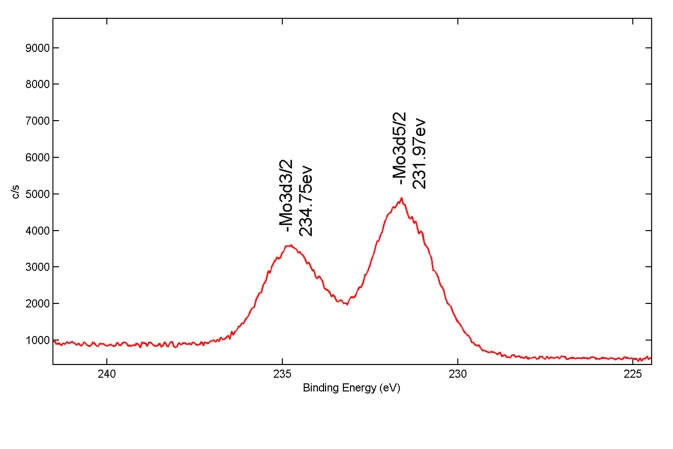


Figure 12 Full XPS spectra of compound **2** and narrow XPS spectra for I(3d) and Mo(3d).


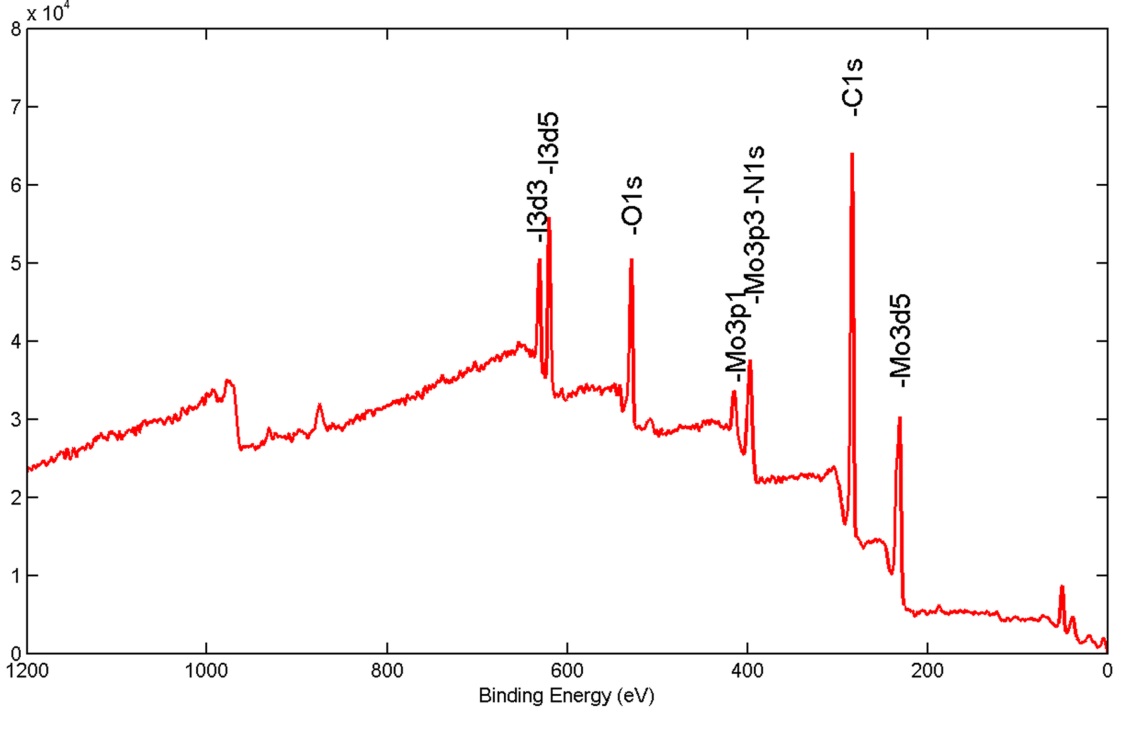


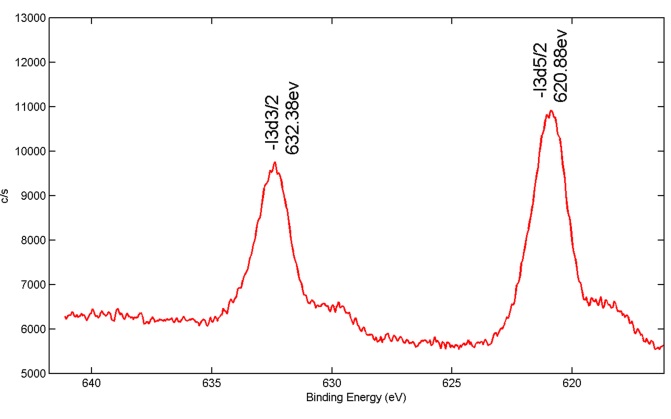

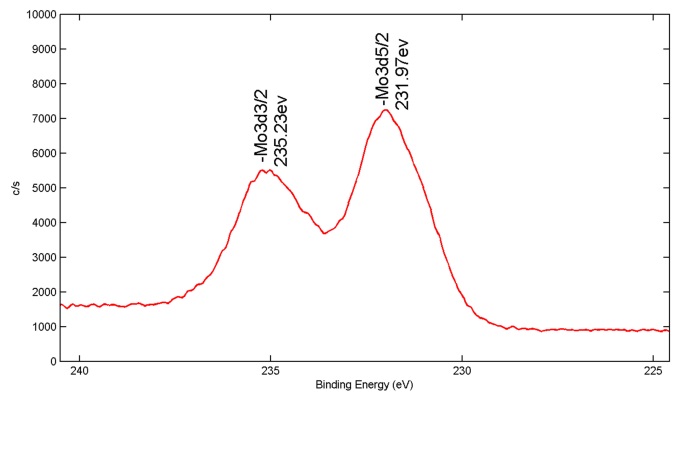


Figure 13 Full XPS spectra of compound **3** and narrow XPS spectra for I(3d) and Mo(3d).


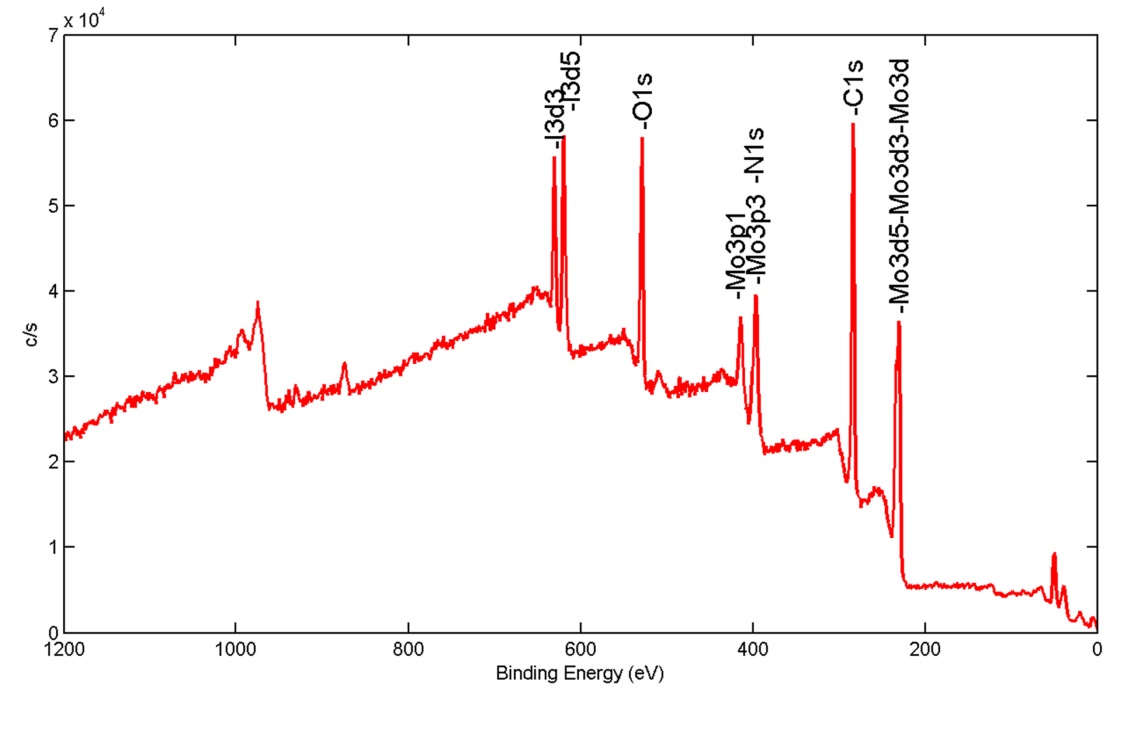


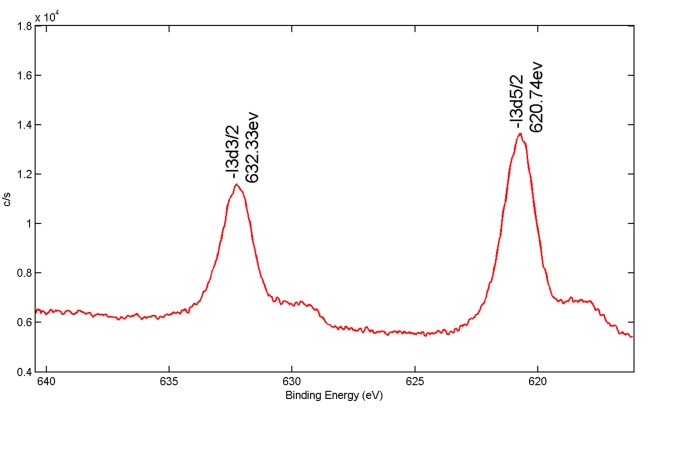

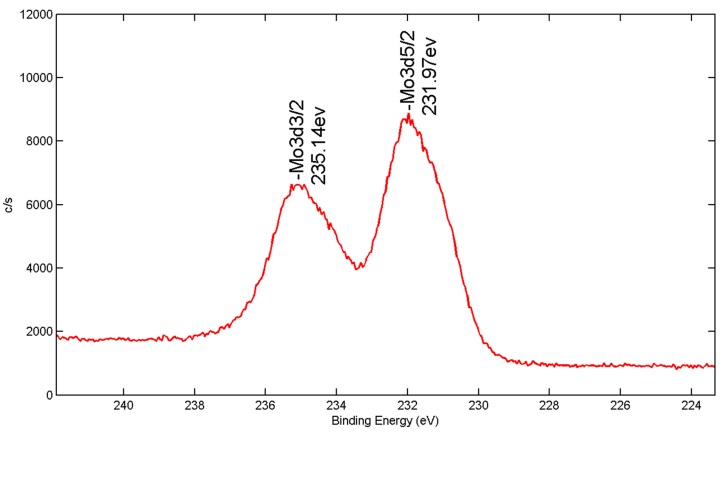


Figure 14 Full XPS spectra of compound **4** and narrow XPS spectra for I(3d) and Mo(3d).


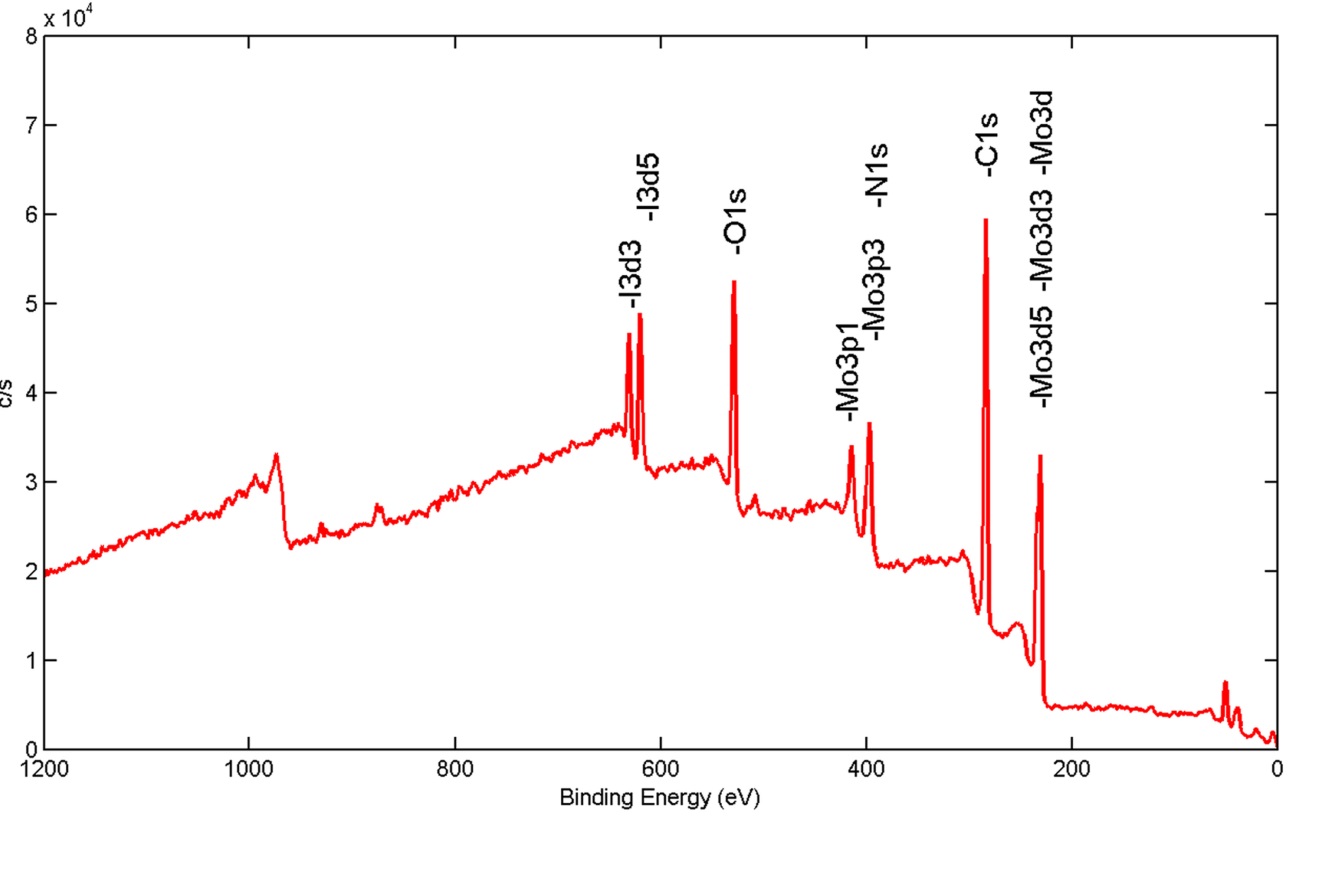


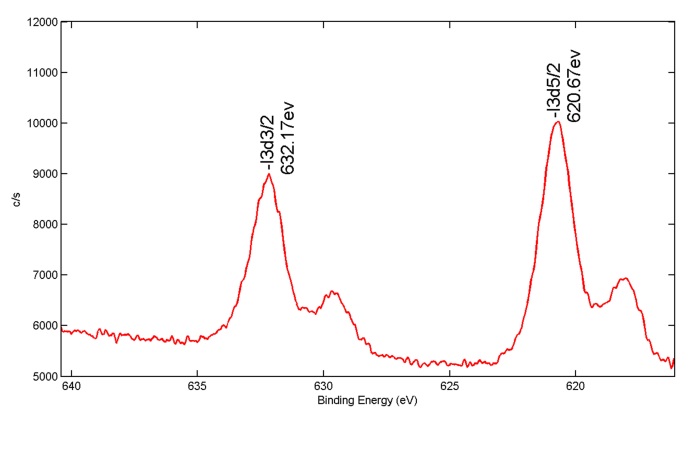

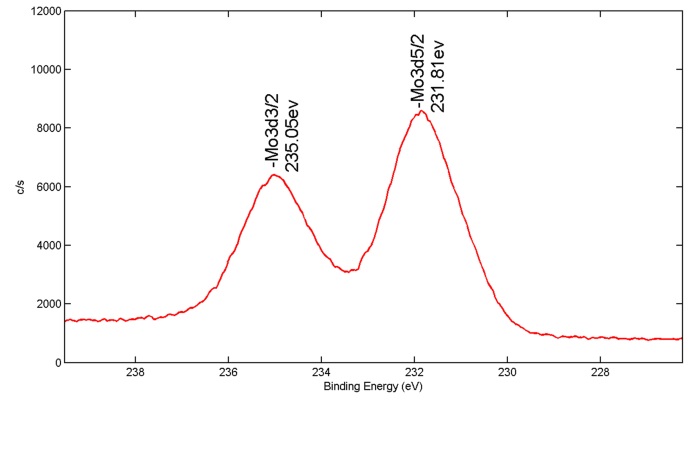


Figure 15 Full XPS spectra of compound **5** and narrow XPS spectra for I(3d) and Mo(3d).


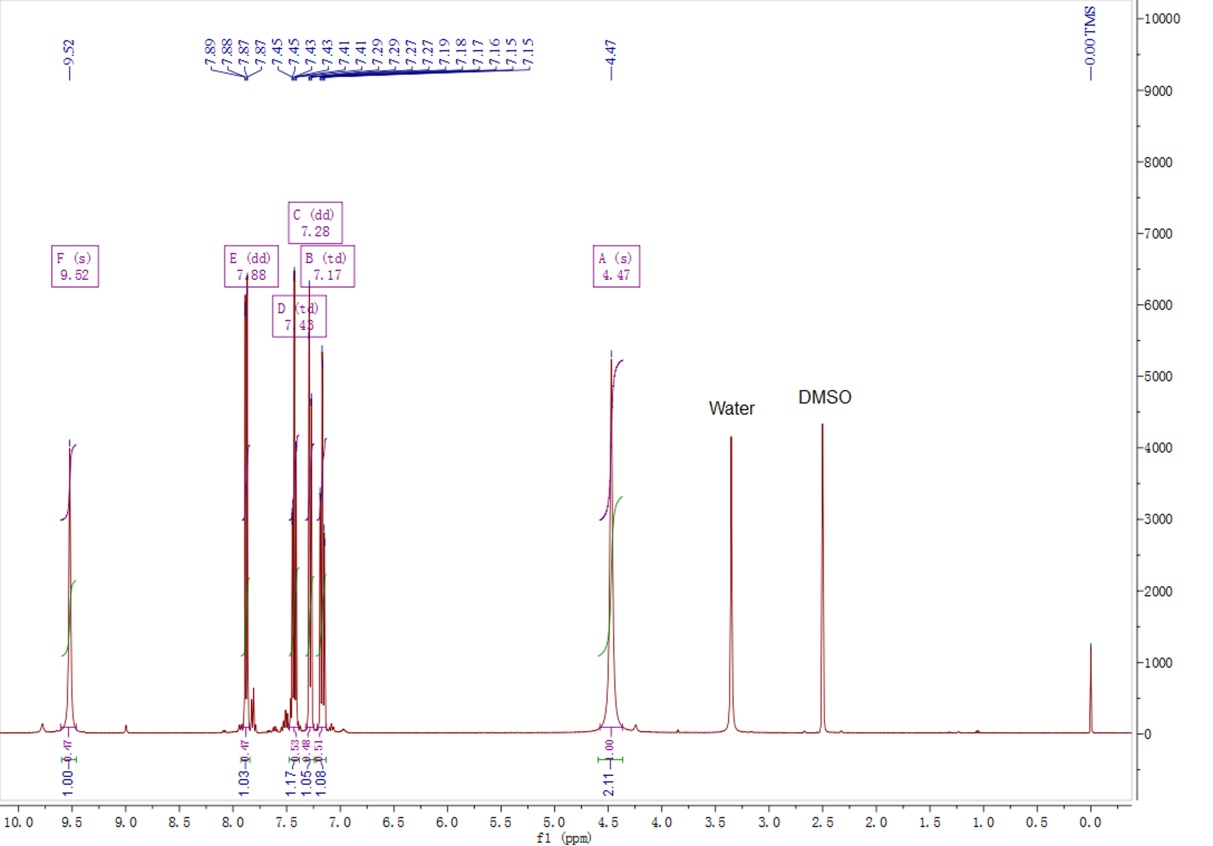


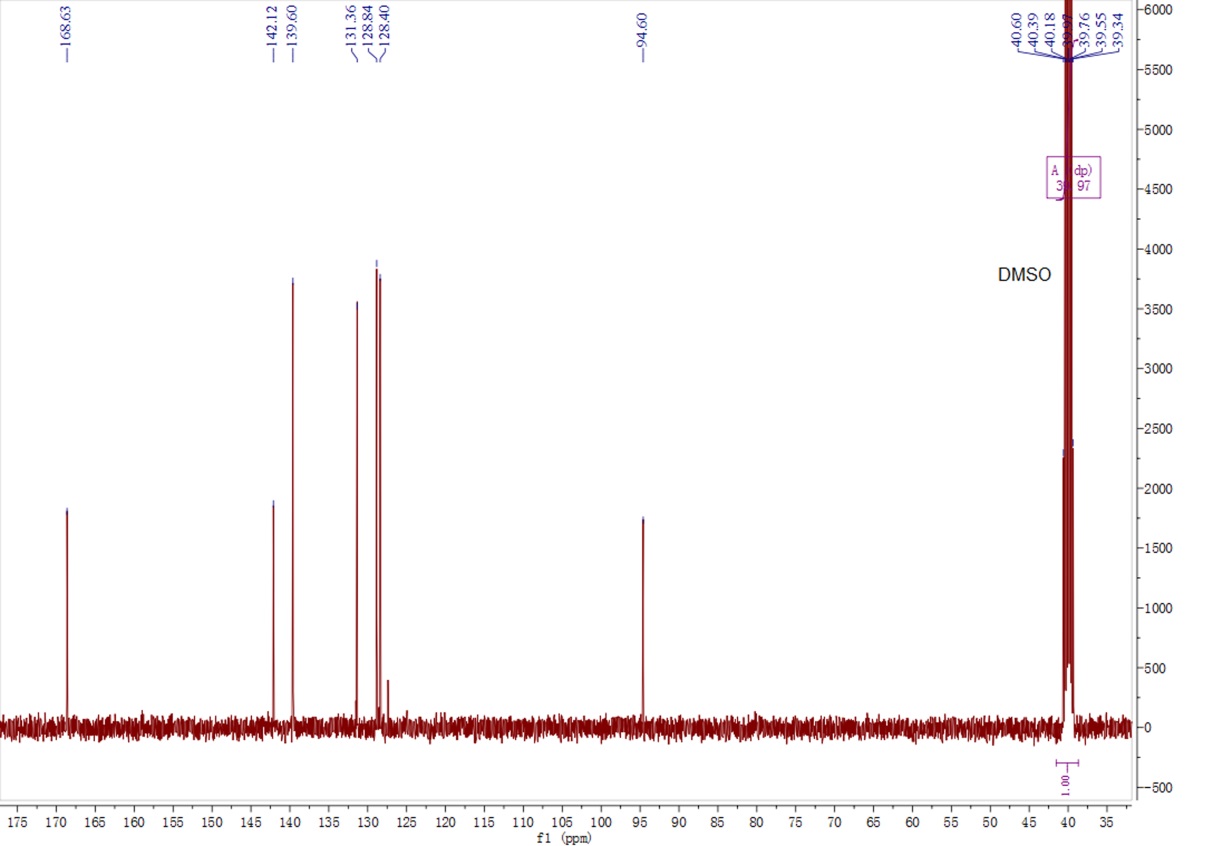


**Figure S16** ^1^H NMR and ^13^C NMR spectrum of 2-iodobenzohydrazide (**L1**).

**
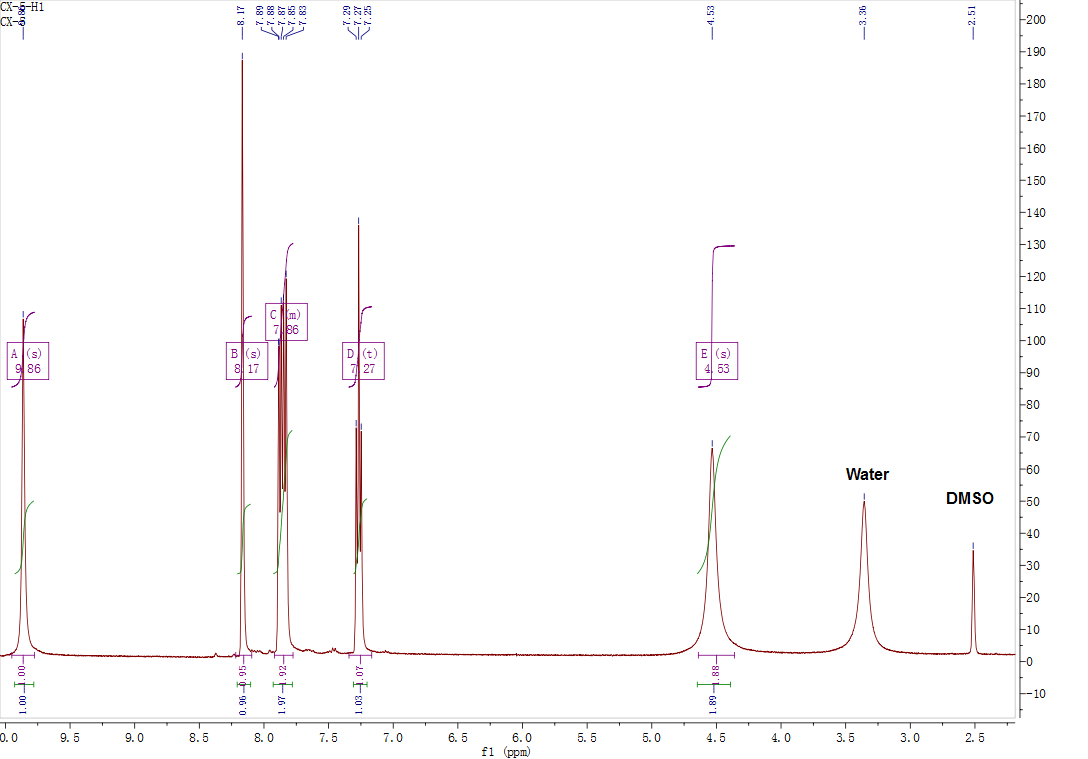
**

**
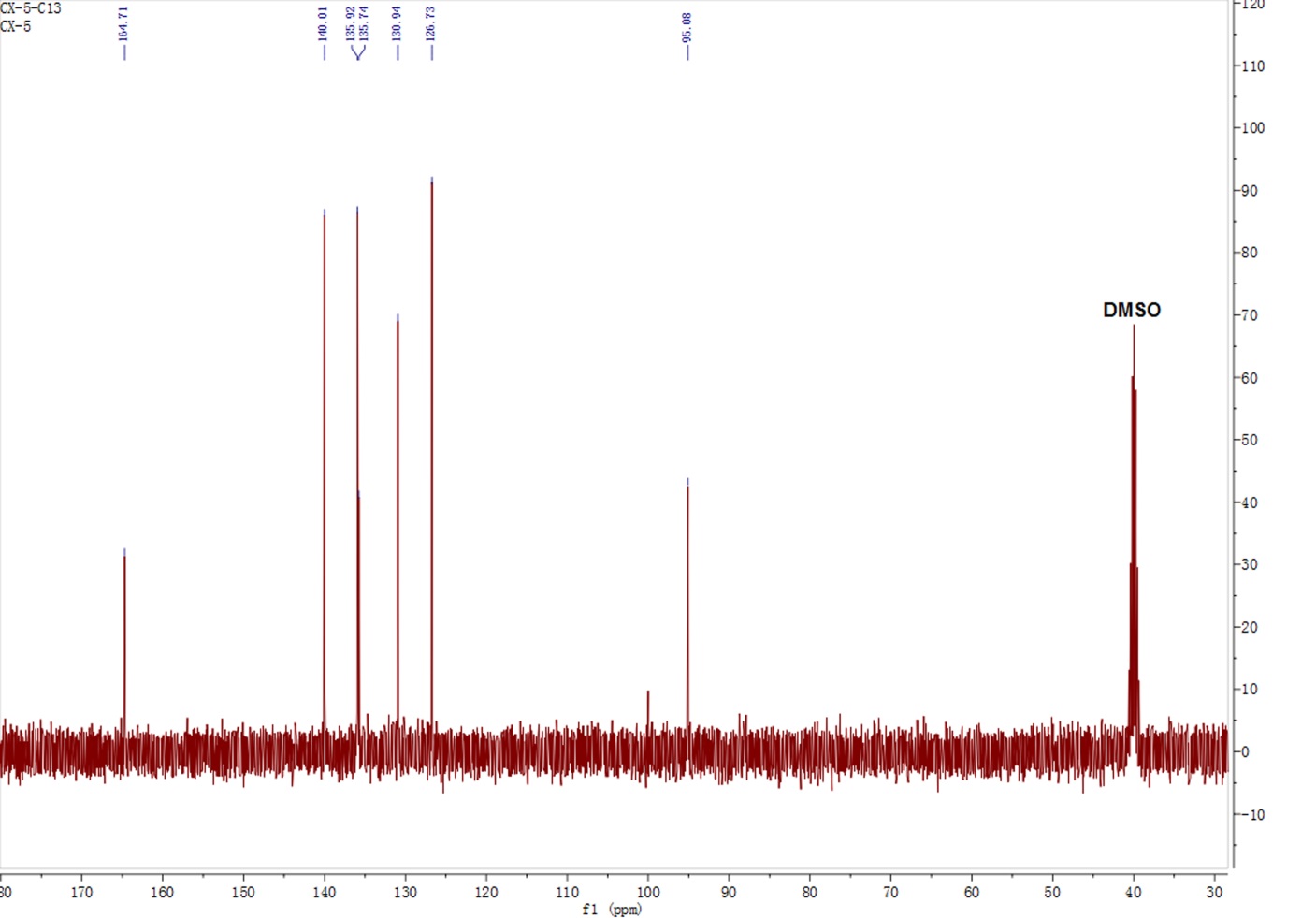
**

Figure S17 ^1^H NMR and ^13^C NMR spectrum of 3-iodobenzohydrazide (**L2**)

**
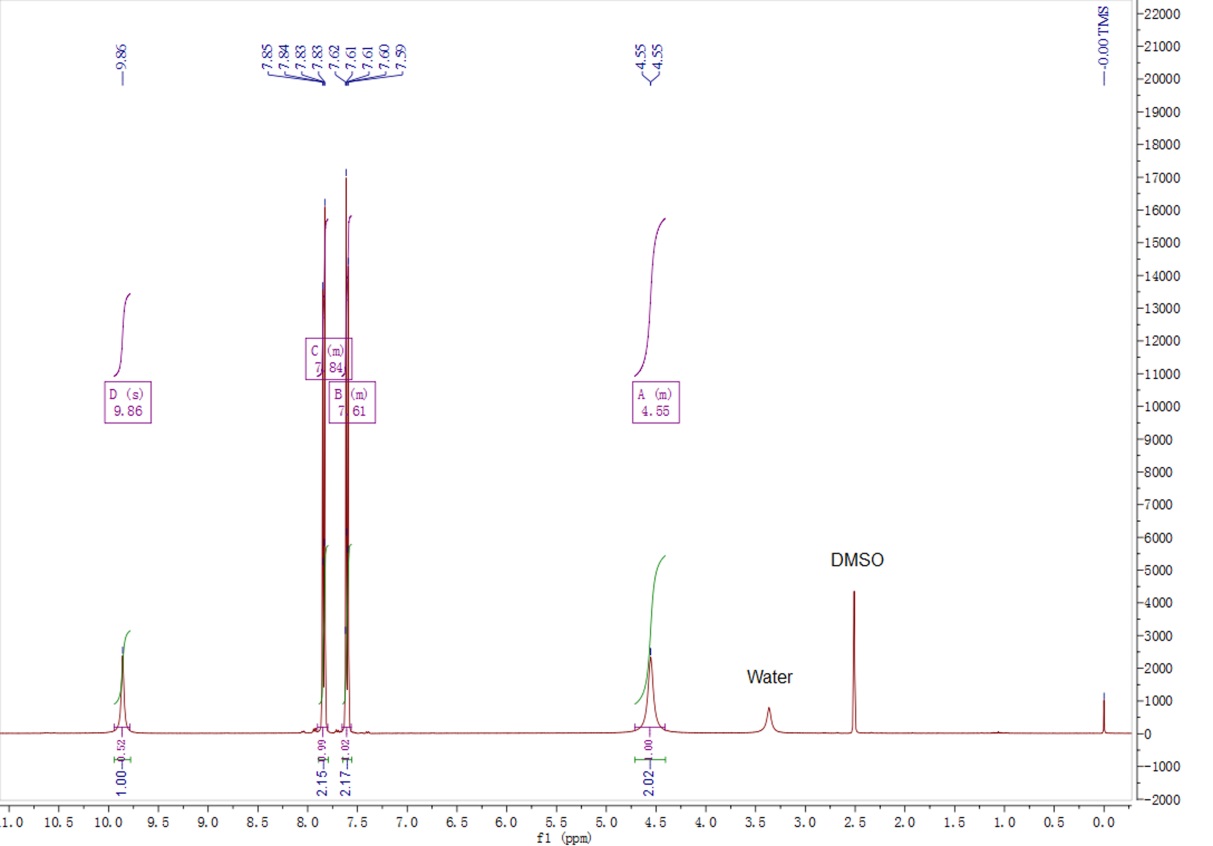
**

**
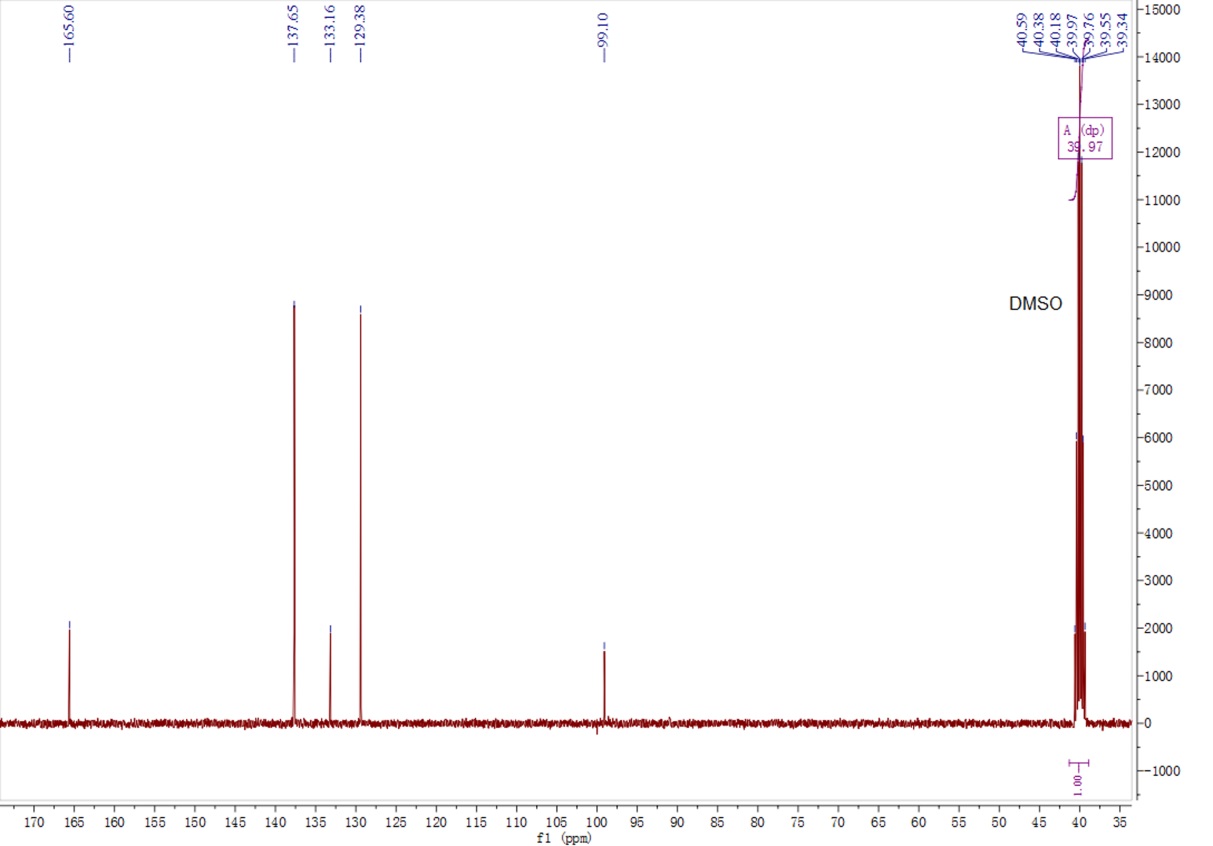
**

Figure S18 ^1^H NMR and ^13^C NMR spectrum of 4-iodobenzohydrazide (**L3**).

**
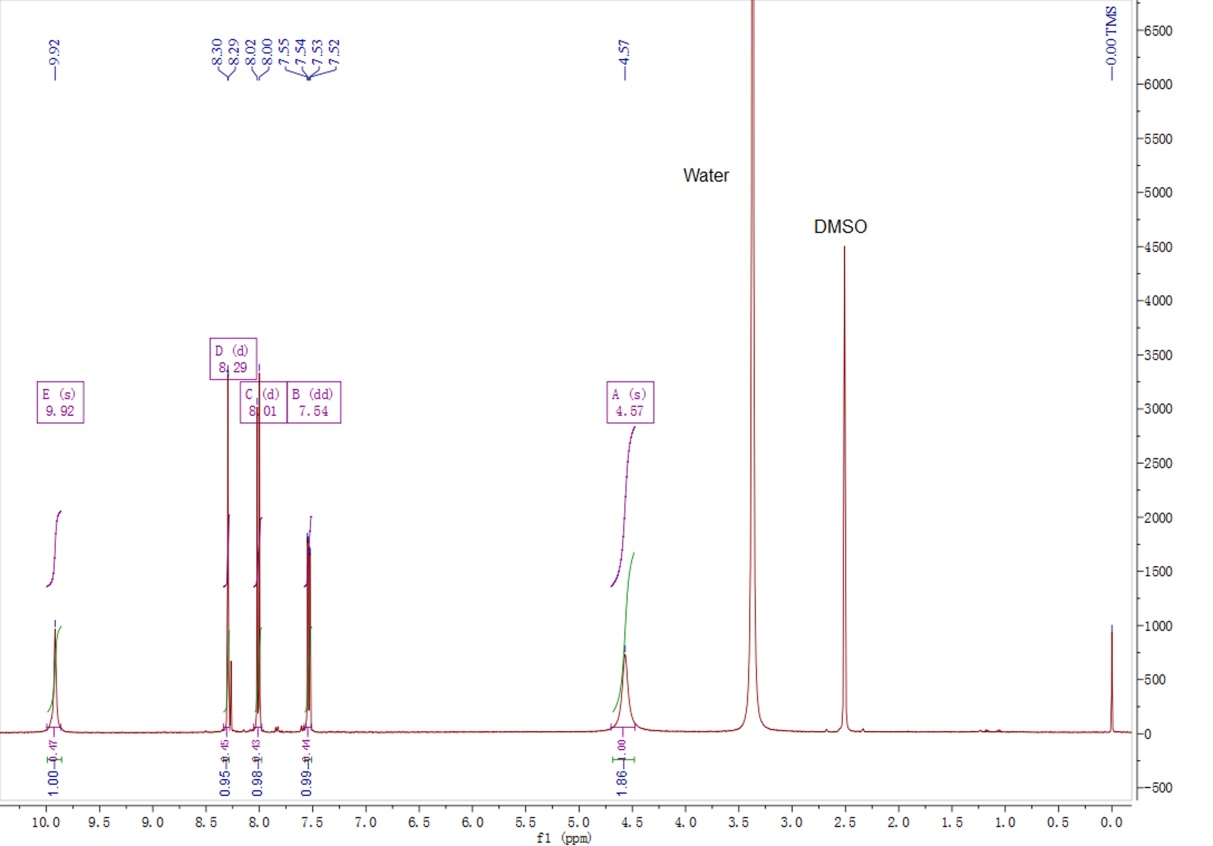
**

**
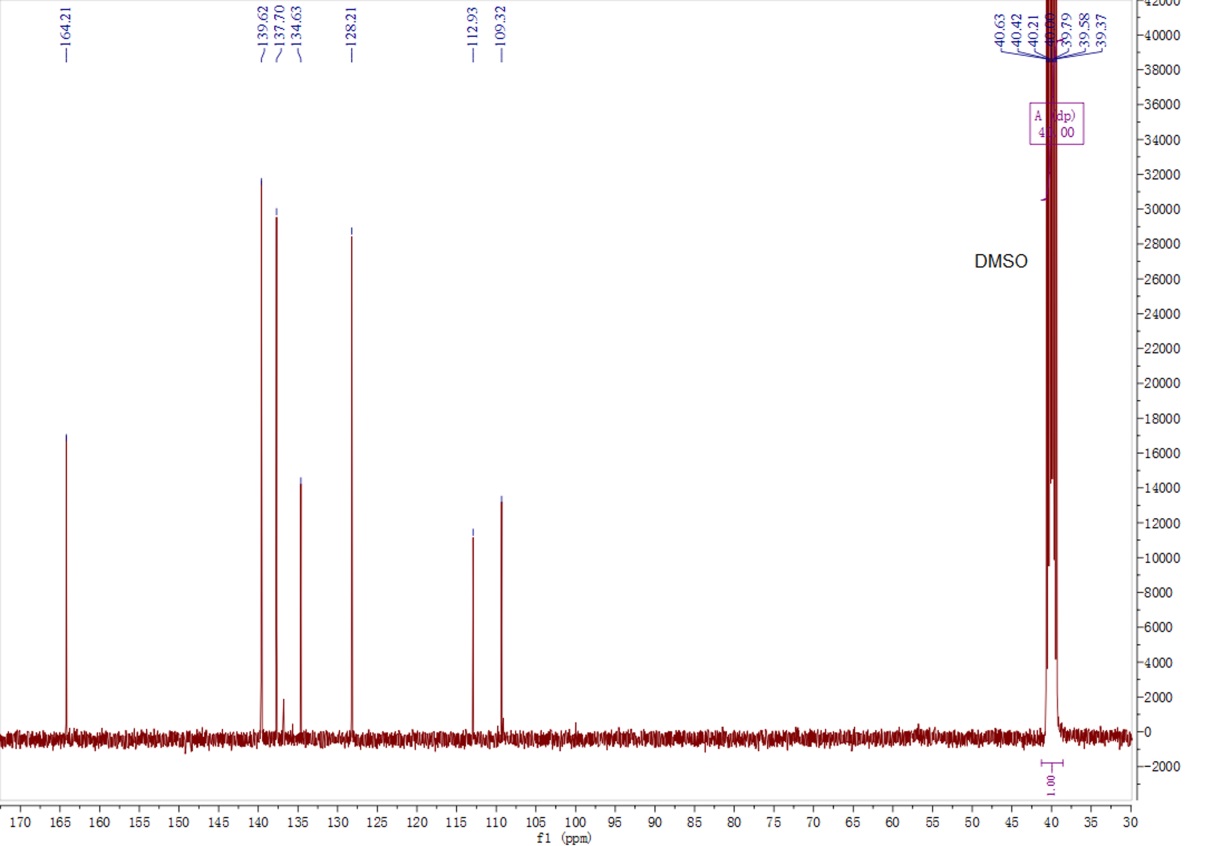
**

Figure S19 ^1^H NMR and ^13^C NMR spectrum of 3**,**4-Diiodobenzohydrazide (**L4**).

**
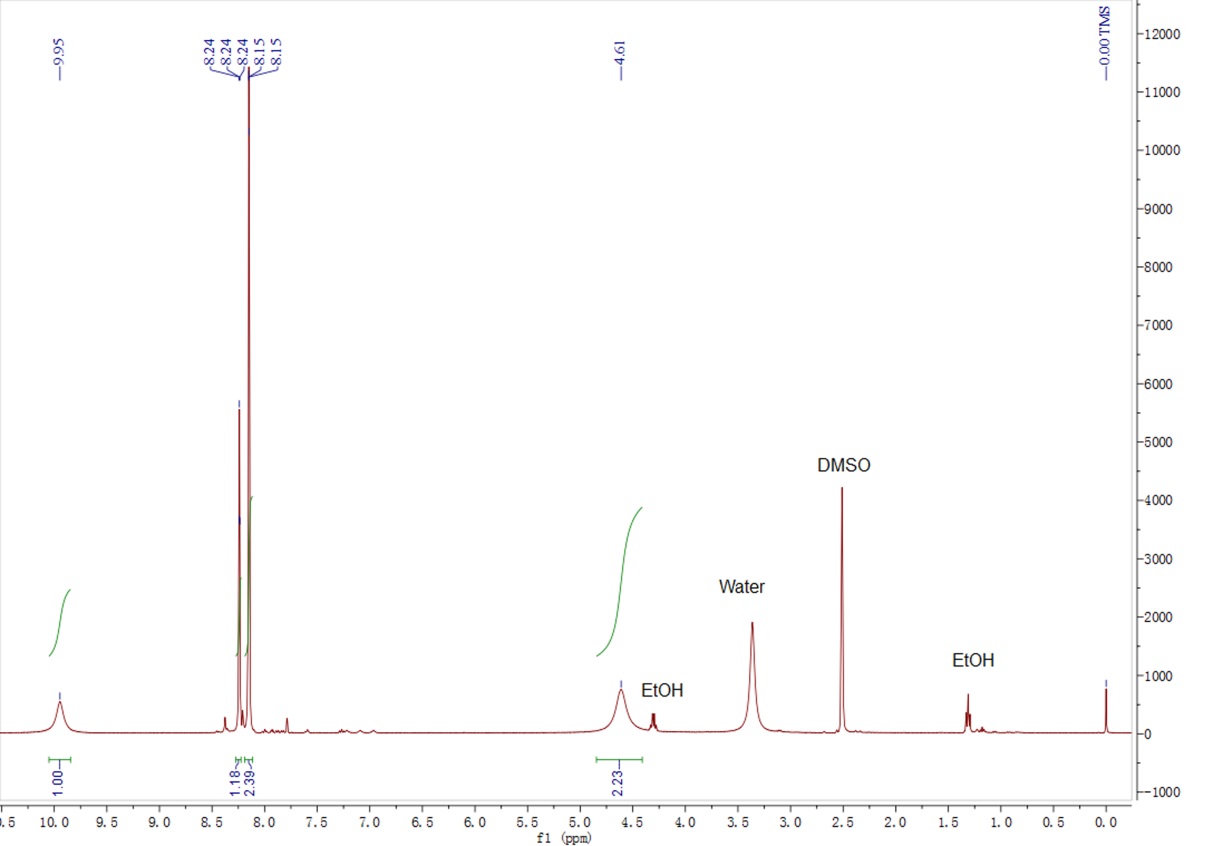
**

**
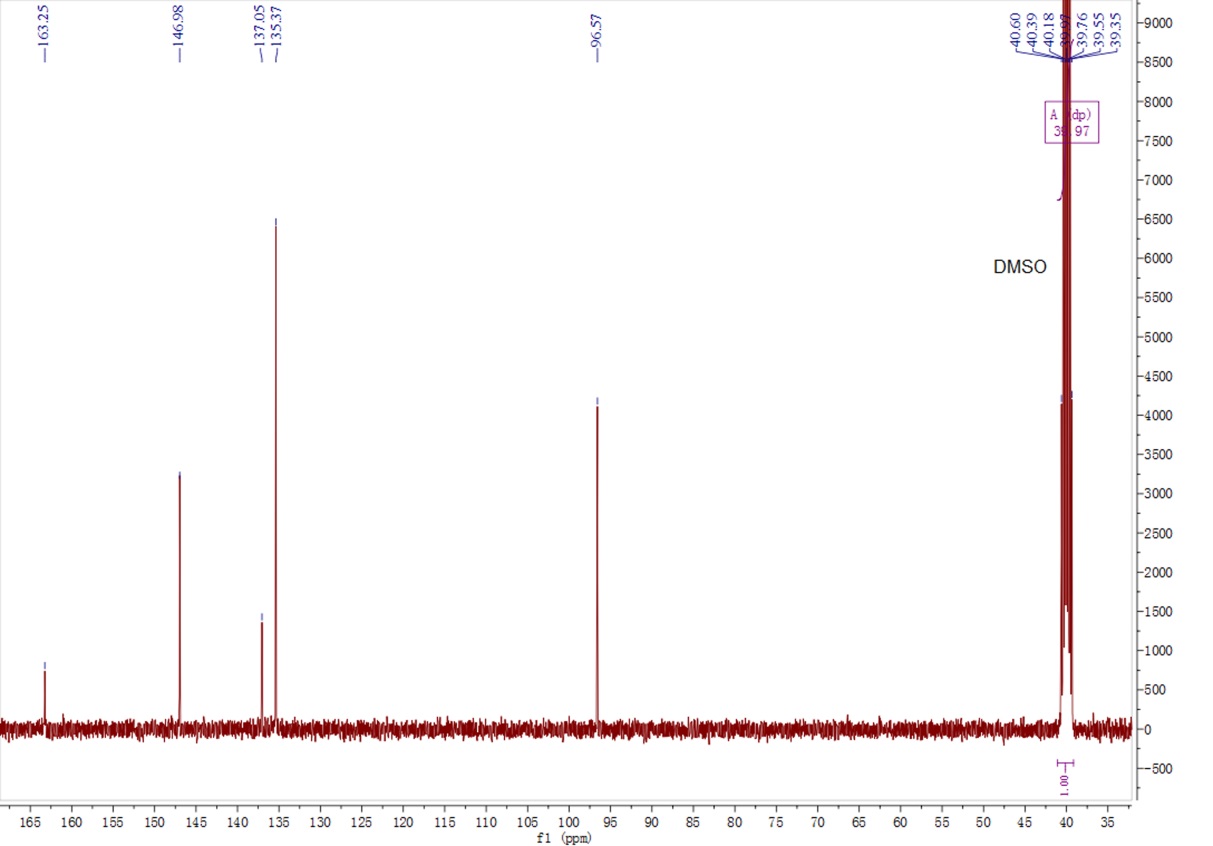
**

Figure S20 ^1^H NMR and ^13^C NMR spectrum of 2**,**3**,**5-Triiodobenzenecarbohydrazide (**L5**).

**
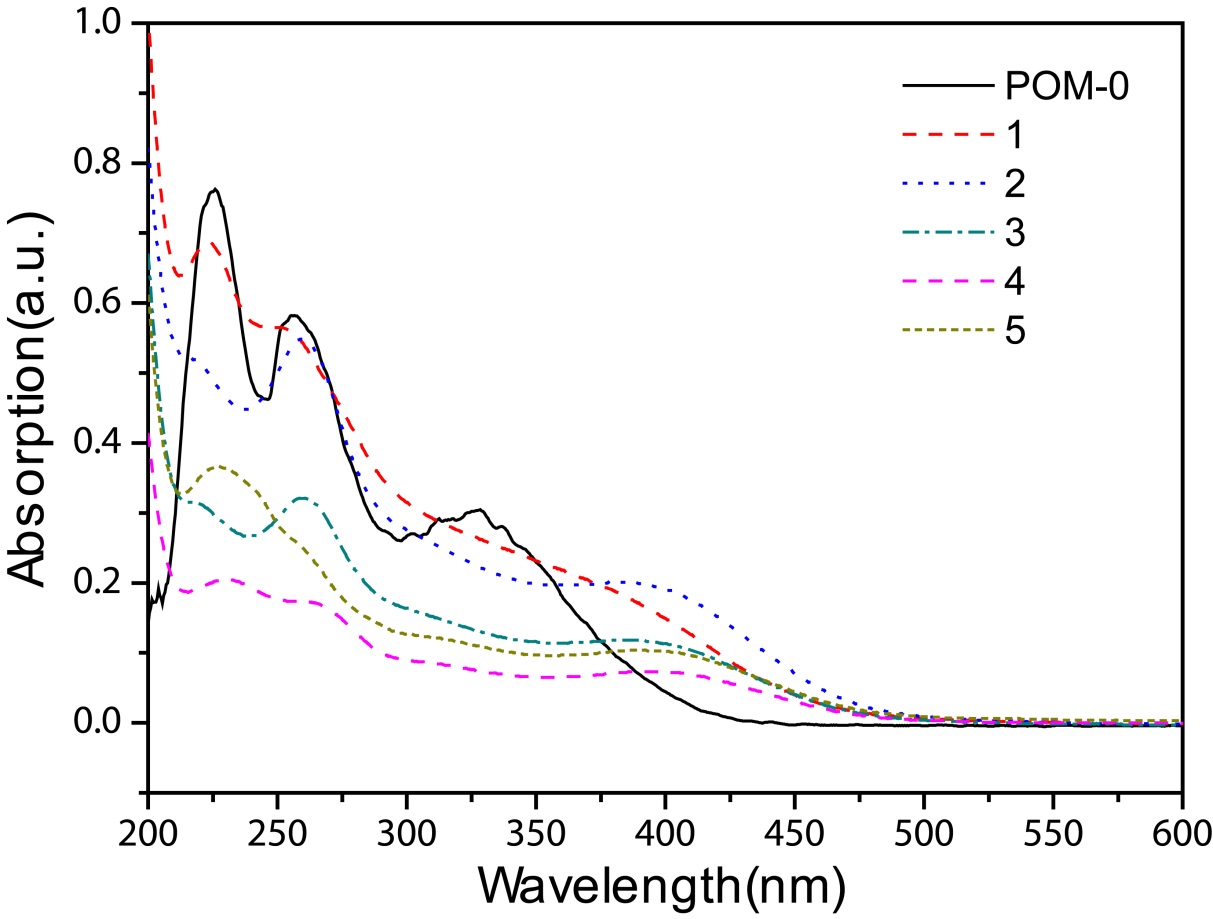
**

**Figure S21** UV-Vis spectra of **POM-0** and compounds **1**-**5**.


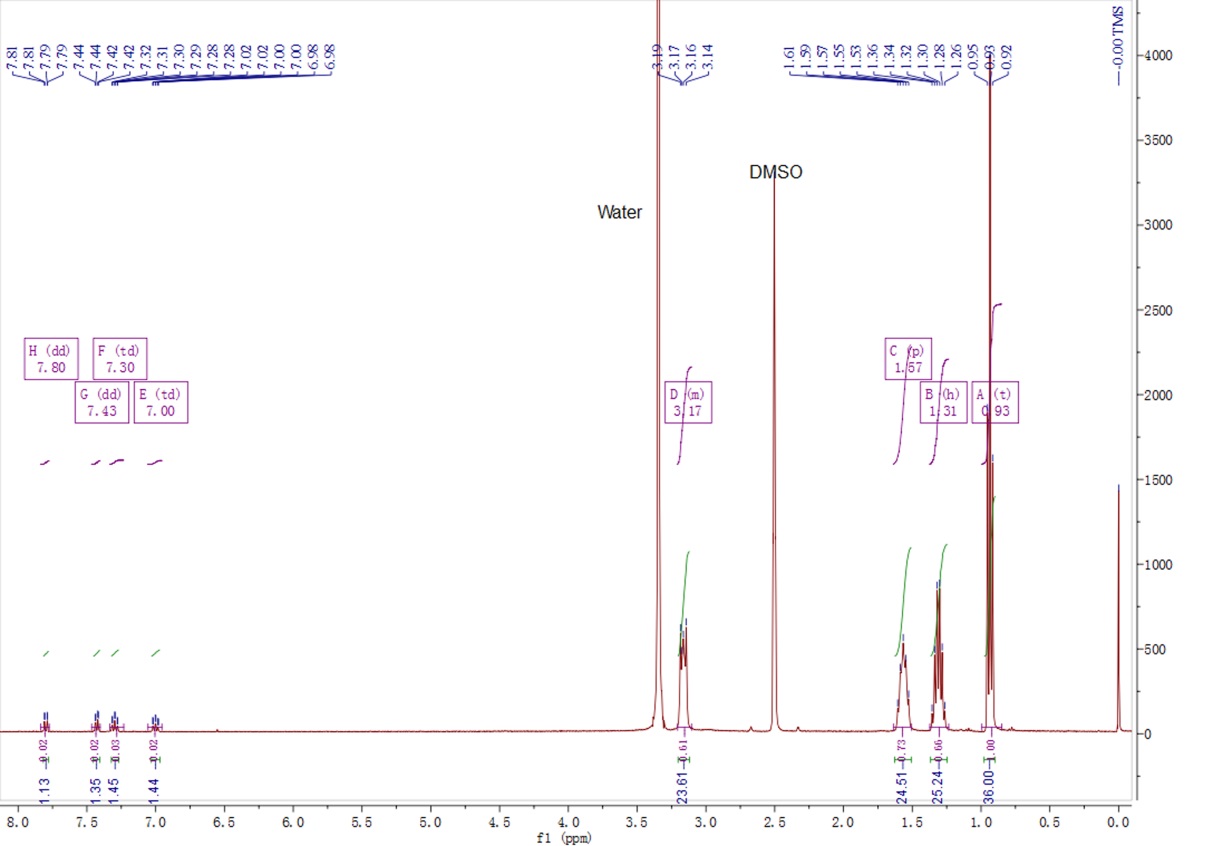


**Figure S22** ^1^H NMR of [Bu_4_N]_3_[Mo_6_O_18_(=N=NCOC_6_H_4_-2-I)] (**1**)


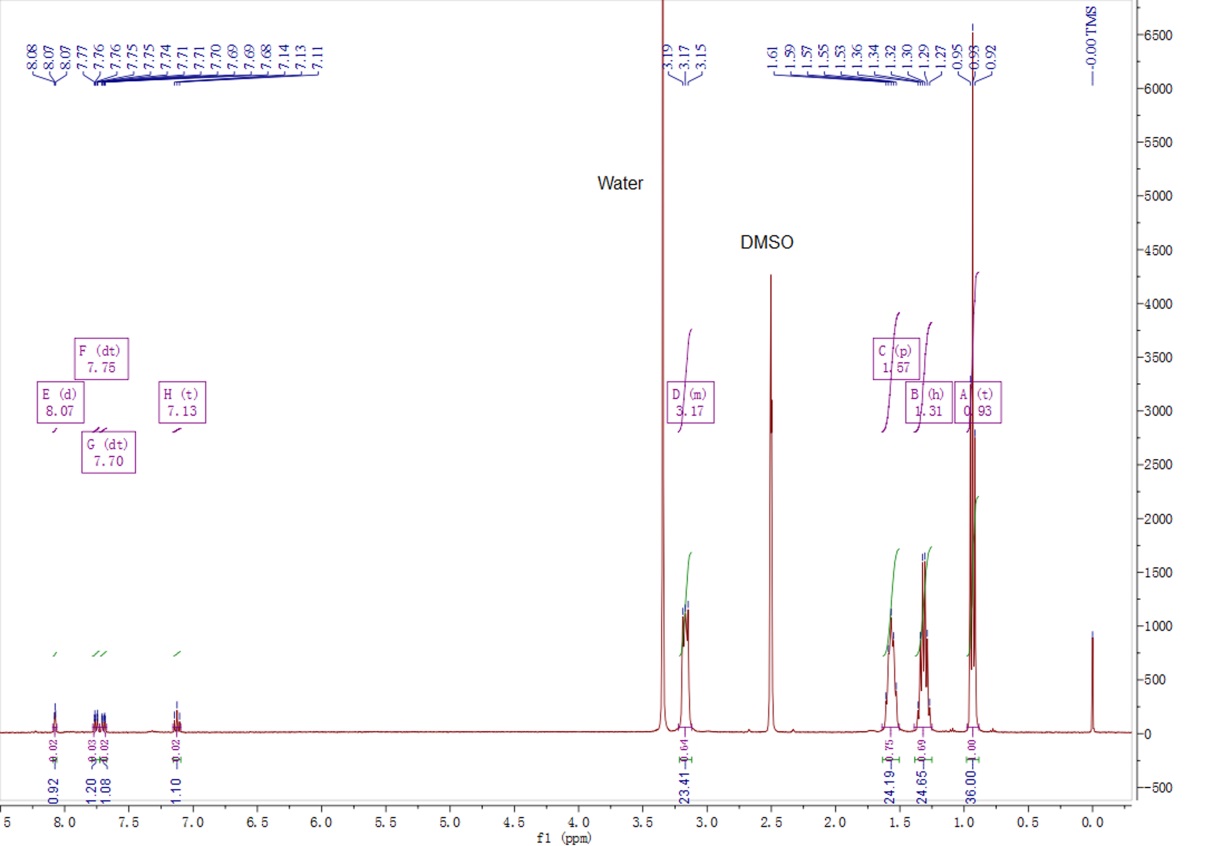


**Figure S23** ^1^H NMR of [Bu_4_N]_3_[Mo_6_O_18_(=N=NCOC_6_H_4_-3-I)] (**2**)


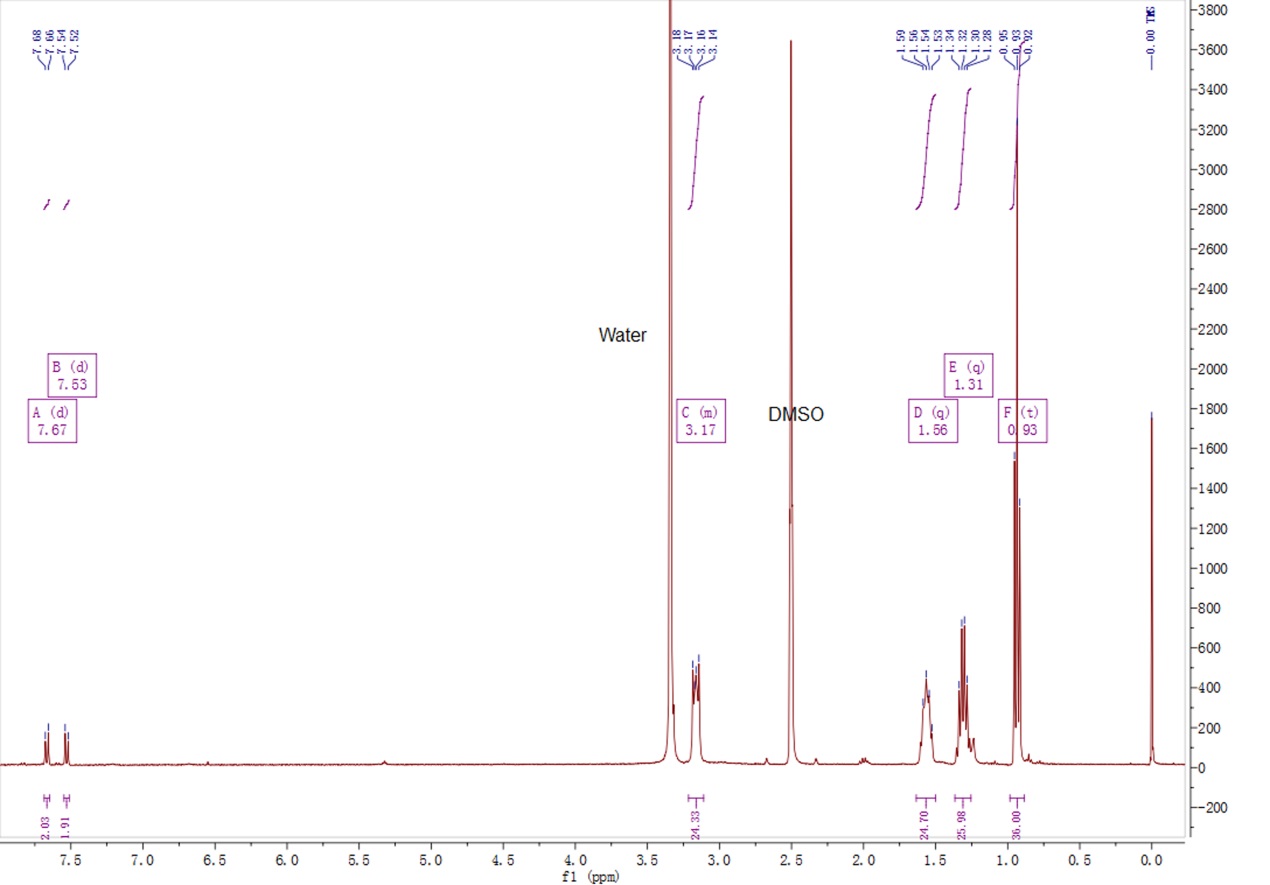


**Figure S24** ^1^H NMR of [Bu_4_N]_3_[Mo_6_O_18_(=N=NCOC_6_H_4_-4-I)] (**3**)


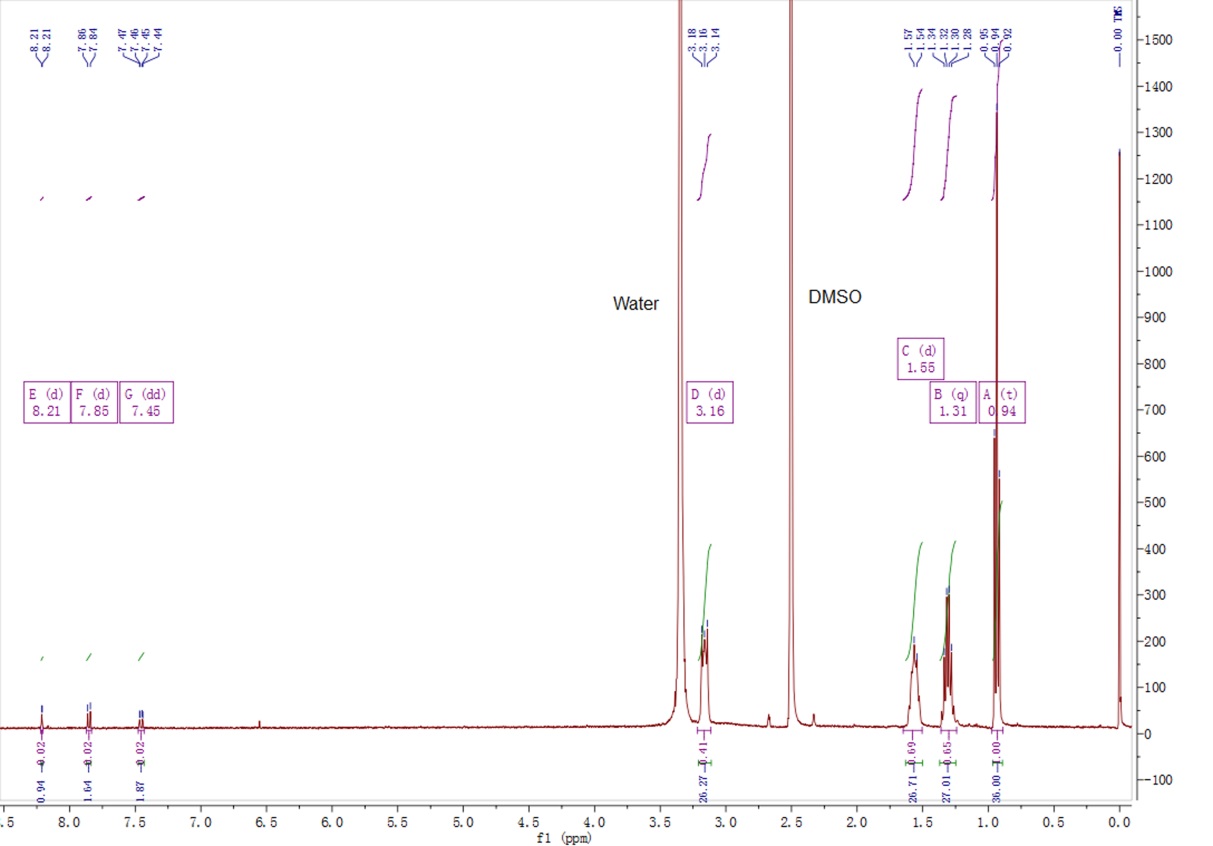


**Figure S25** ^1^H NMR of [Bu_4_N]_3_[Mo_6_O_18_(=N=NCOC_6_H_3_-3,4-I_2_)] (**4**)


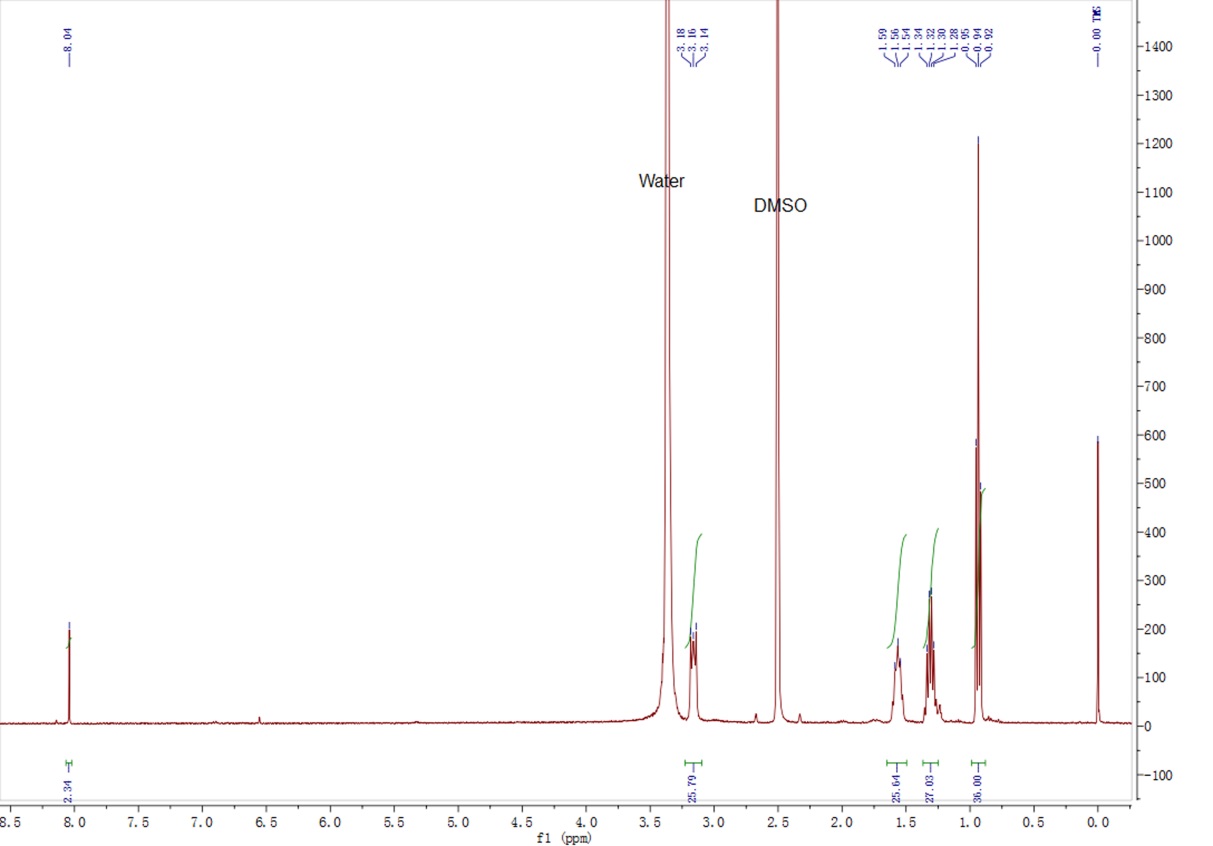


**Figure S26** ^1^H NMR of [Bu_4_N]_3_[Mo_6_O_18_(=N=NCOC_6_H_2_-2,3,5-I_3_)] (**5**)


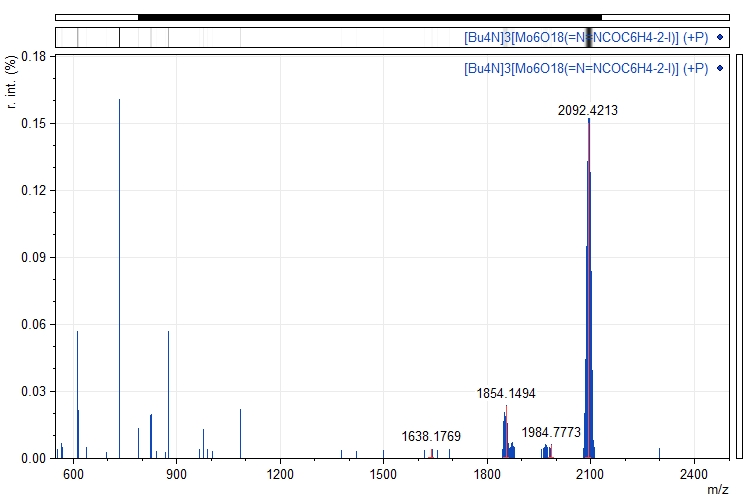
**
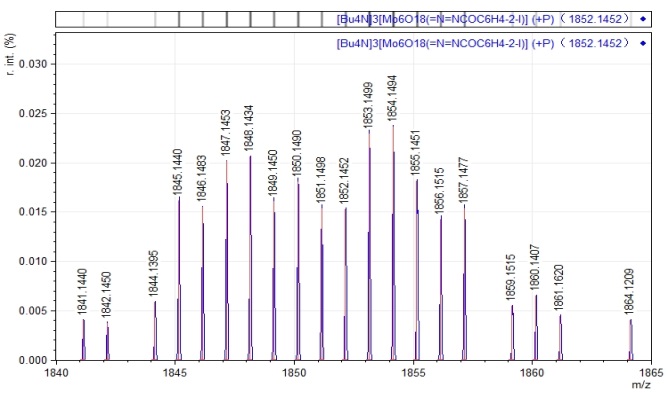

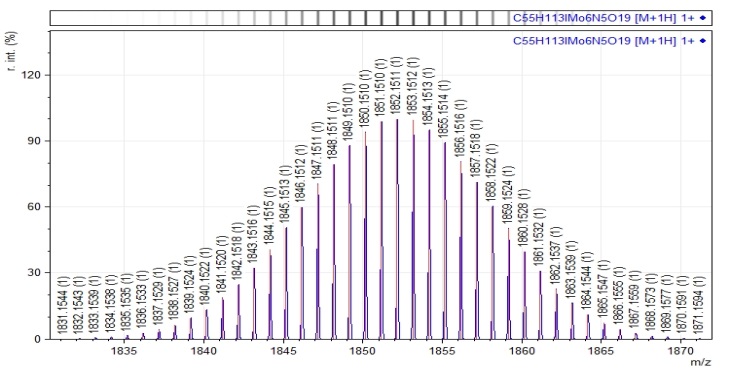
**

**
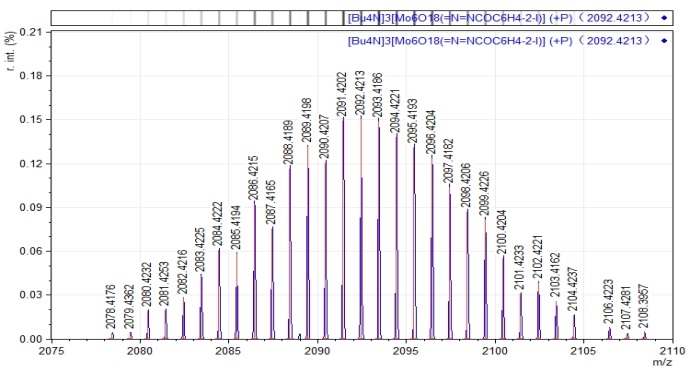

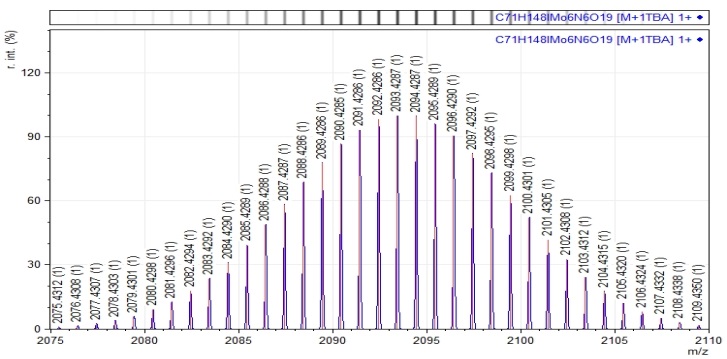
**

Figure **S27.** GC-HR MS spectrum (positive region) of [Bu_4_N]_3_[Mo_6_O_18_(=N=NCOC_6_H_4_-2-I)] (**1**): 1852.1452 for {M + 1H}^1+^, calcd. 1852.1511; 2092.4213 for {M + 1TBA}^1+^, calcd.2092.4286


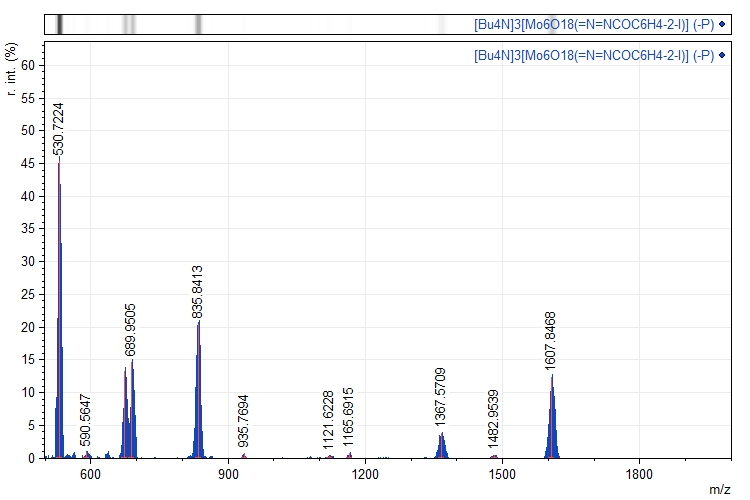


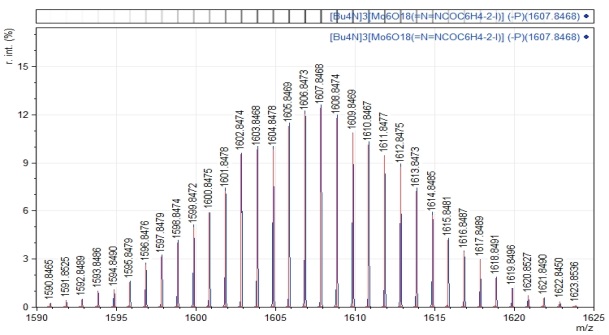

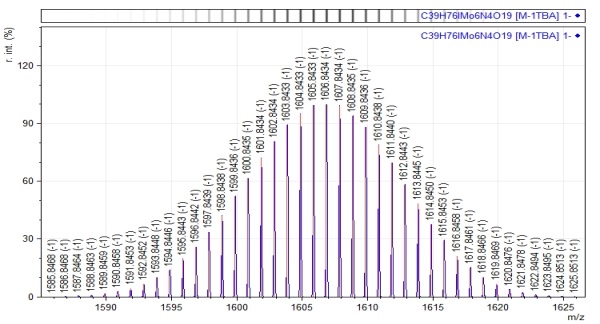


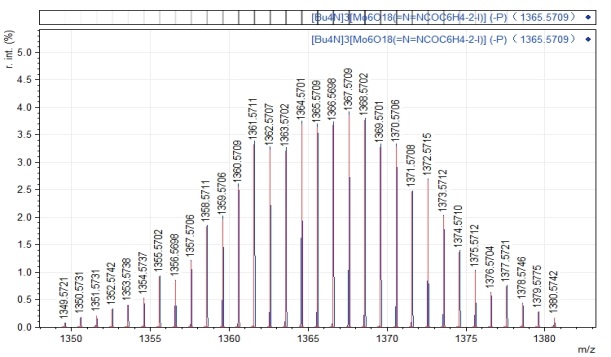

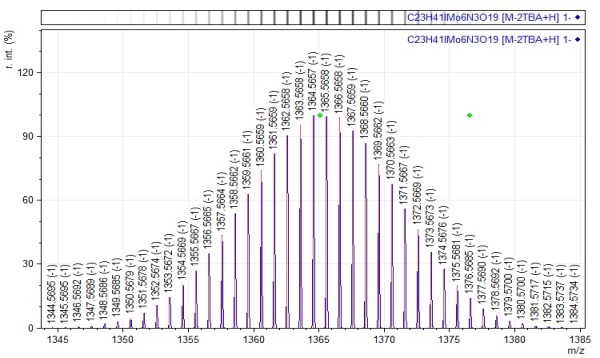


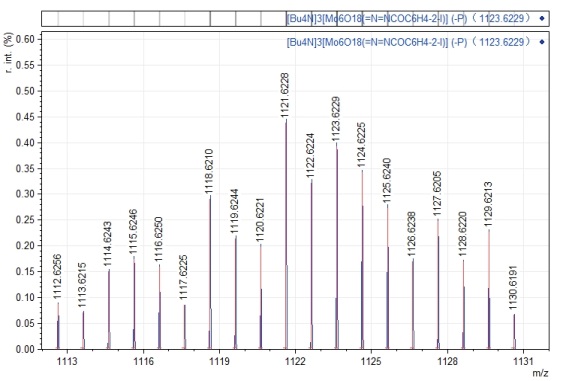

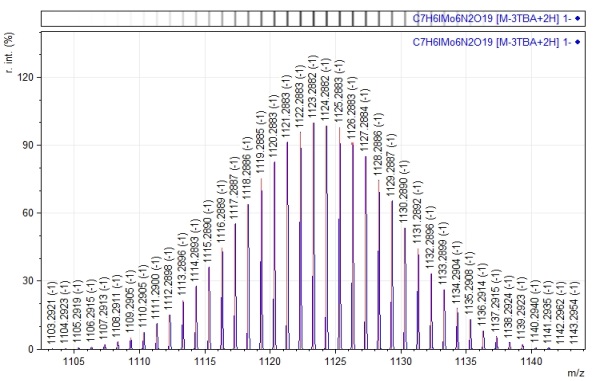


**Figure S28**. GC-HR MS spectrum (negative region) of [Bu_4_N]_3_[Mo_6_O_18_(=N=NCOC_6_H_4_-2-I)] (**1**): 1607.8468 for {M - TBA}^1-^: calcd. 1607.8434; 1366.5709 for {M - 2TBA + H}^1-^: calcd.1366.5658; 1123.6229 for {M - 3TBA + 2H}^1-^1123.2882.

Table S2 Summary of GC-HRMS results of [Bu_4_N]_3_[Mo_6_O_18_(=N=NCOC_6_H_4_-2-I)] (**1**)**.**

| Negative mode of GC-HRMS for**[Bu_4_N]_3_[Mo_6_O_18_(=N=NCOC_6_H_4_-2-I)]** | | | | |
| --- | --- | --- | --- | --- |
| m/z | Calculated m/z | Assigned Species | Charge | Notes |
| 1607.8468 | 1607.8434 | {M- TBA}^1-^ | -1 | molecule ion |
| 1366.5709 | 1366.5658 | {M- 2TBA + H}^1-^ | -1 | molecule ion |
| 1123.6229 | 1123.2882 | {M- 3TBA +2 H}^1-^ | -1 | molecule ion |
|  |  |  |  |  |
| Positive mode of GC-HRMS for **[Bu_4_N]_3_[Mo_6_O_18_(=N=NCOC_6_H_4_-2-I)]** | | | | |
| 1852.1452 | 1852.1511 | {M + 1H}^1+^ | +1 | molecule ion |
| 2092.4213 | 2092.4286 | {M + 1TBA}^1+^ | +1 | molecule ion |


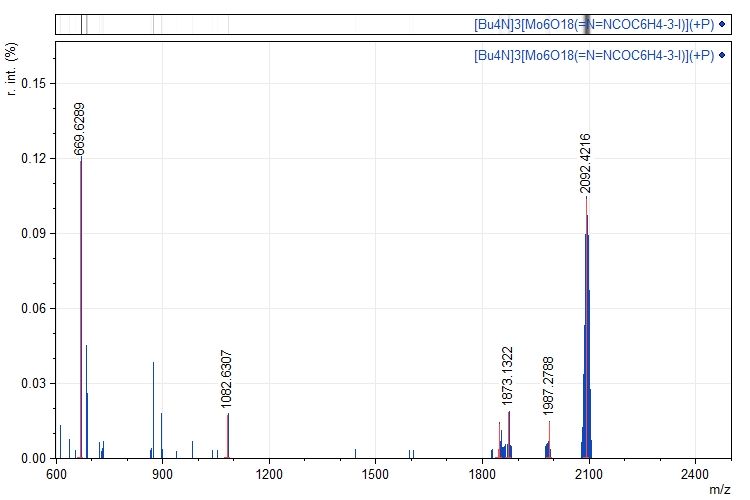


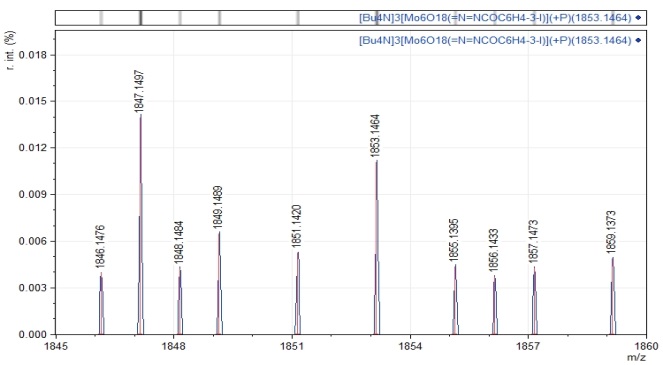
**
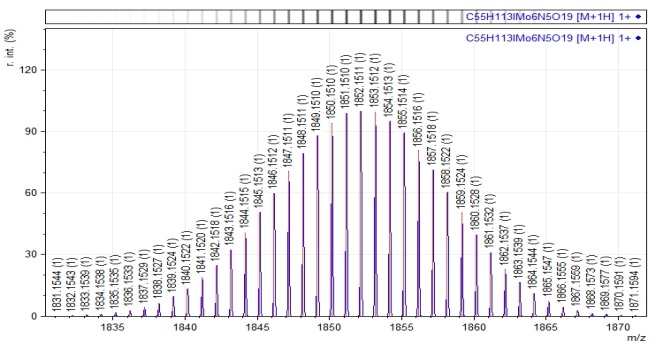
**


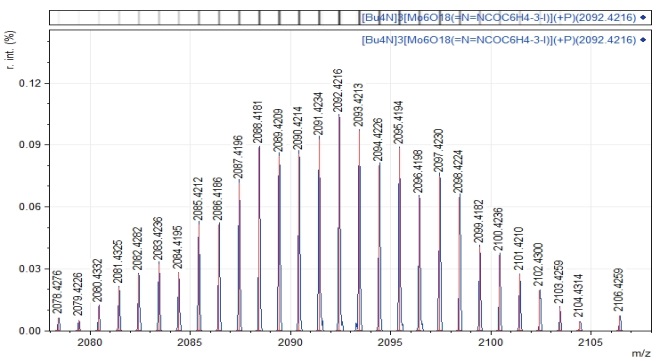
**
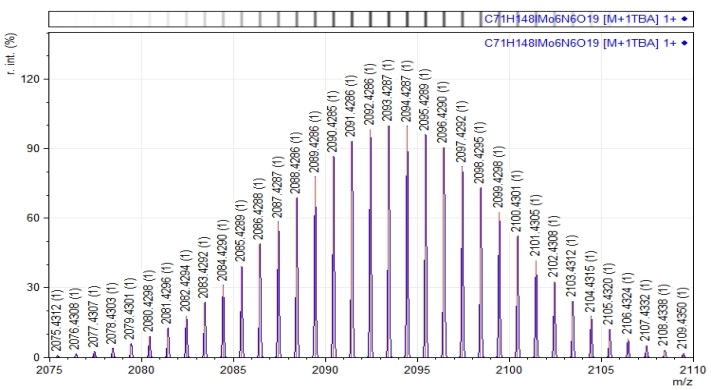
**

**Figure S29**. GC-HR MS spectrum (positive region) of [Bu_4_N]_3_[Mo_6_O_18_(=N=NCOC_6_H_4_-3-I)] (**2**): 1853.1464 for {M + 1H}^1+^, calcd. 1853.1512; 2092.4216 for {M + 1TBA}^1+^, calcd.2092.4286


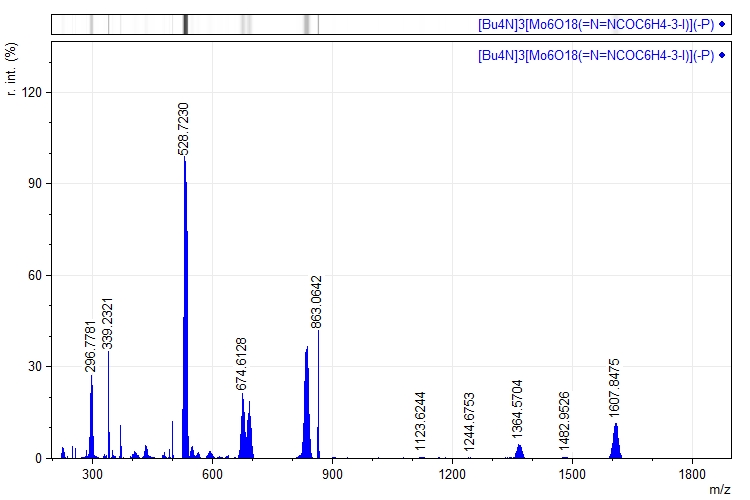


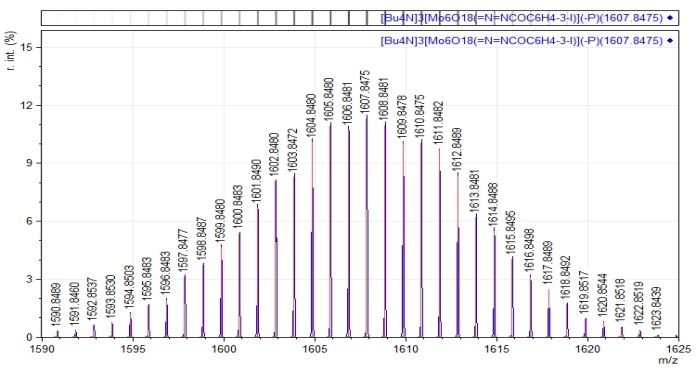

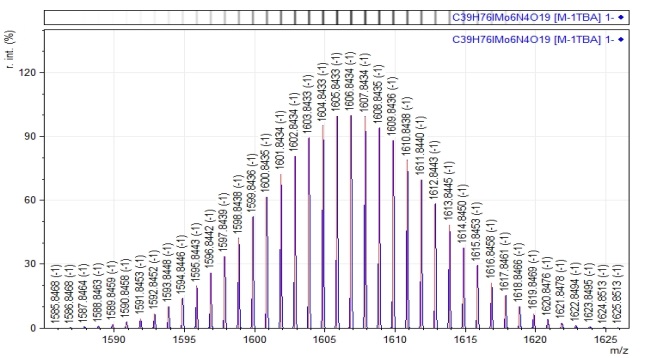


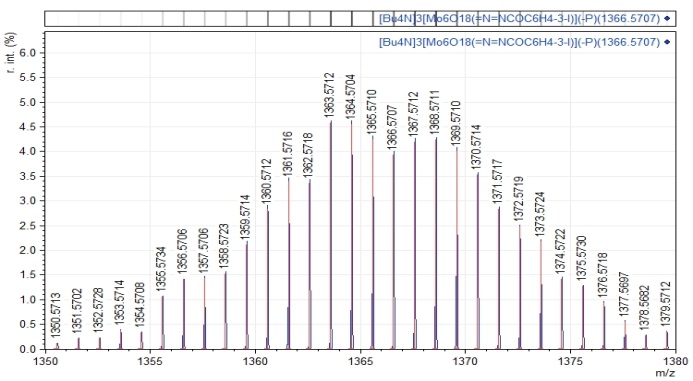

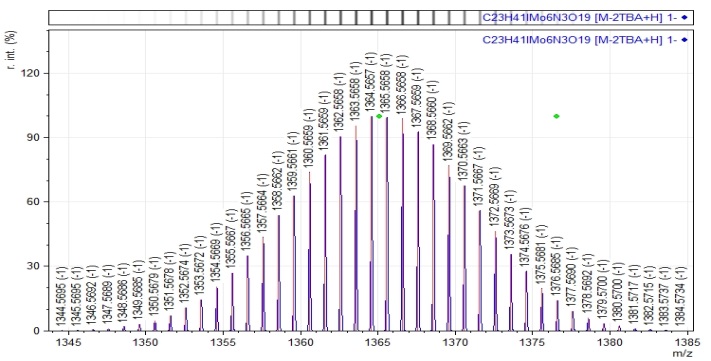


**
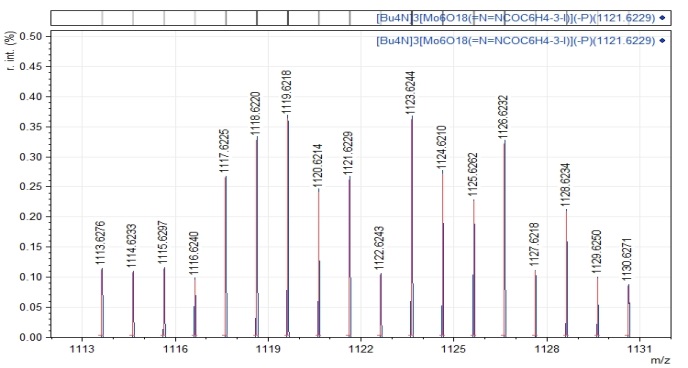
**
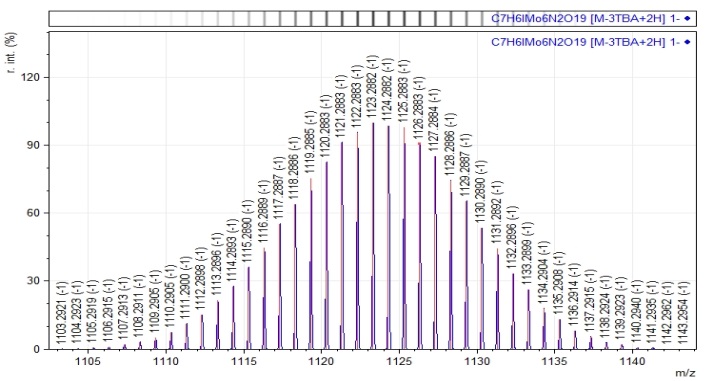


**Figure S30**. GC-HR MS spectrum (negative region) of [Bu_4_N]_3_[Mo_6_O_18_(=N=NCOC_6_H_4_-3-I)] (**2**): 2218.3156 for {M- TBA}^1-^: calcd.2218.3252; 1366.5709 for {M- 2TBA + H}^1-^: calcd.1366.5658; 1123.6229 for {M- 3TBA + 2H}^1-^: calcd.1123.2882.

Table S3 Summary of GC-HR MS results of [Bu_4_N]_3_[Mo_6_O_18_(=N=NCOC_6_H_4_-3-I)] (**2**)**.**

| Negative mode of GC-HR MS for[Bu_4_N]_3_[Mo_6_O_18_(=N=NCOC_6_H_4_-3-I)] | | | | |
| --- | --- | --- | --- | --- |
| m/z | Calculated m/z | Assigned Species | Charge | Notes |
| 1607.8475 | 1607.8434 | {M- TBA}^1-^ | -1 | molecule ion |
| 1366.5658 | 1366.5658 | {M- 2TBA + H}^1-^ | -1 | molecule ion |
| 1123.6229 | 1123.2882 | {M- 3TBA +2 H}^1-^ | -1 | molecule ion |
|  |  |  |  |  |
| Positive mode of GC-HR MS for [Bu_4_N]_3_[Mo_6_O_18_(=N=NCOC_6_H_4_-3-I)] | | | | |
| 1853.1464 | 1853.1512 | {M + 1H}^1+^ | +1 | molecule ion |
| 2092.4216 | 2092.4286 | {M + 1TBA}^1+^ | +1 | molecule ion |


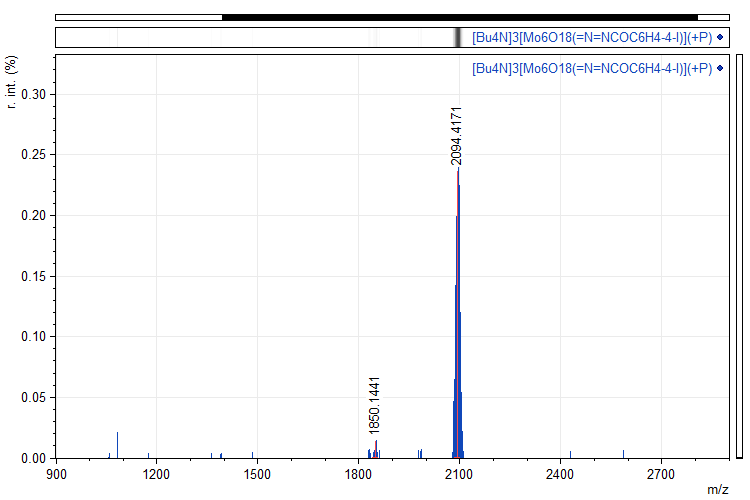


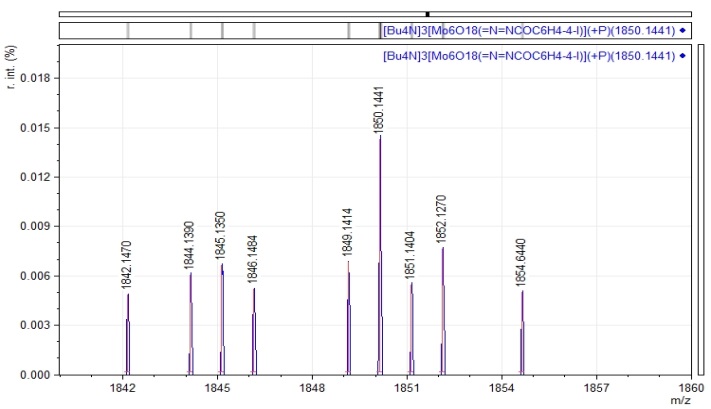

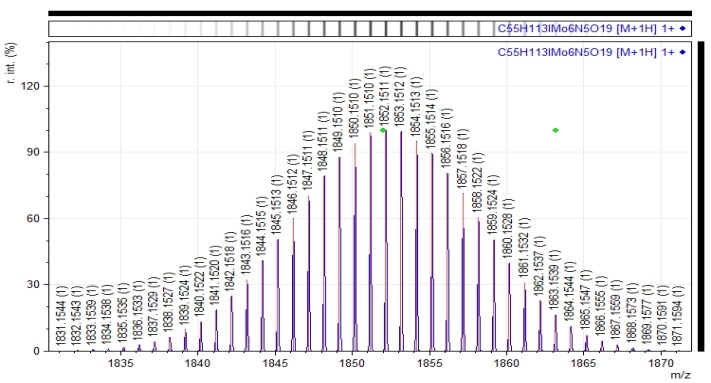


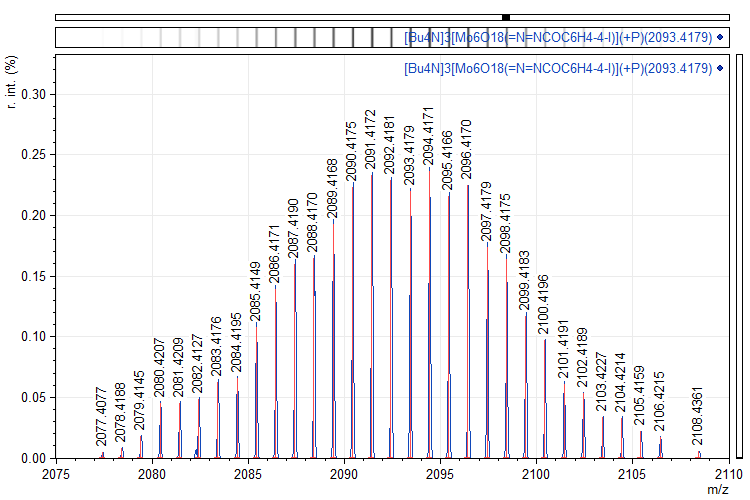

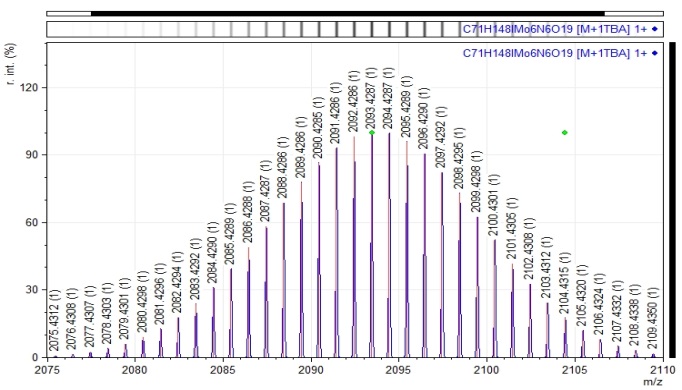


**Figure S31**. GC-HR MS spectrum (positive region) of [Bu_4_N]_3_[Mo_6_O_18_(=N=NCOC_6_H_4_-4-I)] (**3**): 1850.1441 for {M + 1H}^1+^, calcd. 1851.1510; 2093.4179 for {M + 1TBA}^1+^, calcd.2093.4287.


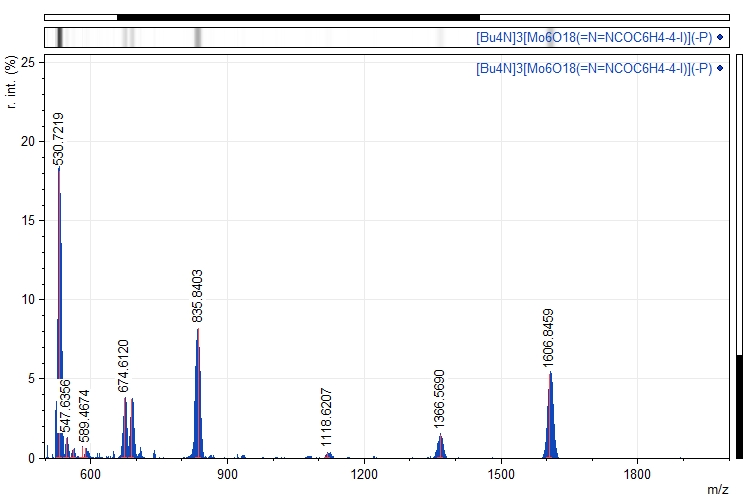


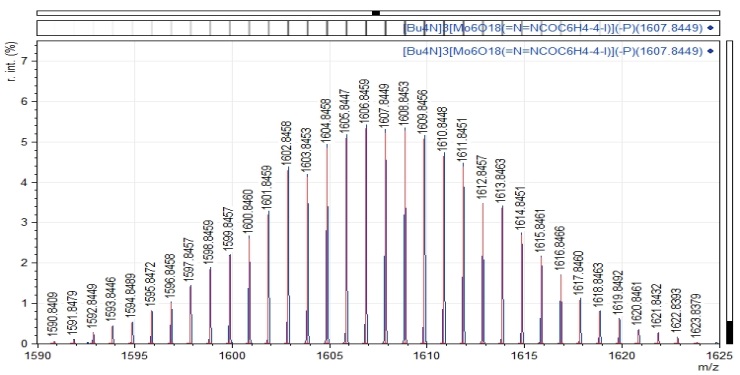

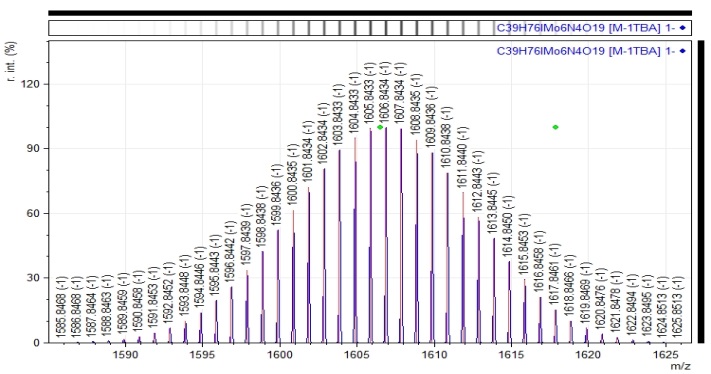


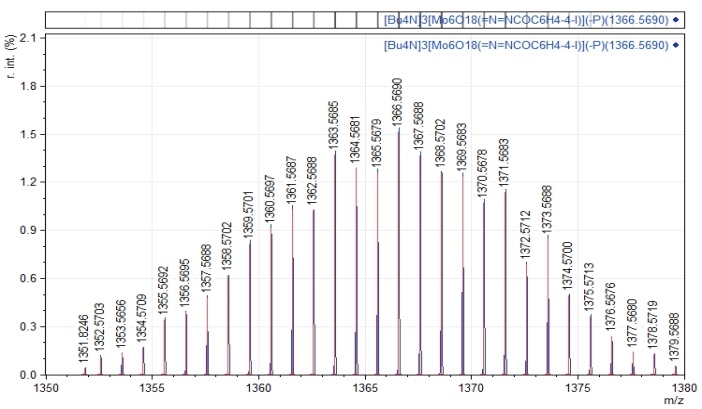

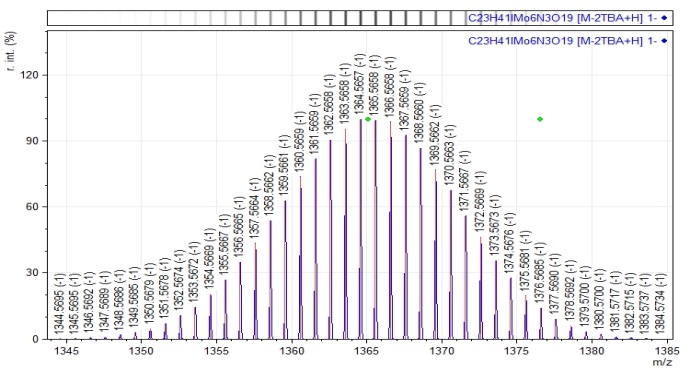


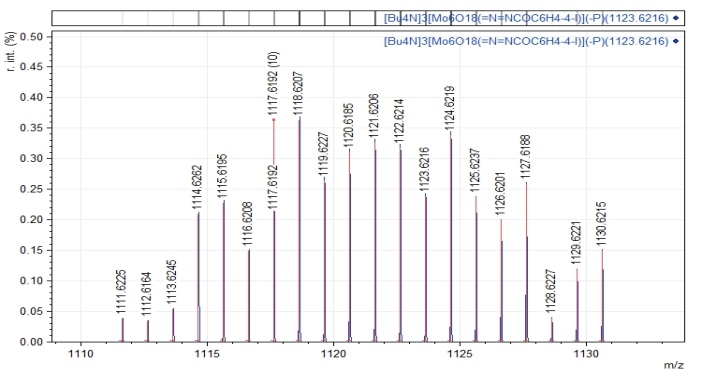

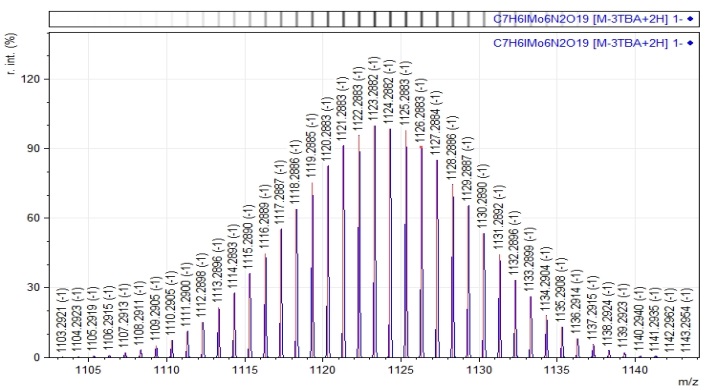


**Figure S32**. GC-HR MS spectrum (negative region) of [Bu_4_N]_3_[Mo_6_O_18_(=N=NCOC_6_H_4_-4-I)] (**3**): 1607.8449 for {M- TBA}^1-^: calcd. 1607.8434; 1366.5690 for {M- 2TBA + H}^1-^: calcd.1366.5658; 1123.6216 for {M- 3TBA + 2H}^1-^1123.2882.

Table S4 Summary of GC-HRMS results of [Bu_4_N]_3_[Mo_6_O_18_(=N=NCOC_6_H_4_-4-I)] (**3**).

| Negative mode of GC-HRMS for[Bu_4_N]_3_[Mo_6_O_18_(=N=NCOC_6_H_4_-4-I)] | | | | |
| --- | --- | --- | --- | --- |
| m/z | Calculated m/z | Assigned Species | Charge | Notes |
| 1607.8449 | 1607.8434 | {M- TBA}^1-^ | -1 | molecule ion |
| 1366.5690 | 1366.5658 | {M- 2TBA + H}^1-^ | -1 | molecule ion |
| 1123.6216 | 1123.2882 | {M- 3TBA +2 H}^1-^ | -1 | molecule ion |
|  |  |  |  |  |
| Positive mode of GC-HRMS for [Bu_4_N]_3_[Mo_6_O_18_(=N=NCOC_6_H_4_-4-I)] | | | | |
| 1850.1441 | 1851.1510 | {M + 1H}^1+^ | +1 | molecule ion |
| 2093.4179 | 2093.4287 | {M + 1TBA}^1+^ | +1 | molecule ion |


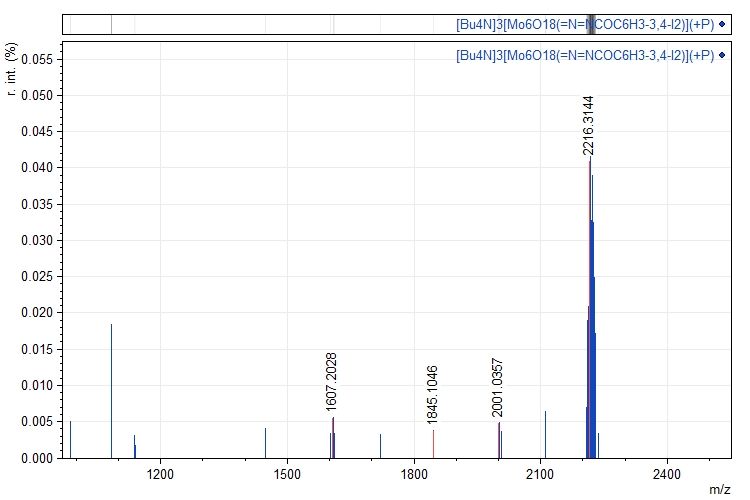


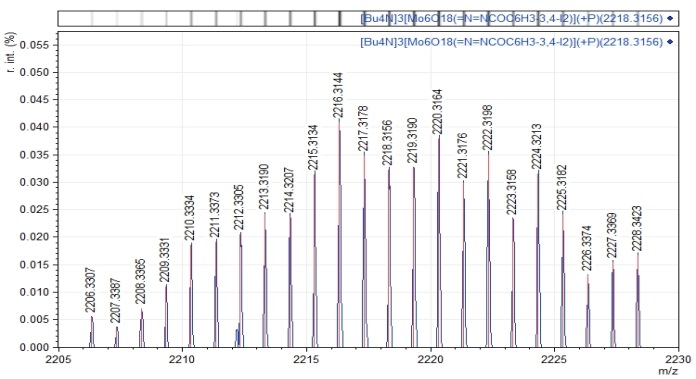

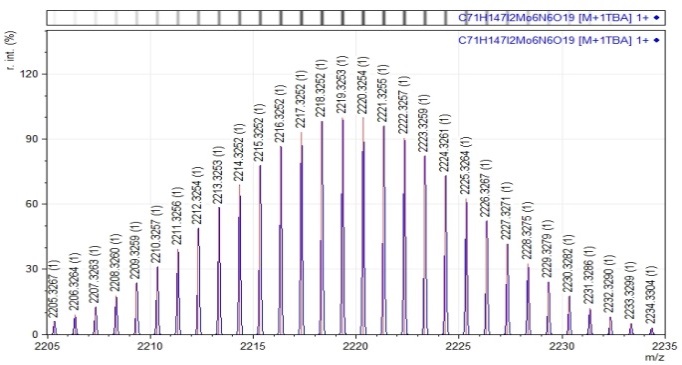


**Figure S33**. GC-HR MS spectrum (positive region) of [Bu_4_N]_3_[Mo_6_O_18_(=N=NCOC_6_H_3_-3,4-I_2_)] (**4**): 2218.3156 for {M + 1TBA}^1+^, calcd. 2218.3252.


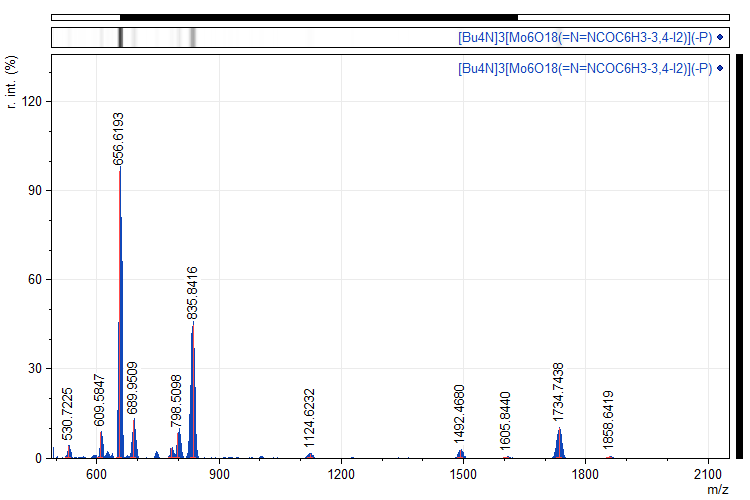


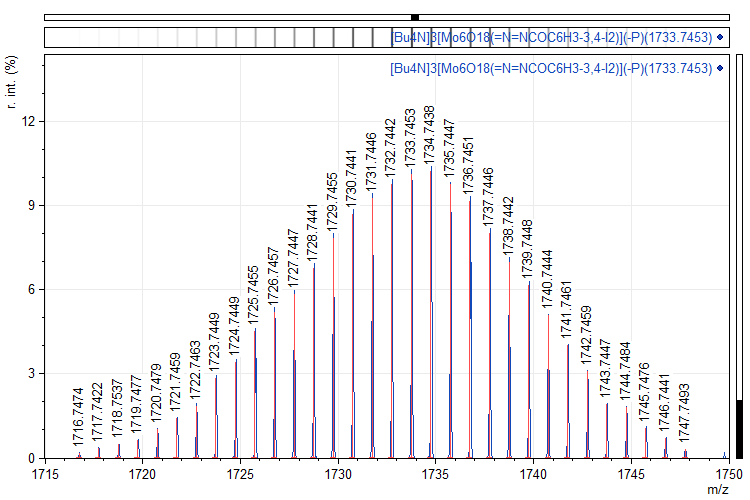

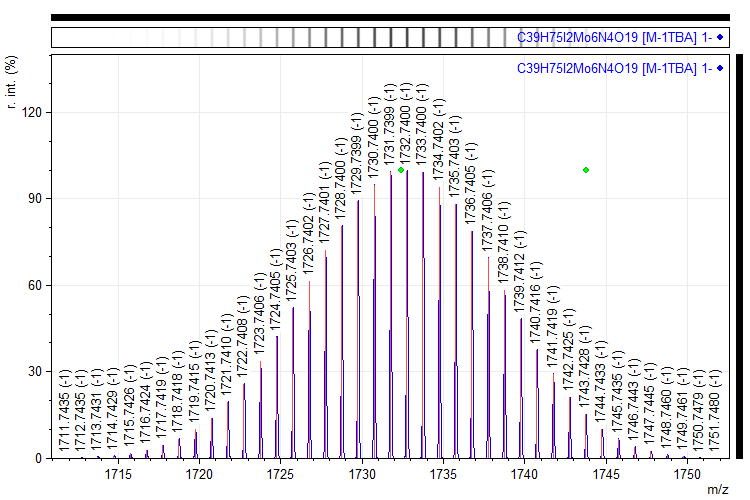


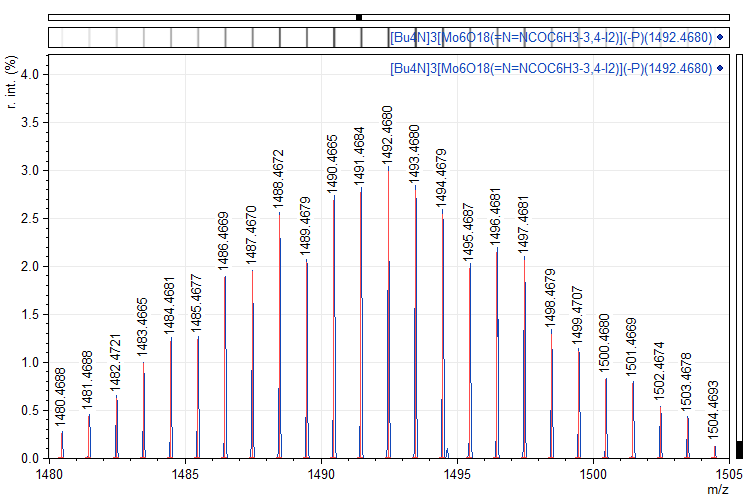

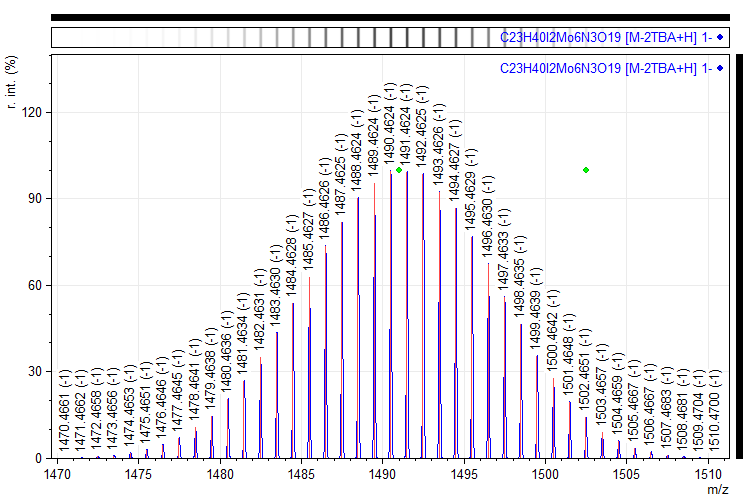


Figure **S34**. GC-HR MS spectrum (negative region) of [Bu_4_N]_3_[Mo_6_O_18_(=N=NCOC_6_H_3_-3,4-I_2_)] (**4**): 1733.7453 for {M- TBA}^1-^: calcd. 1733.7400; 1492.4680 for {M- 2TBA + H}^1-^: calcd.1492.4625.

Table S5 Summary of GC-HR MS results of [Bu_4_N]_3_[Mo_6_O_18_(=N=NCOC_6_H_3_-3,4-I_2_)] (**4**).

| Negative mode of GC-HR MS for [Bu_4_N]_3_[Mo_6_O_18_(=N=NCOC_6_H_4_-3,4-I_2_)] | | | | |
| --- | --- | --- | --- | --- |
| m/z | Calculated m/z | Assigned Species | Charge | Notes |
| 1733.7453 | 1733.7400 | {M- TBA}^1-^ | -1 | molecule ion |
| 1492.4680 | 1492.4625 | {M- 2TBA + H}^1-^ | -1 | molecule ion |
|  |  |  |  |  |
| Positive mode of GC-HR MS for [Bu_4_N]_3_[Mo_6_O_18_(=N=NCOC_6_H_3_-3,4-I_2_)] | | | | |
| 2218.3156 | 2218.3252 | {M + 1TBA}^1+^ | +1 | molecule ion |


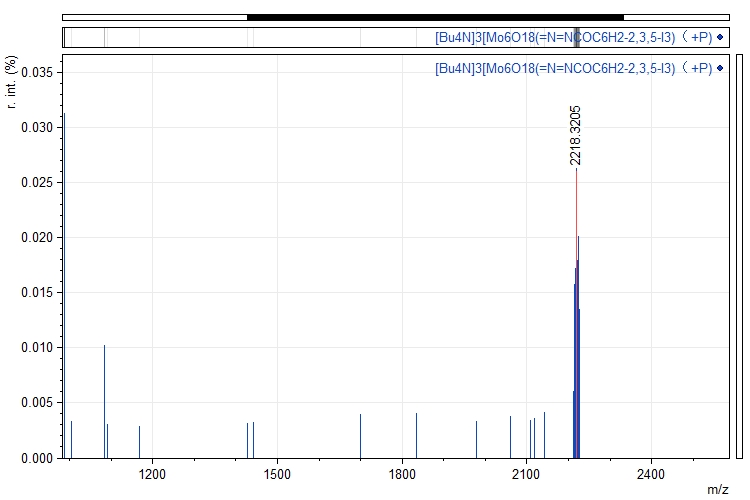

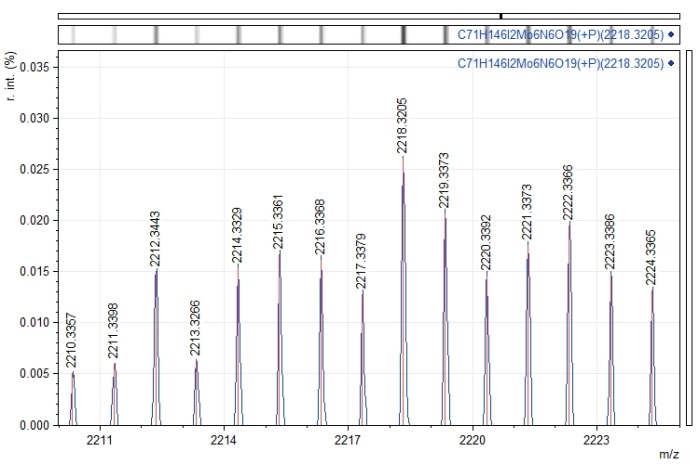

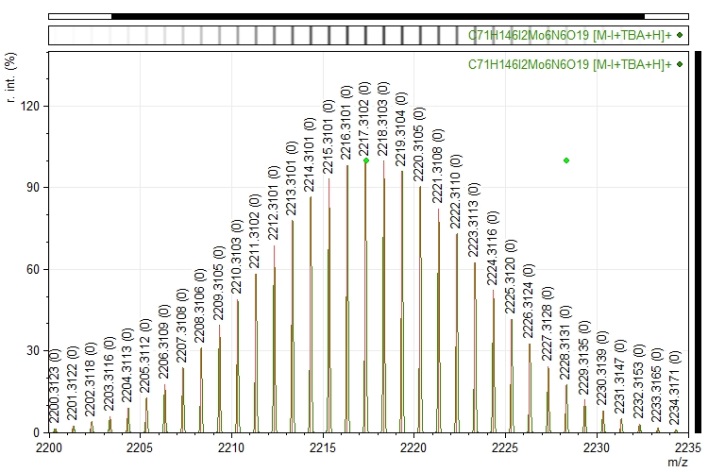


Figure **S35**. GC-HR MS spectrum (positive region) of [Bu_4_N]_3_[Mo_6_O_18_(=N=NCOC_6_H_3_-2,3,5-I_3_)] (**5**): 2218.3205 for {M – I + TBA + H}^+^ calcd. 2218.3103.


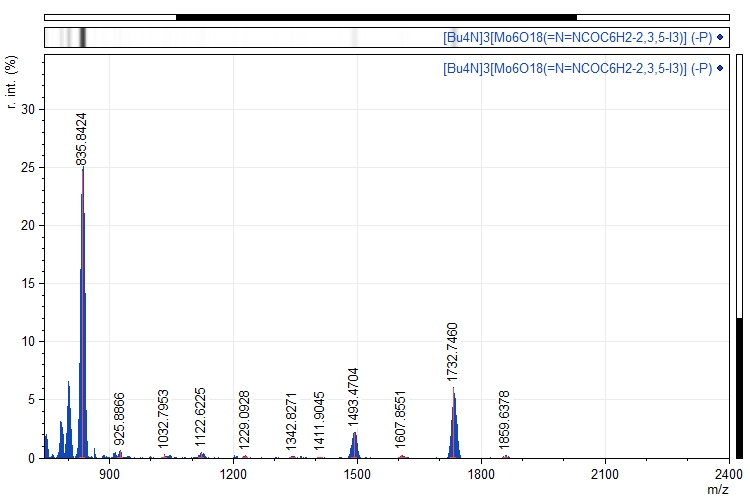


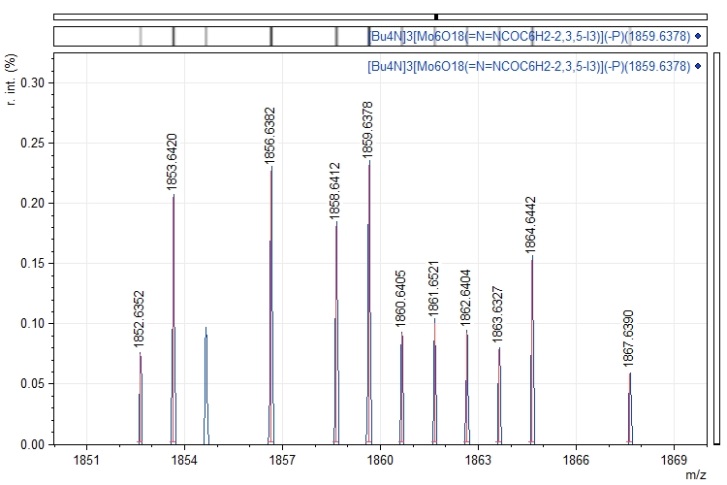

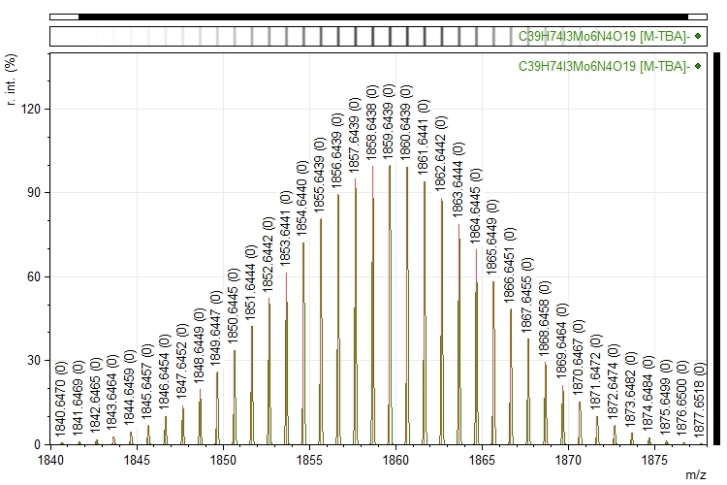


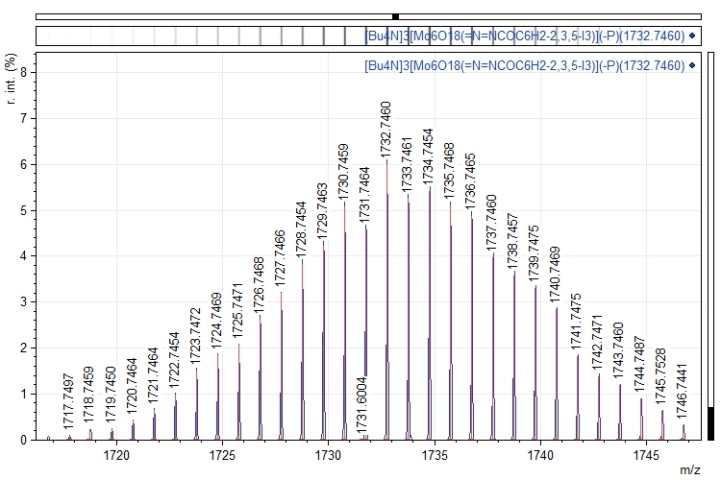

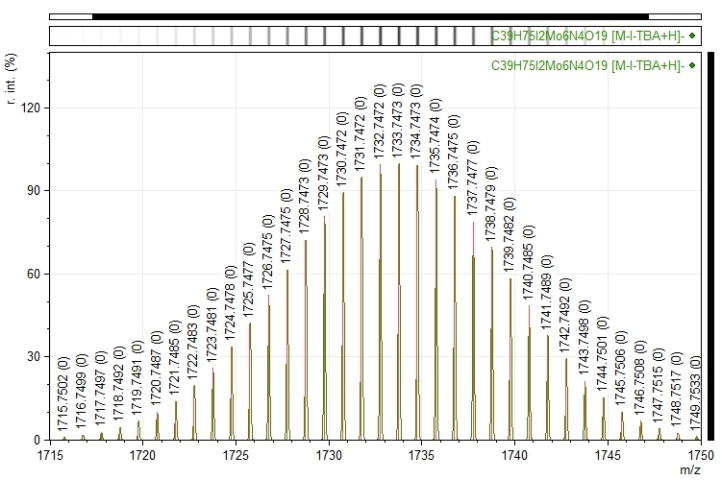


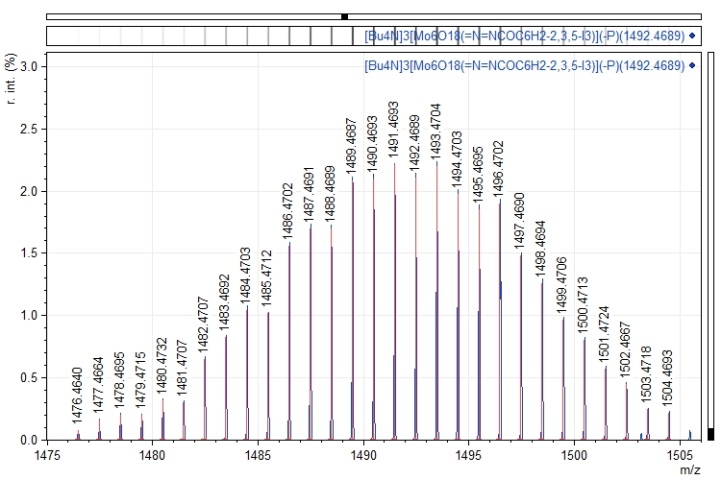

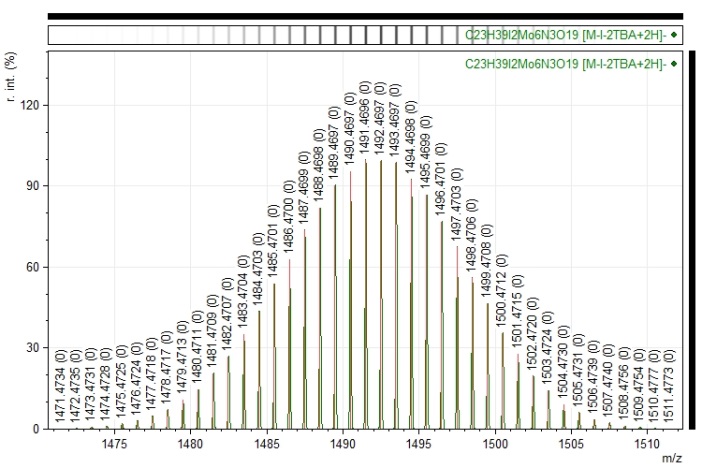


Figure **S36**. GC-HR MS spectrum (negative region) of [Bu_4_N]_3_[Mo_6_O_18_(=N=NCOC_6_H_2_-2,3,5-I_3_)] (**5**): 1859.6378 for {M- 1TBA}^1-^ calcd.1859.6439; 1732.7460 for {M-I-TBA+H}^1-^ calcd.1732.7472; 1492.4689 for {M-I-2TBA+2H}^-^  calcd.1492.4697.

Table S6 Summary of GC-HR MS results of [Bu_4_N]_3_[Mo_6_O_18_(=N=NCOC_6_H_2_-2,3,5-I_3_)] (**5**).

| Negative mode of GC-HRMS for[Bu_4_N]_3_[Mo_6_O_18_(=N=NCOC_6_H_2_-2,3,5-I_3_)] (**5**) | | | | |
| --- | --- | --- | --- | --- |
| m/z | Calculated m/z | Assigned Species | Charge | Notes |
| 1859.6378 | 1859.6439 | {M- 1TBA}^1-^ | -1 | molecule ion |
| 1732.7460 | 1732.7472 | {M-I-TBA+H}^1-^ | -1 | molecule ion |
| 1492.4689 | 1492.4697 | {M-I-2TBA+2H}^-^ | -1 | molecule ion |
|  |  |  |  |  |
| Positive mode of GC-HRMS for[Bu_4_N]_3_[Mo_6_O_18_(=N=NCOC_6_H_2_-2,3,5-I_3_)] | | | | |
| 2218.3156 | 2218.3252 | {M –I + 1TBA}^1+^ | +1 | molecule ion |

**References:**

Armarego, W. L. E. and Chai, C. L. L. (2003). Purification of Laboratory Chemicals, Fifth ed. Elsevier Science.

Hur, N. H., Klemperer, W. G. and Wang, R.-C. (1990). B. Tetrabutylammonium Octamolybdate*, Inorg. Synth.* 27, 78-79,

Mosmann, T. (1983). Rapid Colorimetric Assay for Cellular Growth and Survival: Application to Proliferation and Cytotoxicity Assays*, J. Immunol. Methods* 65, 55-63, doi:10.1016/0022-1759(83)90303-4.

Priti, K., Arvindhan, N. and Uchil, P. D. (2018). Analysis of Cell Viability by the MTT Assay*, Cold Spring Harb Protoc* 6, 469-471, doi:10.1101/pdb.prot095505.

Reed, L. J. and Muench, H. (1938). A simple method of estimating fifty percent endpoints*, Am J Hyg* 27, 493-497,

Wang, L., Yin, P., Zhang, J., Hao, J., Lv, C., et al. (2011). χ-Octamolybdate [Mo^V4^Mo^VI4^O_24_]^4-^: An Unusual Small Polyoxometalate in Partially Reduced Form from Nonaqueous Solvent Reduction*, Chem. Eur. J.* 17, 4796-4801, doi:10.1002/chem.201002154.
